# Agent-based models for detecting the driving forces of biomolecular interactions

**DOI:** 10.1038/s41598-021-04205-8

**Published:** 2022-02-03

**Authors:** Stefano Maestri, Emanuela Merelli, Marco Pettini

**Affiliations:** 1 School of Science and Technology, University of Camerino, 62032 Camerino, Italy; 2 Aix-Marseille Univ, Université de Toulon, CNRS, Centre de Physique Théorique, 163 Avenue de Luminy, 13288 Marseille Cedex 9, France

**Keywords:** Computer science, Computational biophysics

## Abstract

Agent-based modelling and simulation have been effectively applied to the study of complex biological systems, especially when composed of many interacting entities. Representing biomolecules as autonomous agents allows this approach to bring out the global behaviour of biochemical processes as resulting from local molecular interactions. In this paper, we leverage the capabilities of the agent paradigm to construct an *in silico* replica of the glycolytic pathway; the aim is to detect the role that long-range electrodynamic forces might have on the rate of glucose oxidation. Experimental evidences have shown that random encounters and short-range potentials might not be sufficient to explain the high efficiency of biochemical reactions in living cells. However, while the latest *in vitro* studies are limited by present-day technology, agent-based simulations provide an *in silico* support to the outcomes hitherto obtained and shed light on behaviours not yet well understood. Our results grasp properties hard to uncover through other computational methods, such as the effect of electromagnetic potentials on glycolytic oscillations.

## Introduction

Long-distance electrodynamic interactions between two small molecules have been largely studied within the framework of quantum electrodynamics, since long-range forces can be detected among excited atoms with similar transition frequencies^1,2^. However, interactions beyond the Debye screening length ($\simeq$ 10 Å in biological systems^3^), carried out by the molecular cognate partners of a biochemical reaction, are not well investigated. Nonetheless, experimental evidences for the existence of collective excitations of biological macromolecules are available in the Raman and Far-infrared spectroscopic domains^4,5^. Although long-distance electrostatic interactions have been considered unlikely, electrodynamic interactions, occurring between oscillating electric dipoles, might have a long-range nature; deterministic selective forces can thus be activated at a distance when the molecules undergo coherent collective oscillations^6^. The existence of forces of this kind might justify the efficiency of biochemical reactions more than the sole effect of stochastic short-range interactions, which rely just on Brownian diffusion and chemical affinity. Numerical studies proved that the overall interaction potential $\mathsf {U(r)}$ between cognate partners (with $\mathsf {r}$ being the intermolecular distance) is generally composed of a short-range term ($\mathsf {1/r}^{\mathsf{6}}$) and a resonant long-range term ($\mathsf {1/r}^{\mathsf{3}}$), meaning that, when the dipole moments of two molecules oscillate at the same frequency, an attractive resonant potential $\mathsf {U(r)} \sim \mathsf{r}^{\mathsf{-3}}$ should be added to the random Brownian force^7^.

These phenomena have been lately analysed, theoretically and experimentally, in the interactions among lysozyme molecules and oppositely charged dyes^8^. However, detecting long-range molecular recruitments in biosystems is still held back by the current technology; even these recent results, gained through Fluorescence Correlation Spectroscopy, are limited to systems where long-range interactions are built-in (by setting up a solution in which electrostatic interactions are non-screened).

Computational approaches might be able to overcome some of these hurdles, allowing to test *in silico* the existing theoretical models. Indeed, numerical simulations, such as those performed through Molecular Dynamics, have been already successfully carried out^8^, taking into account an a priori knowledge of numerous physical parameters characterising the molecular interactions under study. On the one hand, a model that considers the largest amount of empirical information allows generating a faithful representation of the biological system and provides a reliable *in silico* support for theoretical and experimental analyses; on the other hand, the lack of empirical data may limit the complexity of the system simulated.

**Figure 1:**
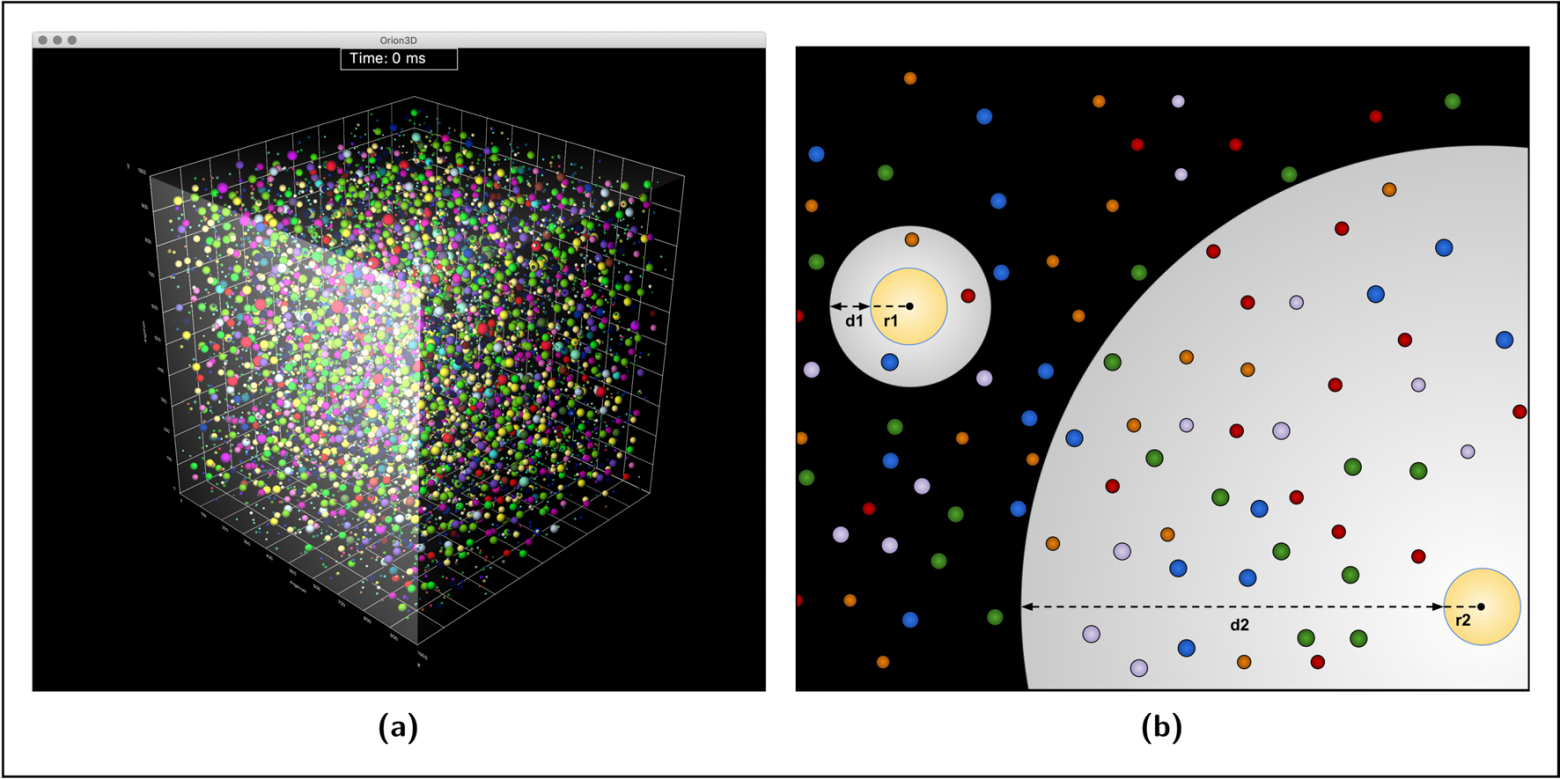
(**a**) Three-dimensional representation of the agents environment. Specifically, it is a one-attolitre cubic portion of cell cytoplasm, populated by enzymes and metabolites, each modelled as an autonomous agent and represented as a sphere. The figure is obtained from the 3D interface of the simulator we developed over our glycolysis ABM. It shows the position of every molecule instant by instant. The software also makes it possible to highlight the metabolites perceived by every enzyme at each time step of the simulation. (**b**) Graphical representation of the agent’s perception, by which every modelled enzyme detects the cognate metabolites in its surrounding environment. Each enzyme, depicted as a sphere of radius r, is able to perceive its neighbouring metabolites at different distances d. Such a process is fundamental to reproduce *in silico* the effects of the long-range forces on biochemical reactions, as will be discussed throughout this article.

This article aims to address most of these issues by exploiting an alternative way to define a computational model of molecular interactions in a metabolic pathway. Specifically, we construct an agent-based model (ABM) of a well-studied process, the glycolysis of yeasts, to simulate the effect of the long-distance electrodynamic interactions among the biomolecules involved in the pathway. Agent-based simulations make use of autonomous systems (agents) able to interact with one another in a concurrent and asynchronous fashion; they can thus fairly faithfully replicate *in silico* the behaviour of the entities interacting in a real biological system. ABMs require instructing the agents representing the simulated molecules with minimal empirical information, letting the global behaviour of the process result from local interactions, which are generated dynamically at each step of the simulation. The system evolves due to the ability of every agent to perceive and respond to the states of its environment, which is unpredictable and populated by other agents; the agent’s perception results in performing an appropriate action (if any) able to modify the environment^9^. The agent-based approach allows both the environment and the molecules to be three-dimensional (as shown in Fig. 1a); molecular shapes can thus affect the diffusion processes.

ABMs have been already successfully applied in the analysis of several biological systems and used to develop tools for *in silico* supporting experimental studies^10–12^. With the present work, we leverage the flexibility of the agent-based modelling to construct *in silico* biochemical systems; this approach is intended to simulate the glycolytic process by taking into account different types of forces driving molecular interactions. We aim to abstract the core features of biochemical systems characterised by purely random molecular encounters and compare them to those where cognate partners interactions are mainly driven by deterministic long-range forces. ABMs allow us to reproduce these phenomena in a network of mutually conditioning reactions without knowing a priori all the parameters needed in a numerical simulation, which might be missing or difficult to assay experimentally.

By analysing the concentration changes of the molecular species during each step of the agent-based simulation, we are able to hypothesise how long-distance interactions may quantitatively and qualitatively affect the glycolysis process. This way, we can also hint at what might be the physical phenomena underlying the related kinetic parameters if they would be assayed *in vivo* and highlight possible discrepancies with the values obtained *in vitro*. These results would provide the basis for setting up further experimental studies.

## Methods

### From a kinetic to an agent-based model

The construction of an ABM able to represent the molecular interactions of a metabolic pathway requires some information on the pathway itself and on the environment where it takes place. In particular, we need to know the sequence of reactions to simulate, or a subset of those relevant for our analysis, and some quantitative data, such as the initial concentrations of the species involved (as it will be better explained later in this section). In this perspective, a kinetic model can serve as a source of such data and as a reference against which to compare our results.

We cannot completely base our study on a kinetic model, since it uses experimental parameters, often assayed *in vitro*, to directly describe the global properties of the system through a set of differential equations. Conversely, we aim to understand if kinetic data actually underlie processes related to the ability of molecules to perceive each other, even from a long distance. An ABM of molecular interactions allows not considering a priori some of these parameters and thus provides a better baseline over which carrying out our *in silico* study. ABMs describe molecular interactions at a local level, but they also possess compositionality, that is, the capability of recursively applying the rules characterising agents interactions to define progressively higher abstraction levels. In this way, we can hide the unnecessary details of a specific level and, at the same time, observe its global behaviour^13,14^. Considering the case of a metabolic pathway, a kinetic model treats enzymatic reactions as mathematical functions that relate the concentrations of reactants to those of products, assuming that they incorporate the role carried out by each molecular interaction. In our ABM, instead, each enzyme is represented by a dedicated agent able to perceive the environment and its cognate partners; the interactions among the molecules are thus explicit in the definition of the model. The compositionality of ABMs also makes it possible to conduct the study at an abstraction level that can be represented with a small amount of empirical data, without losing accuracy in reproducing macromolecular behaviours.

Nonetheless, not all the kinetic parameters can be overlooked: in order for a modelled saturated enzyme to generate the products of the reaction, faithfully to its biological counterpart, it must wait for a time interval corresponding to the reciprocal of its $k_{cat}$ value (or turnover number). The $k_{cat}$ represents the number of molecules converted by an enzyme in the time unit; therefore, its reciprocal provides the interval after which the reaction products are released in the environment, and the enzyme returns free.

**Figure 2:**
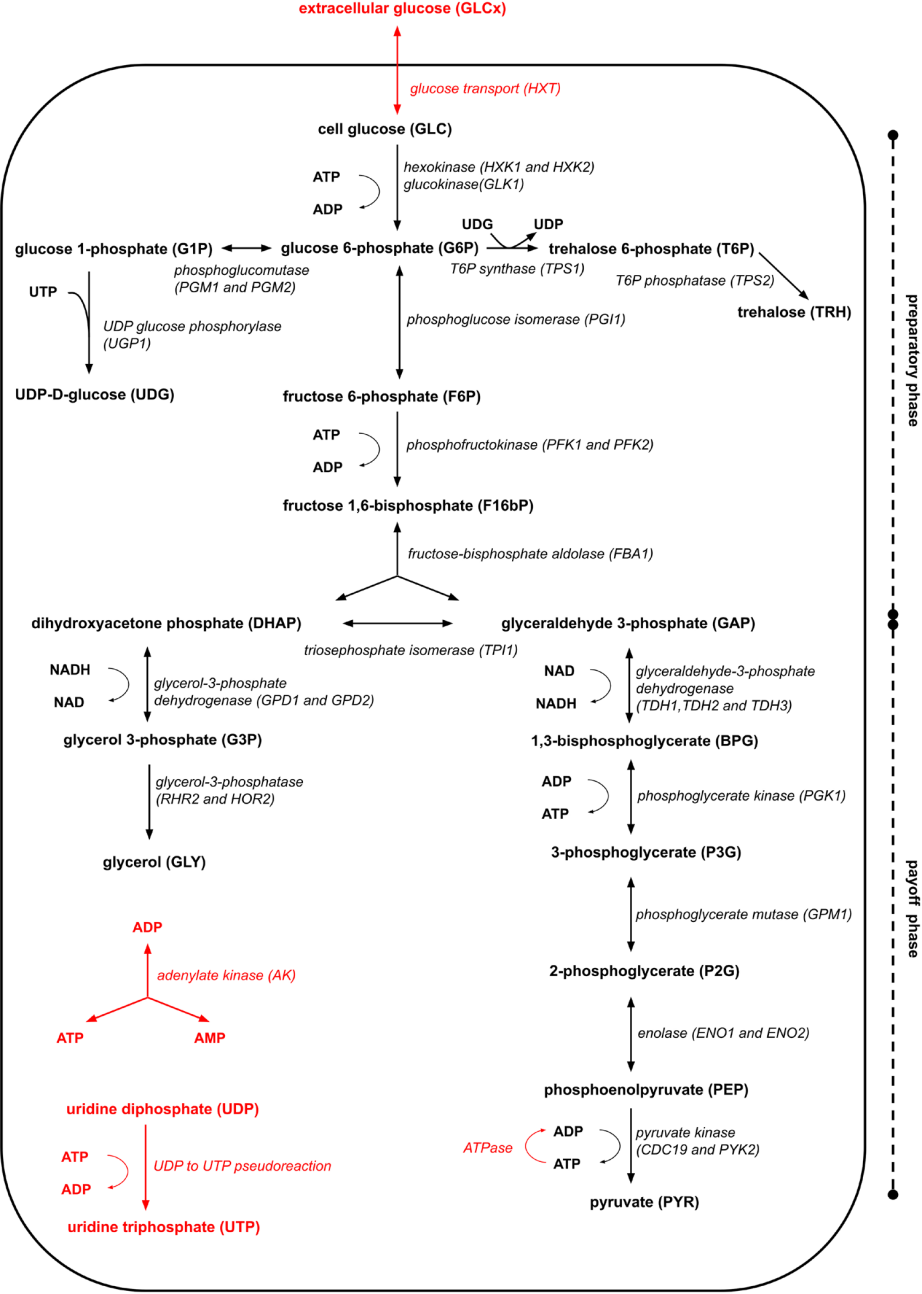
Schematic representation of the glycolysis steps and branches taken into account in our ABMs. They are extracted and adapted, through a dedicated module of Orion, from the SBML of the Smallbone2013 kinetic model^16^. The reactions in red are those excluded during the conversion; see the “Methods” section of this manuscript and Sect. 2 of the Supplementary Information for details on the conversion process from the SBML source to our ABM. For each metabolite involved, we report both the name and the acronym (in bold), while, for every reaction, we indicate the abbreviation of each isoenzyme carrying it out (in italics). On the right side of the image, we highlight the two main phases of the process; since the ethanol fermentation has not been simulated, we prefer not to show this phase to preserve the readability of the figure.

Several kinetic models have already been constructed over metabolic pathways, mainly because the properties of metabolism at steady state simplify the model definition^15^. However, by considering the enzymatic reactions as just mathematical functions from reactants to products, they mostly focus on changes in metabolite concentrations and do not provide the actual number of enzyme molecules in the simulated environment. In contrast, for the reasons explained above, this information is fundamental for constructing our ABM. Based on this requirement, we identified in the “Smallbone2013 - Iteration 18”^16^ a model particularly suitable to serve as a source for the ABM, since it contains a complete set of experimental data on the isoenzymes involved in a well-studied metabolic process, the glycolysis of Saccharomyces cerevisiae. The Smallbone2013 model provides a detailed description of the chain of reactions that generates energy from glucose by breaking it into two molecules of pyruvate. In addition to the main branch of glycolysis, it includes the glycerol, glycogen and trehalose branches and also considers the alcoholic fermentation steps, which lead to the formation of ethanol (see Fig. 2). It, therefore, defines a system of interacting molecules sufficiently complex to allow bringing out, through an ABM, the global effect of the long-range forces on the dynamics of the pathway.

### Designing the model of glycolysis

The ABM is the basis for a simulator whose input is in the form of an SBML (Systems Biology Markup Language) file filled with experimental data^17^. It contains information about the molecules involved in the metabolic pathway and their initial concentrations; data related to the reactions carried out are also taken from this source. The Smallbone2013 model is represented in SBML format.

A dedicated module of the simulator converts the SBML model to an XML (Extensible Markup Language) file specifically formatted to be interpreted by the simulator itself but also to be human-readable^18^. Therefore, its main function is to translate the kinetic representation of the metabolic reactions into our agent-based model. To do this, for every reaction in the SBML model, it gets the reactants and products, and generates XML code for each of its interactions. It also associates, to each modelled molecule and reaction, the physical and kinetic parameters needed to set up the simulation; for the study proposed in this paper, they are limited to the molecular weights (automatically retrieved from online databases, such as UniProt^19^ and ChEBI^20^) and the turnover numbers. Our agent-based simulator can also deal with a higher level of detail, obtained by forcing an enzyme to form a complex with an encountered cognate metabolite on the basis of a priority list constructed over the $k_{cat}/K_m$ ratio (specificity constant); this possibility can be established during the initial setup of the simulation and requires the user to provide the $K_{m}$ values of each enzyme-substrate interaction (see Sect. 2.2 of the Supplementary Information). Despite that, with the current study, we want to leverage the capability of agent-based simulations to reproduce the collective properties of a biological system drawing from a small amount of empirical data; we thus simulate the molecular interactions as completely random, without predetermining any priority on the metabolites perceived by an enzyme. A generalisation of the XML input and its construction via the dedicated module of the simulator are described in the Supplementary Information; this document also contains the actual XML listing generated for running the simulations.

By importing the reactions of the SMBL file as the input of our agent-based simulations, we excluded all those for which the Smallbone2013 model does not provide the enzymatic concentrations. Our simulator can actually handle this kind of reactions, since we can model them in terms of their bulk effects; however, for the aim of observing the global behaviour of glycolysis as resulting from the local molecular interactions, introducing any bulk reaction would perturb the environment and hide the absence of actual interactions among the molecules, making the ABM close to a standard kinetic model. Based on this idea, we do not consider the *adenylate kinase, ATPase*, and *UDP to UTP* reactions and *glucose transport* (between the extracellular environment and the cytosol). The most significant of these reactions is the adenylate kinase, since it controls the ratio of ATP, ADP and AMP (also called energy charge), which in turn affects the allosteric regulation of important enzymes, such as phosphofructokinase and hexokinase^21^. However, the length of the simulated process (1 second, as better discussed in the “Results” section) makes the allosteric regulation and the whole energy charge effects negligible^22,23^. Suppressing glucose transport and enzyme regulation also prevents, de facto, the achievement of a steady state, helping us to emphasise the effects of the various types of interactions on the concentration changes in the simulation interval.

The initial concentrations of the molecular species are gained from the SBML file as millimoles per litre (mmol/l). The simulator’s module mentioned above converts these values into the initial particle numbers, needed to instantiate the agents at the beginning of the simulation. In this regard, we point out that, although agent-based simulations have a fairly light computational load, reproducing a metabolic pathway involves thousands of molecules, and therefore as many agents running concurrently. The resulting resources demand conditioned the molecular concentrations we were able to simulate. More precisely, we scaled the concentrations provided by the Smallbone2013 model to values less than 1 mmol/l. Indeed, the Smallbone2013 model provides a wide range of species concentrations (e.g., from the 6.28 mmol/l of glucose to the 0.0007 mmol/l of 1,3-bisphosphoglycerate); scaling them just proportionally to the simulated volume (1 attoliter, as clarified later in this section) would have caused the species with lower concentrations to disappear from the system. Adapting the concentrations provided by the Smallbone2013 model to values less than 1 mmol/l is a first approximation to meet the limits in the computational power. Future development of the simulator will implement distributed computing to deal with concentrations more faithful to experimental data. In Supplementary Table S1, we report the initial concentrations of all the simulated species.

Our agent-based model is intended to study the glycolytic pathway from the general perspective of the oxidation of one molecule of glucose to two molecules of pyruvate; for this reason, we consider the pyruvate as the end product of the process and excluded the fermentation-related reactions, catalysed by the pyruvate decarboxylase isoenzymes (PDC1, PDC5, PDC6) and by the two alcohol dehydrogenase isoenzymes (ADH1 and ADH5). Therefore, the branches acting on pyruvate, that is, the succinate and acetate branches of glycolysis, are not taken into account in our model.

To complete the list of changes we made to the original kinetic model, we report that, according to most of the literature, we modelled the reactions catalysed by hexokinase (and glucokinase), phosphofructokinase, and pyruvate kinase as irreversible^24–26^, since they function as control points of the whole glycolysis process, despite the Smallbone2013 model considers irreversible only the reaction performed by phosphofructokinase.

The subset of reactions characterising the model at the basis of our simulations, as resulting from the above-described adaptations, can be found in Fig. 2 of this manuscript and in Supplementary Table S2.

### Orion: agent-based simulator for metabolic pathways

The study proposed in this manuscript has been carried out with the aid of Orion, a spatial simulator for metabolic pathways. It has been developed in Java starting from a prototype project^27,28^. In what follows, we introduce the basic principles over which the simulator has been designed; this information can help the reader to better understand the results of our work. Further details on modelling and implementation choices are provided in Sect. 2 of the Supplementary Information.

Orion is a agent-based simulator, this means that the molecules involved in the pathway are represented by agents, autonomous systems able to perceive changes in their environment and react to them. Formally, a reactive agent is defined by a 6-tuple $\langle E, Per, Ac, see, action, do \rangle$ where:

- *E* is the set of all states for the environment
- *Per* is a partition of *E*
- *Ac* is a set of actions
- *see*: $$E \rightarrow Per$$
- *action*: $$Per \rightarrow Ac$$
- *do*: $$Ac \times E \rightarrow E$$

*Per* represents the perception of the environment from the agent’s point of view. An agent observes the environment (*see*), selects the appropriate action (*action*), and acts (*do*) on the environment itself^9^.

The simulations are performed in the three-dimensional space, representing a portion of the cytoplasm, that is, the environment perceived by the agents. Each molecule is modelled as a sphere, whose radius is estimated from its molecular weight and the average value of the molar specific volume of a protein in solution^29–31^. These modelling choices produce a fairly realistic molecular crowding in the simulated portion of the cytoplasm. Moreover, by making every molecule spherical, we can correlate its shape to its diffusion coefficient through the Stokes-Einstein equation for the Brownian motion of a spherical particle:

1 $$\begin{aligned} D = \frac{k_B T}{6\pi \eta r} \end{aligned}$$

where $k_B$ is the Boltzmann constant, *T* the temperature, $\eta$ the viscosity of the environment, and *r* the radius of the molecule. For our simulations, we set $T = 298.15$ kelvin and $\eta = 0.0011$ pascal-second.

Each molecule can freely move, inside the simulation volume, according to a vector applied to the centre of its sphere: its direction is generated randomly, based on polar coordinates, while its module is calculated from the ambient diffusion coefficient *D*, obtained via Equation 1, as the average value of the square of the molecule displacement *x* in a time *t*:

2 $$\begin{aligned} <x^2>\,= 2Dt \end{aligned}$$

A dedicated agent monitors the position of all the molecules to ensure that every movement ends in an empty space of the environment, avoiding collisions and overlaps.

The simulator enables to set the space unit and the time scale as per requirement; in this study, we consider the angstrom (Å, equivalent to $10^{-10}$ m) for space and $10^{-4}$ seconds for time (corresponding to one tick of the simulation clock). A cube of 1 attolitre ($10^{-18}$ L, having a side of 1000 Å) represents the best option for the aim of our analysis and meets the computational demand of the simulations (Fig. 1a shows the 3D space visualised through the interface of the simulator).

The model at the basis of the simulator classifies molecules into three types: *enzymes*, *complexes,* and *metabolites*; the latter can only move inside the simulation volume, while the first two classes of molecules can also act on the environment.

Although molecular movements are modelled on the basis of Brownian diffusion, this study pushes forward the capabilities of the agent paradigm by not limiting molecular interactions to just those allowed by random encounters. Indeed, enzymatic reactions are simulated by exploiting the ability of agents to perceive and interact with one another: each enzyme identifies the cognate metabolites in its proximity thanks to a perception-sphere that it projects on the environment. Such an approach is the simulator key feature that allows studying the effects of long-distance interactions among biomolecules; for this reason, it will be detailed in the “Results” section.

Every molecular interaction may lead to the formation of a complex, which is modelled in the ABM as a new agent. If such a complex represents a saturated enzyme, it waits an amount of time corresponding to the reciprocal of its $k_{cat}$ value and then releases the final product (or products) of the reaction; otherwise, it moves and acts on the environment to bind the metabolite needed to reach saturation. This modelling approach is based on the construction of a *reaction automaton*, introduced in a previous work^32^ and formalised in Sect. 2.1 of the Supplementary Information.

## Results

### Modelling short- and long-range forces among biomolecules

To simulate the effects of the molecular interactions operating at different distances, we endowed agents with specifically designed perception capabilities. Their core property lies in the definition of a *perception sphere* that surrounds each active molecule (enzymes and complexes, as explained in the “Methods” section). By setting the *perception radius*, that is, the radius of the perception sphere, we can model various lengths at which enzymes and complexes are able to interact with their cognate partners. Therefore, *the capability of agents to perceive and interact with one another allows us to abstract the effects of the electrostatic and electrodynamic potentials among the molecules of the simulated environment*; this can be achieved without taking into account all the physical parameters usually required in molecular dynamics simulations (such as the potential values or the forces generated by molecular collisions)^8^.

Each perception radius is obtained by summing the radius of the enzyme to the *perception distance* at which we want the enzyme to be able to find a cognate metabolite; the perception distance extends beyond the surface of the sphere representing the enzyme. As the distance of the metabolite from the enzyme increases, the intensity of the forces acting on it diminishes; for this reason, each perception sphere is characterised by different interaction probabilities, depending on its size (more details on the perception spheres implementation are provided in Sect. 2.4 of the Supplementary Information).

We simulated three different systems, in which the interactions characterising the glycolytic process are driven by the specific kinds of forces whose effects on the pathway we aim to compare. Going into details, the agent-based modelling approach makes us able to define:

- A system in which molecular encounters are driven only by Brownian motion and dynamic complementarities (e.g., lock-and-key or induced-fit phenomena). We modelled this system allowing enzymes and complexes to identify a cognate metabolite within a *perception distance of* *5 Å*; this sets the space on which electrostatic forces, such as those resulting from van der Waals-like potentials, operate.
- A system where a *10 Å* *perception distance* models the effects of electromagnetic potentials limited by the Debye screening^3^; it restricts the interactions to just those allowed by stochastic short-range forces.
- A system characterised by *perception distances of* *300 Å*, chosen as the average length to simulate the existence of long-range electrodynamic forces among biomolecules (considering that the size of the simulation volume of our study is 1000 cubic angstroms). As mentioned in the Introduction, these are deterministic attractive forces activated by a long-range potential between two dipolar molecules A and B, if they vibrate at frequencies $\omega _A \simeq \omega _B$ (that is, if they are at resonance). In real cells, this phenomenon might be observed because a macromolecule oscillating at a high frequency (in the range of $10^{10} - 10^{11}$ Hz) does not suffer the Debye screening effect by the ions of the medium^6,7^.

In Fig. 1b, we provide a graphical representation of how the perception radii project on the environment. Each of them is given as the sum of the enzyme radius (r) and the perception distance (d) at which the molecule is able to detect its cognate metabolites. The perception radius of the r1+d1 type schematises the constraint that limits the enzyme interactions to those allowed by short-range forces (both *5 and 10 Å* *perception distances*), while a r2+d2 type radius models the effects of the long-distance electrodynamic interactions. We point out that the figure arranges side by side two different types of radii just for comparative purposes; in the ABMs defined for glycolysis, *only one type of radius is allowed per modelled system*.

Imposing fixed perception distances for each simulated system may be considered a limitation, since a molecule involved in multiple interactions can be affected by different kinds of electrodynamic forces. However, to define the glycolysis ABM, we need to balance the accounted systems’ details with the availability and reliability of experimental data; nonetheless, we have to consider the computational cost of the simulations. Modelling different radii for each agent (in the same simulation) would imply taking into account, a priori, a high number of physical parameters that are currently unknown (i.e., the relative strengths of different interactions). We experienced a similar problem with Molecular Dynamics simulations; the aim of our agent-based approach is, indeed, to assess the possible presence of long-range molecular interactions in a metabolic pathway by relying on few empirically known parameters.

### Outcome of the simulations

By setting the local rules that determine movements and interactions of the molecules involved in our model of yeast glycolysis (as detailed in the “Methods” section), the global behaviour of the pathway can be observed, during the simulation, in the form of molecular concentration changes (mmol/l) over time (s).

To balance the computational demand of dealing with thousands of molecules and the need of producing worthwhile outputs, we run each type of simulation for an interval of 1 second (about ten days of actual running time); it turned out to be sufficiently long for us to observe and compare the specific features of each of the three modelled systems.

**Figure 3:**
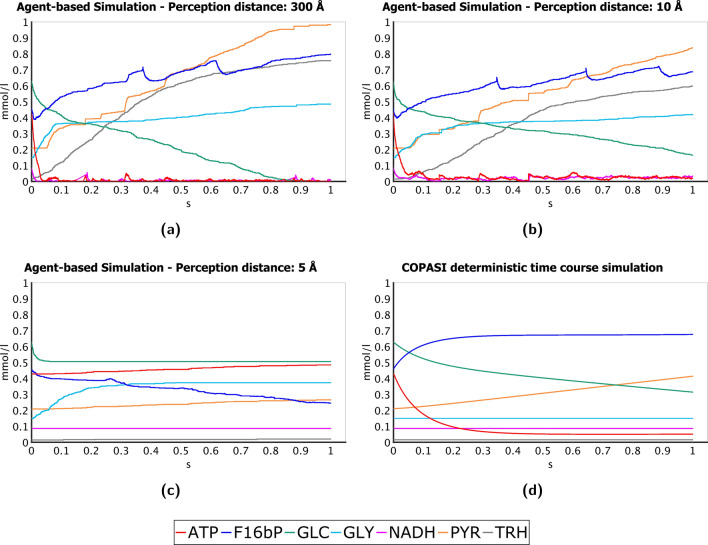
Concentration changes over time, in simulations of 1 second, of a selection of metabolites particularly relevant for our study (for the complete set of plots, representing all the metabolites simulated, see Sect. 3.1 of the Supplementary Information). Through this figure, we provide a comparison of the plots generated by three agent-based simulations–with perception distances set to 300 Å (**a**), 10 Å (**b**) and 5 Å (**c**), respectively–and by a deterministic time course simulation based on the Smallbone2013 kinetic model (**d**). The selected metabolite species are: glucose (GLC), the source of the glycolytic pathway; pyruvate (PYR), NADH, and ATP, that is, the end products of glycolysis; trehalose (TRH) and glycerol (GLY), respectively, the products of the two main glycolysis branches; fructose 1,6-bisphosphate (F16bP), the product of the most important control-point reaction of the glycolytic pathway, namely the one catalysed by the phosphofructokinase. In plot (**a**), it is possible to notice how the simulation that takes into account long-range electrodynamic forces (300 Å perception distance) also shows a higher reactivity and an evident increase in the amounts of the pathway end products. In comparison, the simulation that limits the electromagnetic forces to those affected by the Debye screening (10 Å perception distance), shown in (**b**), is not able to consume all the glucose in the environment and generates significantly smaller amounts of pyruvate and thralose. Simulating a system driven by van der Waals-like potentials (5 Å perception distance), whose plot is represented in (**c**), causes negligible changes in metabolite concentrations and the glucose consumption reaches a plateau; the agent-based approach allows us to attribute this behaviour to the inability of the reactions that use ATP or NADH as energy donor to bound these types of metabolites (see the “Results” section for further details). The plot (**d**) is generated through the deterministic time-course simulation of the Smallbone2013 model using the software Copasi^33^.

In Fig. 3, we report some of the concentration changes characterising each type of system. For generating these plots, we selected the metabolites whose amount variations during the simulation have been accounted as the most meaningful for our analysis (a complete set of plots, covering all the metabolite species considered in our models, is provided in Sect. 3 of the Supplementary Information).

The simulation performed by setting *perception distances of* *300 Å*, which represents a system where we hypothesise the existence of selective long-range molecular recruitments, has the highest reactivity and efficiency (Fig. 3a); already after 0.9 s, all the glucose in the environment is consumed and the pyruvate (one of the main products of the pathway) increases from an initial concentration of 0.2 mmol/l to about 1 mmol/l.

In the system where we limited the electromagnetic forces to those below the Debye screening (*perception distances of* *10 Å*—Fig. 3b), we do not observe utterly different concentration changes in comparison to the previous one; however, they clearly show a lower efficiency in the production of pyruvate from glucose, which a system of that type is unable to completely deplete in the chosen simulation interval.

These two types of simulations can also be compared in terms of variations of the other main products of glycolysis and related branches. Indeed, they both report a clear, yet similar, increase of the glycerol amount. Conversely, if we take into account the effects of long-distance interactions, the trehalose branch shows a change of its end product from 0.015 to 0.76 mmol/l, a concentration 56% higher compared to the 0.48 mmol/l resulting from a system limited by a 10 Å perception distance. Regarding ATP and NADH, their concentrations reach almost immediately a value close to zero and then oscillate around it during the entire simulation. This behaviour is observable due to the short interval of glycolysis we are analysing: at this stage of the process, the reactions that use ATP as an energy donor, as well as the redox conversion of dihydroxyacetone phosphate to glycerol 3-phosphate, which is coupled with the oxidation of NADH, still have an abundance of substrate to consume; as a consequence, the related enzymes continuously bind the ATP and NADH in the environment to perform their catalytic activity.

We obtained remarkably opposed results in the case of simulations based on *perception distances of* *5 Å*, which model a system affected only by short-range van der Waals-like potentials (Fig. 3c). Despite the certainty that a metabolite will be bound by its cognate enzyme when it enters a such a small perception sphere (as detailed in Sect. 2.4 of the Supplementary Information), at the end of the simulation, we can observe negligible increases in the concentration of the pathway end products as well as in the consumption of glucose. In particular, the curve representing the latter reaches a plateau after a small depletion in its concentration, a behaviour we would observe at steady state; however, mostly because we did not implement enzyme regulation and glucose transport, such a condition is unlikely in our simulated systems. We can also observe similar concentration changes for glycerol (in this case, it increases before reaching a plateau). In both the situations, these anomalous behaviours are explainable if we observe the curves of ATP and NADH: the amount of ATP never decreases, because, during the preparation phase of glycolysis, neither the hexokinases nor the phosphofructokinases are able to bind this molecule and complete the catalysis of their respective reactions. Indeed, glucose molecules are bound at the beginning of the simulation, but then the environment maintains the same concentration of hexokinase-glucose and glucokinase-glucose complexes for the entire simulated interval (plots reporting the concentrations changes of complexes are provided in Sect. 3.2 of the Supplementary Information). This phenomenon also justifies why fructose 1,6-bisphosphate (product of the phosphofructokinase) can only decrease, consumed by fructose-bisphosphate aldolase. NADH, instead, remains stable at its initial concentration of 0.086 mmol/l, because the glycerol-3-phosphate dehydrogenase is not able to bind it; when all the glycerol 3-phosphate in the environment has already been converted in glycerol, the latter can no longer be produced (causing the observed plateau of its curve).

We compared the results described so far with the output obtained through a numerical time-course simulation; this has been performed via the Copasi software^33^ over the Smallbone2013 model^16^. We modified the original SBML with the same changes applied to our ABM, relating to accounted reactions and initial molar concentrations (see the “Methods” section). However, we left unchanged the functions associated with enzymes regulation, because a system of differential equations resulted in being less flexible than an ABM and removing this feature would have compromised its consistency, making the numerical simulation impossible. As shown in Fig. 3d, the kinetic model thus generates results closer to a steady-state condition, a property that, at first glance, may mislead the observer to find analogies with the simulations accounting 5 Å perception distances. However, excluding the fluctuations of metabolites concentrations, which are better captured by the ABM and more evident in the related plots, most of the shown concentration changes are loosely similar to those identified when we simulated 10 Å and 300 Å perception distances. This can be verified at least for the consumption of glucose and ATP, and for the increase of pyruvate and fructose 1,6-bisphosphate; nonetheless, they show significantly smaller variations from their initial molar concentrations. Although we consider identifying such properties in some of the most relevant species of the pathway noteworthy, we also point out that the last observations do not apply to all the simulated metabolites (as explained in Sect. 3.1 of the Supplementary Information).

## Discussion

The outcomes of the agent-based simulations detailed above suggest that the two systems reproducing an off-resonance situation, where molecular interactions rely only on van der Waals-like potentials or, at least, on electromagnetic forces shorter than the Debye length, are not able to oxidise glucose at a high rate. This property is particularly true when we limit the *perception distance* to 5 Å, resulting in negligible changes in metabolite concentrations. By analysing the complexes formed by specific enzymes, such as hexokinases and phosphofructokinases, we attributed this behaviour to the inability of the electrostatic forces to guarantee the interaction of these enzymes with the needed energy donors. In this regard, the agent-based approach shows one of its major capabilities: it reproduces the dynamics of local interactions among the molecules (modelled as autonomous agents) and “captures” the formation of complexes, even when they are partly-saturated enzymes.

Such a possibility allowed us to observe, in the system limited by short-range electrostatic interactions, a condition that might be detrimental for the cell anaerobic metabolism, which commits the production of energy (in the form of ATP) only to a fast-paced glycolytic process. At the current stage of our work, this consideration represents just a hypothesis: in real cells, glycolysis’ processes take place in times ranging from few seconds to hours^22,23,34,35^, making our one-second interval of simulation just a testbed to validate the capability of ABMs to support the study of the above-described forces in biological systems. However, if confirmed by further analyses, this result might suggest the non-feasibility of the *lock-and-key* model for enzymes in metabolic processes.

**Figure 4:**
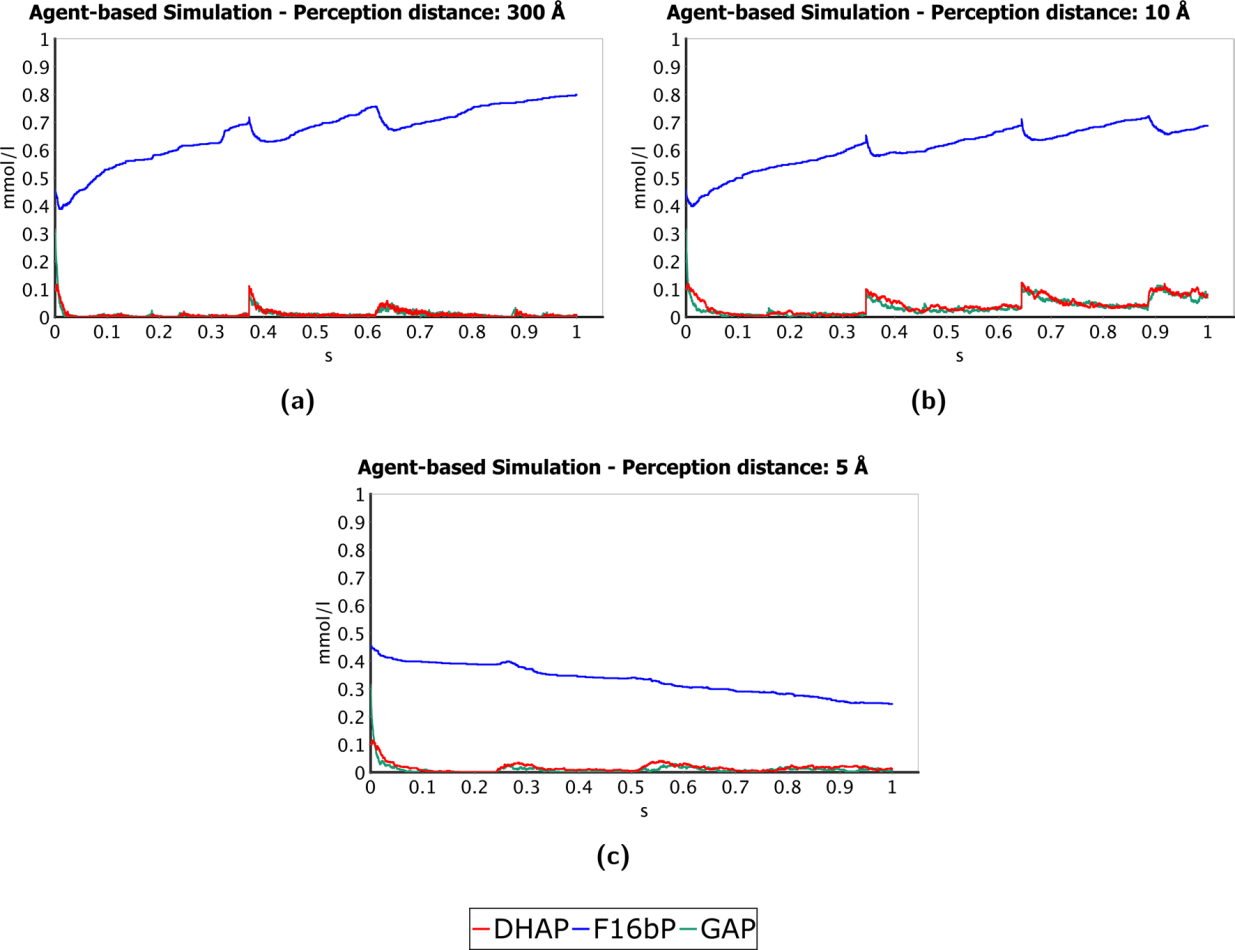
Synchronised oscillation-like fluctuations observed in fructose 1,6-bisphosphate (F16bP), dihydroxyacetone phosphate (DHAP) and glyceraldehyde 3-phosphate (GAP). The first metabolite is the product of the phosphorylation of fructose-6-phosphate, catalysed by phosphofructokinase, while the other two are generated by the subsequent reaction in the glycolytic pathway, carried out by fructose-bisphosphate aldolase. DHAP and GAP are also interconverted by the triosephosphate isomerase. In (**a,b**), that is, the plots of the simulations that take into account the electromagnetic forces (limited or not by the Debye screening), we can observe an oscillatory trend with a frequency of about 2.8 s$^{-1}$, synchronised in all the three curves. Conversely, in the simulation that considers just short-range electrostatic interactions, shown in plot (**c**), these oscillations are almost unnoticeable. The higher frequency measured experimentally in yeast’s glycolysis^35^ is of 30 s$^{-1}$; therefore, at the time scale of our simulations, these can be considered more as micro-oscillations, which give us a clue of the higher faithfulness to the real glycolytic process of the models whose interactions are not limited to just random encounters and chemical affinities.

Interestingly, even though enzymes regulation has not been modelled, the systems driven by electromagnetic forces (including those below the Debye screening length) produce oscillatory-like fluctuations in the concentrations of fructose 1,6-bisphosphtate, the main product of phosphofructokinase. Moreover, as shown in Fig. 4, these fluctuations are synchronised with the concentration changes of DHAP and GAP, the products of the subsequent reaction in the glycolytic pathway, especially due to its reversibility. Conversely, such behaviour is almost unnoticeable in the output of the simulation that allows only short-range van der Waals-like potentials (5 Å perception distance). Phosphofructokinase has a central role in the regulation of glycolysis and, pivoting around this enzyme, an oscillatory behaviour has been experimentally observed during the oxidation of glucose (even if at much lower frequencies)^35,36^. Considering the high level of abstraction of the current glycolysis ABM, this result might be considered another clue that, by not limiting molecular interactions to just shape complementarities and chemical affinities, we generated processes more faithful to those occurring in cellular glycolysis.

We could not reach such a conclusion if we based our analysis on a standard kinetic model, which derives the changes over time of the concentrations (often of metabolites alone) through rate and balance equations. As it lacks the capability to represent the granularity of a molecular system, this approach hardly grasps the fluctuations in the species amounts, generating several discrepancies with the results we gained through our agent-based simulations. Although differential equations best suit modelling the continuum or macroscale level^10^, such divergences might also be attributed to the possible inaccuracy through which kinetic parameters are essayed *in vitro*. Indeed, already in the early 2000s, Teusink et al. questioned that *in vitro* kinetics could be able to faithfully describe an *in vivo* behaviour^37^.

Furthermore, the standard kinetic modelling is based on the Michaelis-Menten formalism, which assumes diffusion-limited enzymatic reactions and a homogeneous environment, not considering the effects of molecular crowding on diffusion processes^38^. Other modelling approaches in Systems Biology (such as those based on Brownian dynamics or spatial partial differential equations) also struggled to deal with this problem due to the complexity of representing molecules 3D shape and diffusion at different scales^39^.

Molecular Dynamics simulations, which may not be affected by the limitations mentioned above, require a high number of physical parameters to be performed. Numerical simulations of this kind have been carried out to detect long-range interactions among biomolecules through the molecular diffusion behaviour^8^. In this case, simulating just one type of protein (the white egg Lysozyme) and one oppositely charged dye (the Alexa Fluor 488) required an *a priori* knowledge of several data (as mentioned in the “Results” section); applying the same approach to a complex pathway of many reactions would be significantly more difficult than performing *in silico* studies through agent-based simulations.

We think that the results provided in this article support the reliability of ABMs in capturing the essential features of a complex biological process and faithfully reproducing different aspects of its behaviour, even on the basis of few empirical data. This approach identifies in the long-range electrodynamic forces some of the fundamental “ingredients” necessary for glycolysis to operate in an efficient way.

With this manuscript, however, we just laid the groundwork for further *in silico* and experimental studies that would explore those aspects of metabolism dynamics overlooked at the current stage of our analysis. An optimised implementation of Orion would allow longer simulations that, complemented by experimental validation of the present results, might highlight if some of our outcomes could be biased by the abstraction level of the agent-based models. As a first step in this direction, we recently carried out a study where we experimentally demonstrate the activation of resonant electrodynamic intermolecular forces for bio-macromolecules with a long-range action; this result has been achieved by characterising clustering transitions induced by fluorescence correlation spectroscopy^40^.

Once the robustness of our agent-based approach is reinforced, we might pave the way for a better comprehension of those phenomena associated with cellular metabolism that are still not well understood. As an example, it can be applied in the study of the Warburg effect, which describes the preference of cancer cells for the anaerobic (and energetically inefficient) consumption of glucose through glycolysis, even in presence of a high oxygen concentration^41^. Recent studies have linked such a process to the effect of glycolytic oscillations^42^ and to the rate of glycolysis, increased to provide a selective advantage over metabolic competition in the tumour environment^43^. In this paper, we have shown how long-range electrodynamic forces may affect the rate and efficiency of glucose oxidation, as well as the oscillations in glycolysis intermediates; therefore, additional studies might enlighten us on their potential involvement in such an anomalous behaviour of tumour cells.

Similar results might also be reached by empowering the capabilities of the agent-based approach with methods from other disciplines. Among them, the topological data analysis, already used to better understand enzymatic reactions through ABMs^32^, may provide the current model with a many-body perspective. Shape calculus and hierarchical structures can be applied to represent molecular conformations and increase the accuracy of the interactions between cognate partners; these approaches would allow modelling the geometry of molecules shapes and collisions with a higher precision^44,45^. Moreover, we may better capture the collective synchronisation properties of a population of molecules behaving as coupled oscillators by using BOSL, the biological oscillators synchronisation logic^46^. Putting efforts in these directions might provide a new standpoint in our comprehension of molecular interactions and disclose aspects of biological systems that are still unexplored.

## Supplementary Information

Supplementary Information.

Supplementary Information.

## Data Availability

All data generated or analysed during this study are included in this published article (and its Supplementary Information files). The SBML of the Smallbone2013 - Iteration 18 model is accessible at http://identifiers.org/biomodels.db/MODEL1303260018; the modified version of this SBML, generated for running the time-course simulations with Copasi, is available at https://bit.ly/orion-simulator.

## Code availability

The simulations described in this manuscript have been performed with the agent-based simulator Orion 2.0.0. The simulator has been developed at the University of Camerino as a non-open and commercially-oriented software. For this reason, the source code is not openly released. To guarantee the methods reproducibility, a demo version of the simulator is accessible at https://bit.ly/orion-simulator.

## References

[1] McLachlan A (1964). A variational solution of the time-dependent Schrodinger equation. Mol. Phys..

[2] Stephen MJ (1964). First-order dispersion forces. J. Chem. Phys..

[3] Cherstvy AG, Kolomeisky AB, Kornyshev AA (2008). Protein-DNA interactions: Reaching and recognizing the targets. J. Phys. Chem. B.

[4] Painter PC, Mosher L, Rhoads C (1981). Low-frequency modes in the raman spectrum of DNA. Biopolymers.

[5] Fischer BM, Walther M, Jepsen PU (2002). Far-infrared vibrational modes of DNA components studied by terahertz time-domain spectroscopy. Phys. Med. Biol..

[6] Preto J, Floriani E, Nardecchia I, Ferrier P, Pettini M (2012). Experimental assessment of the contribution of electrodynamic interactions to long-distance recruitment of biomolecular partners: Theoretical basis. Phys. Rev. E.

[7] Preto J, Pettini M, Tuszynski JA (2015). Possible role of electrodynamic interactions in long-distance biomolecular recognition. Phys. Rev. E.

[8] Nardecchia I (2017). Detection of long-range electrostatic interactions between charged molecules by means of fluorescence correlation spectroscopy. Phys. Rev. E.

[9] Genesereth MR, Nilsson NJ (1987). Logical Foundations of Artificial Intelligence.

[10] Cannata, N., Corradini, F., Merelli, E. & Tesei, L. Agent-Based Models of Cellular Systems. In Reisfeld, B. & Mayeno, A. N. (eds.) *Computational Toxicology*, vol. 930 of *Methods in Molecular Biology*, 399–426, 10.1007/978-1-62703-059-5_18 (Humana Press, Totowa, NJ, 2013). 10.1007/978-1-62703-059-5_18 23086852

[11] Belenchia M (2021). Agent-based learning model for the obesity paradox in RCC. Front. Bioeng. Biotechnol..

[12] Norton K-A, Gong C, Jamalian S, Popel A (2019). Multiscale agent-based and hybrid modeling of the tumor immune microenvironment. Processes.

[13] Brazier F, Jonker C, Treur J (2000). Compositional design and reuse of a generic agent model. Appl Artif. Intell..

[14] Dastani, M., Arbab, F. & de Boer, F. Coordination and composition in multi-agent systems. In *Proceedings of the Fourth International Joint Conference on Autonomous Agents and Multiagent Systems - AAMAS ’05*, 439. 10.1145/1082473.1082540 (ACM Press, The Netherlands, 2005).

[15] Westerhoff, H. V. *et al.* From Silicon Cellsilicon cell to Silicon Humansilicon human. In Booß-Bavnbek, B., Klösgen, B., Larsen, J., Pociot, F. & Renström, E. (eds.) *BetaSys*, 437–458. 10.1007/978-1-4419-6956-9_19 (Springer New York, New York, NY, 2011).

[16] Smallbone K (2013). A model of yeast glycolysis based on a consistent kinetic characterisation of all its enzymes. FEBS Lett..

[17] Hucka M (2003). The systems biology markup language (SBML): A medium for representation and exchange of biochemical network models. Bioinformatics.

[18] World Wide Web Consortium (W3C). Extensible Markup Language (XML) 1.0 (Fifth Edition) (2013).

[19] The UniProt Consortium (2019). UniProt: A worldwide hub of protein knowledge. Nucleic Acids Res..

[20] Hastings J (2016). ChEBI in 2016: Improved services and an expanding collection of metabolites. Nucleic Acids Res..

[21] Guimarães PMR, Londesborough J (2008). The adenylate energy charge and specific fermentation rate of brewer’s yeasts fermenting high- and very high-gravity worts. Yeast.

[22] Laurent, M., Seydoux, F. J. & Dessen, P. Allosteric regulation of yeast phosphofructokinase. Correlation between equilibrium binding, spectroscopic and kinetic data. *J. Biol. Chem.* **254**, 7515–7520 (1979). 157354

[23] van den Brink J (2008). Dynamics of glycolytic regulation during adaptation of saccharomyces cerevisiae to fermentative metabolism. AEM.

[24] De la Fuente IM, Cortes JM (2012). Quantitative analysis of the effective functional structure in yeast glycolysis. PLoS ONE.

[25] Berg, J., Tymoczko, J. & Stryer, L. The Glycolytic Pathway Is Tightly Controlled. In *Biochemistry* (New York: W H Freeman, 2002), 5th edition edn.

[26] Jurica MS (1998). The allosteric regulation of pyruvate kinase by Fructose-1,6-Bisphosphate. Structure.

[27] Angeletti, M. *et al.* Spatial behavioral modeling and simulation of metabolic pathways with Orion. In *IV Bioinformatics ITalian Society Meeting (BITS 2007)*, 70 (Napoli, Italy, 2006).

[28] Cannata, N., Corradini, F. & Merelli, E. Multiagent modelling and simulation of carbohydrate oxidation in cell. *Int J Model. Identif. Control* **3**. 10.1504/IJMIC.2008.018191 (2008).

[29] Richards FM (1977). Areas, volumes, packing and protein structure. Annu. Rev. Biophys. Bioeng..

[30] Zamyatnin A (1972). Protein volume in solution. Prog. Biophys. Mol. Biol..

[31] Harpaz, Y., Gerstein, M. & Chothia, C. Volume changes on protein folding. *Structure* **2**, 641–649. 10.1016/S0969-2126(00)00065-4 (1994). 10.1016/s0969-2126(00)00065-4 7922041

[32] Piangerelli M, Maestri S, Merelli E (2020). Visualising 2-Simplex formation in metabolic reactions. J. Mol. Graph. Model..

[33] Hoops S (2006). COPASI-a COmplex PAthway SImulator. Bioinformatics.

[34] Nielsen K, Sørensen P, Hynne F, Busse H-G (1998). Sustained oscillations in glycolysis: An experimental and theoretical study of chaotic and complex periodic behavior and of quenching of simple oscillations. Biophys. Chem..

[35] Richard P (2003). The rhythm of yeast. FEMS Microbiol. Rev..

[36] Wolf J (2000). Transduction of intracellular and intercellular dynamics in yeast glycolytic oscillations. Biophys. J ..

[37] Teusink B (2000). Can yeast glycolysis be understood in terms of in vitro kinetics of the constituent enzymes? Testing biochemistry: Do we understand yeast glycolysis?. Eur. J. Biochem..

[38] Berry H (2002). Monte Carlo simulations of enzyme reactions in two dimensions: Fractal kinetics and spatial segregation. Biophys. J ..

[39] Takahashi K, Arjunan SNV, Tomita M (2005). Space in systems biology of signaling pathways:Towards intracellular molecular crowding in silico. FEBS Lett..

[40] Lechelon, M. *et al.* Experimental evidence for long-distance electrodynamic intermolecular forces (2021). Working paper or preprint, HAL. https://hal.archives-ouvertes.fr/hal-03259009. 10.1126/sciadv.abl5855 PMC8849397 35171677

[41] Warburg O (1925). The metabolism of carcinoma cells. J. Cancer Res..

[42] Rietman EA (2013). An integrated multidisciplinary model describing initiation of cancer and the Warburg hypothesis. Theor. Biol. Med. Model.

[43] Liberti MV, Locasale JW (2016). The Warburg effect: How does it benefit cancer cells?. Trends Biochem. Sci..

[44] Buti F, Cacciagrano D, Corradini F, Merelli E, Tesei L (2010). BioShape: A spatial shape-based scale-independent simulation environment for biological systems. Proc. Comput. Sci..

[45] Quadrini, M., Daberdaku, S. & Ferrari, C. Hierarchical Representation and Graph Convolutional Networks for the Prediction of Protein–Protein Interaction Sites. In Nicosia, G. *et al.* (eds.) *Machine Learning, Optimization, and Data Science*, vol. 12566, 409–420. 10.1007/978-3-030-64580-9_34 (Springer International Publishing, Cham, 2020).

[46] Bartocci E, Corradini F, Merelli E, Tesei L (2010). Detecting synchronisation of biological oscillators by model checking. Theoret. Comput. Sci..

