# Supplementary material for: Agent-based models for detecting the driving forces of biomolecular interactions
Source: Sci Rep. 2022 Feb 3;12:1878. doi: 10.1038/s41598-021-04205-8 (PMC8814177; doi:10.1038/s41598-021-04205-8)
Supplement: Supplementary file 1 — Supplementary Information. [file 41598_2021_4205_MOESM1_ESM.pdf]

Supplementary Information to *Agent-based models for detecting the driving forces of biomolecular interactions*

Stefano Maestri[1,2], **Emanuela Merelli**[1,*], **and Marco Pettini**[2]

[1]School of Science and Technology, University of Camerino, Camerino, 62032, Italy
[2]Aix-Marseille Univ, Université de Toulon, CNRS, Centre de Physique Théorique, 13288, Marseille, France
[*]emanuela.merelli@unicam.it

This document is intended to provide additional details on the models and results discussed in the paper. Since we simulated the glycolysis of baker's yeasts (Saccharomyces cerevisiae), we firstly introduce, in Section 1, some basic knowledge on glucose oxidation in living cells. In Section 2, we then go into the details of the modelling and simulation methods; this section represents a complement to the description provided in the article, aimed at clarifying those aspects of our models that the latter does not fully cover. Finally, in Section 3, we analyse the plots of the concentration changes over time relevant to better understand our results. In Appendix A1, we provide the XML file generated, as input for the simulations, from the SBML representation of the Smallbone2013 kinetic model.

Contents

1 Introduction to Glycolysis **2**
2 Agent-based Approach **2**
2.1 Enzymatic Reaction Automaton . . . 2
2.2 Defining the Input for the Simulation . . . 4
2.3 Simulation Output and Visualisation . . . 10
2.4 Additional Information on the Perception Spheres - Interaction Probabilities . . . 11
3 Plots of the Concentration Changes over Time **14**
3.1 Metabolite Concentration Changes . . . 14
3.2 Comparison of Relevant Complexes Formation . . . 20
References **26**
A1 XML input file **27**

List of Tables

S1 Modelled molecular species - initial concentrations and sphere radii . . . 11
S2 Modelled reactions and related turnover numbers . . . 12

List of Plots

S4 Concentration changes over time of ADP, ATP, BPG, DHAP, F16bP, F6P . . . 16
S5 Concentration changes over time of G1P, G3P, G6P, GAP, GLC, GLY . . . 17
S6 Concentration changes over time of NAD, NADH, P2G, P3G, PEP, PYR . . . 18
S7 Concentration changes over time of T6P, TRH, UDG, UDP, UTP . . . 19
S8 Phosphorylation of GLC to G6P performed by HXK1 and HXK2 isoenzymes . . . 21
S9 Phosphorylation of GLC to G6P performed by GLK1 . . . 22
S10 Phosphorylation of F6P to F16bP performed by PFK1 and PFK2 isoenzymes . . . 23
S11 Conversion of DHAP to G3P catalysed by GPD1 . . . 24
S12 Conversion of DHAP to G3P catalysed by GPD2 . . . 25

1 Introduction to Glycolysis

This section aims to give an overview of the reactions occurring in the glycolytic pathway; the description is fairly general and based on long-established knowledge on glucose oxidation[1]. The reader already familiar with these concepts can jump directly to Section 2, where we provide additional details on the modelling and simulation methods described in the article.

Glycolysis is the process that degrades, through a series of enzyme-catalysed reactions, a molecule of `glucose` to yield two molecules of `pyruvate` and store some of the released free energy in the form of `ATP` and `NADH`. When glucose degradation happens in the absence of oxygen (anaerobic conditions), it is called fermentation.

The enzymes involved in the glycolysis of all eukaryotic cells are similar in their structures and functions; they only differ in the regulatory processes that determine the fate of pyruvate. The sequential reactions of the glycolytic pathway are usually schematised in *ten steps*. In what follows, we describe the most relevant of these steps and provide the name and the acronym of the related molecular species by which we refer to them in this supplementary document and in the article.

The initial five steps constitute the *preparatory phase*;

- *first step*: `glucose` (`GLC`) is phosphorylated to form `glucose 6-phosphate` (`G6P`)
- *second step*: `G6P` is converted to `fructose 6-phosphate` (`F6P`)
- *third step*: `F6P` is phosphorylated to `fructose 1,6-bisphosphate` (`F16bP`)

  (for both the phosphorylations, `ATP` is the phosphoryl group donor)
- *fourth step*: `F16bP` is split into `dihydroxyacetone phosphate` (`DHAP`) and `glyceraldehyde 3-phosphate` (`GAP`)
- *fifth step*: `DHAP` is converted to a second molecule of `GAP`

The energy gain occurs in the *payoff phase*, composing the remaining five steps of glycolysis. In the *sixth step*, each molecule of `GAP` is oxidised and phosphorylated to form `1,3-bisphosphoglycerate` (`BPG`). Energy is then released by converting, *from the seventh to the tenth step*, two molecules of `BPG` into two molecules of `pyruvate` (`PYR`). Much of this energy is conserved, by the phosphorylation of four `ADP` molecules, into an equal number of `ATPs`. Since two molecules of `ATP` are used in the preparatory phase, the net output is two `ATPs` for each molecule of `glucose` degraded. During the payoff phase, energy is also stored by forming two molecules of `NADH` for each molecule of `glucose`.

In yeasts, `pyruvate` is further converted, under anaerobic conditions, into `ethanol` (`EtOH`) and CO_2, a process called *ethanol (alcohol) fermentation*.

Among the other carbohydrates involved in glycolysis, the only one we take into account in our models is `glucose 1-phosphate` (`G1P`), which is converted to `G6P` during the preparatory phase.

Alongside the main steps described above, the breakdown of `glucose` can also enter one of the glycolysis branches, which leads to the formation of end products such as `trehalose` (`TRH`), `glycerol` (`GLY`) and `succinate` (`SUC`).

A schematic representation of the steps and branches considered in our work is provided in Fig. 2 of the paper.

2 Modelling and Simulating the Glycolytic Pathway

2.1 Enzymatic Reaction Automaton

In this section, we describe and formalise the *reaction automaton* introduced in the Methods section of the paper; this model allows simulating biochemical reactions as resulting from the local interactions among enzymes and cognate metabolites.

According to the Michaelis-Menten model of enzyme kinetics, an enzymatic reaction can be represented as:

$$E + S \leftrightharpoons ES \rightarrow E + P \tag{S1}$$

Where E is an enzyme, S its substrate and P the product of the reaction catalysed by E; assuming the *steady-state approximation*, we can consider ES as constant[2,3].

However, by taking into account local interactions in the dynamics of a biochemical reaction, we can abstract the following molecular entities[4]:

- *Free enzyme*, seeking a substrate to interact with.
- *Dual-complex*, formed when an enzyme binds a cognate metabolite but needs another molecule (such as an energy donor) to saturate; it is unstable because the second metabolite is necessary to generate the final products of the reaction.
- *Saturated enzyme*, corresponding to the final complex of the reaction; it is formed by an enzyme linked to one or two metabolites, stably for a time interval obtained as the inverse of the turnover number (k_{cat}) of the reaction. The k_{cat} represents the number of molecules converted by an enzyme in the time unit; therefore, its reciprocal provides the interval after which the reaction products are released in the environment, and the enzyme returns free.

An enzymatic reaction cycles through these states; we model such a pattern by constructing an *automaton* based on the molecular entities described above. To reproduce the local interactions properly, we represent each type of molecule as an autonomous entity; as discussed in the paper, this entity corresponds, in our model of glycolysis, to an *agent*, a system *having the capability of perceiving and interacting with its environment.*

We provide a formal definition of the automaton through Milner's CCS (Calculus of Communicating Systems). This process algebra consists of a collection of constructors for building a new process description from existing ones by representing them as systems that exhibit behaviour and interact via synchronised communication[5,6]. A process can be viewed as a black box with a name and a set of communication channels. An input action on the channel a is indicated using the labels a, while an output on the same channel is represented by $\overline{a}$. For the aim of this discussion, we consider the following two CCS constructors. Let $\mathcal{P}$, $\mathcal{Q}$ be processes,

- *Action prefixing:* if a is an action, $a.\mathcal{P}$ is a process that begins by performing the action a and behaves like $\mathcal{P}$ thereafter.
- *Choice operator:* $\mathcal{P}+\mathcal{Q}$ is a process that may behave like $\mathcal{P}$ or $\mathcal{Q}$.

Using the CCS process algebra, we can define an enzymatic reaction as a process $\mathcal{R}$ such that:

$$
\begin{aligned}
&\mathcal{R} \stackrel{\text{def}}{=} e.E_{m1} + e.E_{m2};\\
&E_{m1} \stackrel{\text{def}}{=} m1.DC1 + m1.ES;\\
&E_{m2} \stackrel{\text{def}}{=} m2.DC2;\\
&DC1 \stackrel{\text{def}}{=} m2.DC1_{m2};\\
&DC2 \stackrel{\text{def}}{=} m1.DC2_{m1};\\
&DC1_{m2} \stackrel{\text{def}}{=} m2.ES;\\
&DC2_{m1} \stackrel{\text{def}}{=} m1.ES;\\
&ES \stackrel{\text{def}}{=} \overline{pe}.\mathcal{R};
\end{aligned}
$$

Where:

- e is a free enzyme;
- $m1$ is the primary substrate of the enzyme;
- $m2$ is a secondary substrate of the enzyme, such as an energy donor;
- pe generalises the products of the reaction (one or more) and the enzyme that returns free;
- E_{m1} and E_{m2} are the processes that represent the enzyme perceiving a cognate metabolite;
- $DC1$ and $DC2$ correspond to the dual-complexes of the enzyme with m1 and m2, respectively;
- $DC1_{m2}$ and $DC2_{m1}$ are the processes through which the dual complexes perceive the metabolite needed to saturate the enzyme;
- ES represents the saturated enzyme.

To better highlight all the processes and actions characterising the reaction automaton, in Fig. S1, we provide the Labeled Transition System (LTS)[7] related to the previous algebraic specification.

Since the simulator represents the molecules as spheres, we are able to implement this model by allowing the formation of larger spheres as the result of the interaction between two cognate molecules. The volume of the sphere corresponding to a molecular complex is calculated from the sum of the originating molecules' weights on the basis of Equation S2 described in the following Section 2.2. Figure S2 provides a schematic representation of the automaton for the case in which the enzyme interacts with two metabolites.

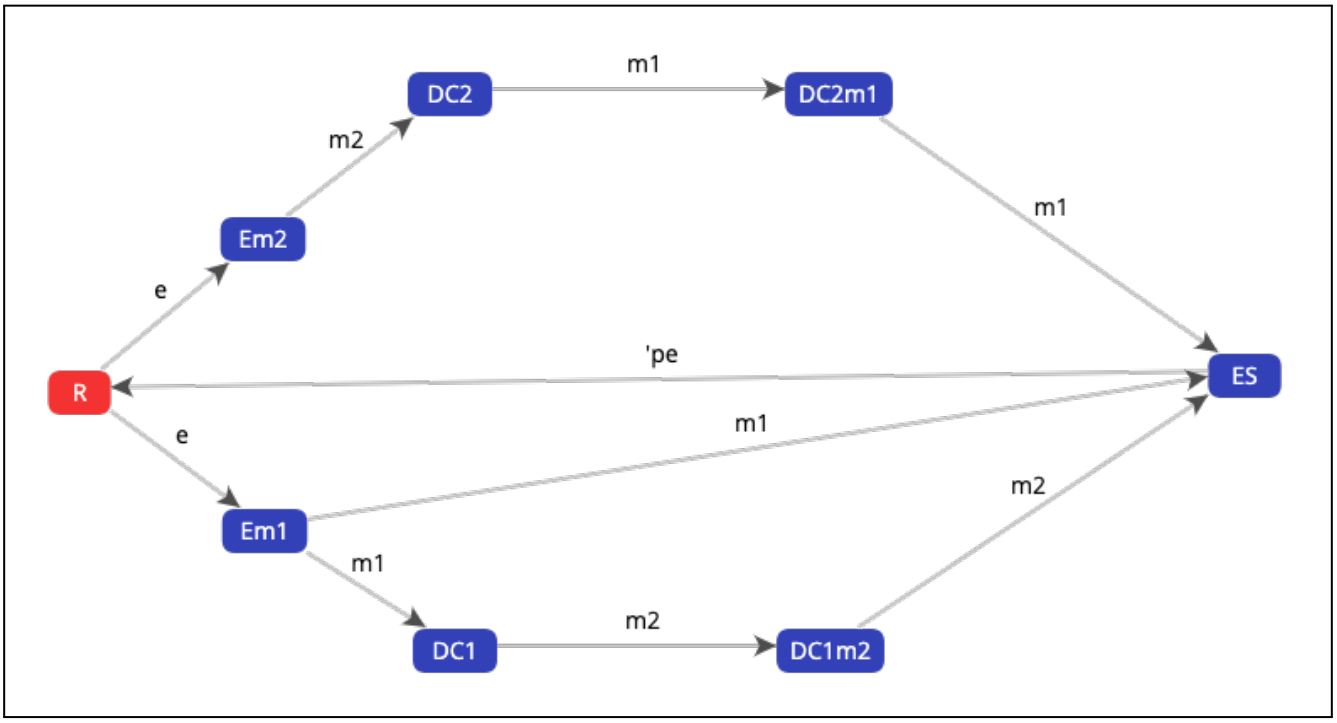

Figure S1. Labeled Transition System (LTS) of the automaton representing an enzymatic reaction in our agent-based model. It consists of a set of processes, a set of actions and a transition relation $\rightarrow$ such that, if a process P can perform an action a and become a process P', we write $P \xrightarrow{a} P'$[5]. The shown LTS has been generated, from the algebraic definition provided in Sction 2.1, through the web-based tool CAAL[8]. The names of the processes are transliterations of the names provided in the CCS specification.

2.2 Defining the Input for the Simulation

The input of the agent-based simulator described in the manuscript is an SBML (Systems Biology Markup Language) file filled with experimental data[9–14]. It contains information about the molecules involved in the metabolic pathway and their initial concentrations; data related to the reactions carried out are also taken from this SBML file. For the study described in the manuscript, we chose as the source of our simulations the Smallbone2013 - Iteration 18 model[15], provided in the SBML format (`http://identifiers.org/biomodels.db/MODEL1303260018`).

A dedicated module of the simulator converts the SBML model to an Extensible Markup Language (XML) file specifically formatted to be interpreted by the simulator itself, but also to be human-readable[16]. Therefore, its main function is to translate the kinetic representation of the metabolic reactions into our agent-based model. To do this, for every reaction–in the SBML file–we want to model, it gets the reactants and products and generates XML code for each of its interactions, based on the algebraic definition of the automaton provided in Section 2.1. It also associates to the reaction its k_{cat} (as described in the previous subsection) and the K_m values of all its enzyme-substrate interactions. The K_m measures the affinity of an enzyme for a specific substrate; it is needed since an enzyme can form a complex with an encountered metabolite randomly or based on a priority list constructed over the k_{cat}/K_m ratio (specificity constant). This possibility can be established in the initial setup of the simulation. For the study proposed in the article, we set the molecular interactions to be completely random, without predetermining any priority on the metabolites perceived by an enzyme.

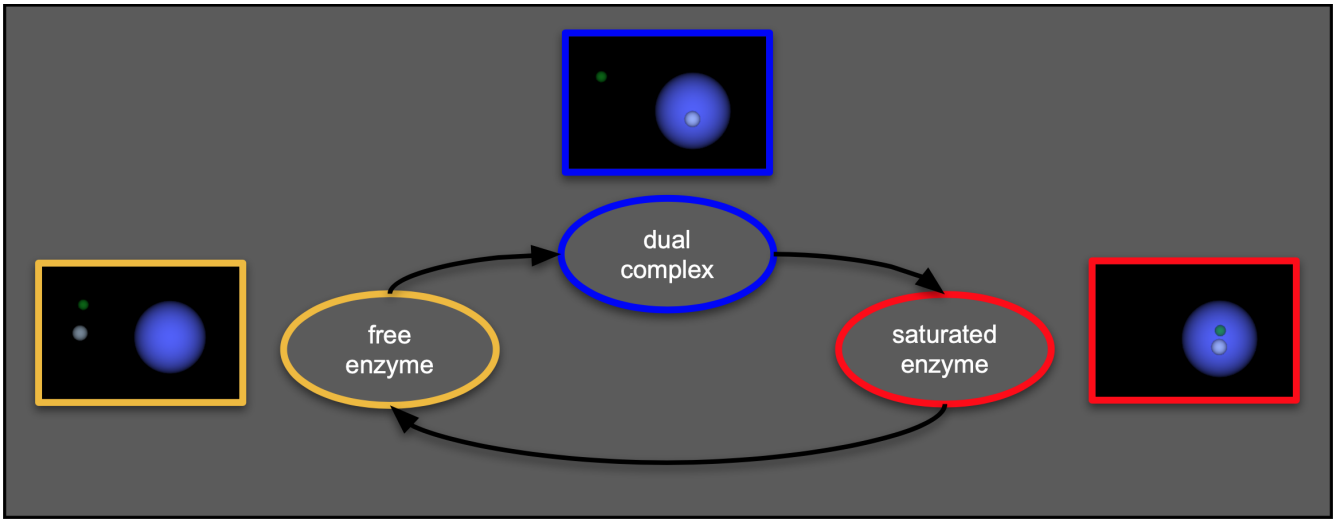

Figure S2. The three states of the enzymatic reaction automaton in which the enzyme interacts with two metabolites[4]. Each state has been associated with a representation of the related molecular entities in the agent-based model. For better showing the molecules involved in the formation of a molecular complex, we choose to draw dual-complexes and saturated enzymes as paired spheres; however, in the actual implementation, each of them is represented by a single sphere whose volume is obtained, from the sum of the weights of the generating molecules, through Equation S2.

SBML and XML (from which the first is derived) are markup languages that define rules for storing data in a formatted document so as to comply with both human and machine readability[9,16]. They are structured as element trees: starting from a root, each element of the tree can have one or more child elements. Every element is delimited by an opening tag, in which the element name is enclosed between angle brackets (`<` and `>`), e.g. `<element_name>`, and a closing tag, similar to the opening tag but with the element name preceded by a slash symbol (`/`), e.g. `</element_name>`. It can also have one or more attributes, placed inside the opening tag in the form: `attribute_name = "attribute_value"`.

In what follows, we provide a simplified conversion of a kinetic model, in SBML format, to the XML input of our agent-based simulator; we consider a generalised reaction that is catalysed by an enzyme `E`, with two substrate metabolites (`M1` and `M2`) and two products (`P1` and `P2`).

Starting from the SMBL source:

```
<reaction metaid="meta_E" sboTerm="SBO:0000176" id="E" name="reaction_name">
    <annotation>
      <rdf:RDF xmlns:rdf="http://www.w3.org/1999/02/22-rdf-syntax-ns#"
      xmlns:bqmodel="http://biomodels.net/model-qualifiers/"
      xmlns:bqbiol="http://biomodels.net/biology-qualifiers/">
        <rdf:Description rdf:about="#meta_E">
          <bqbiol:is>
            <rdf:Bag>
              <rdf:li rdf:resource="resource_url"/>
            </rdf:Bag>
          </bqbiol:is>
          <bqbiol:isVersionOf>
            <rdf:Bag>
              <rdf:li rdf:resource="identifier_url"/>
              <rdf:li rdf:resource="identifier_url"/>
            </rdf:Bag>
          </bqbiol:isVersionOf>
        </rdf:Description>
```

```
  </rdf:RDF>
 </annotation>
 <listOfReactants>
   <speciesReference metaid="metaid_value" species="M1"/>
    <speciesReference metaid="metaid_value" species="M2"/>
 </listOfReactants>
 <listOfProducts>
   <speciesReference metaid="metaid_value" species="P1"/>
   <speciesReference metaid="metaid_value" species="P2"/>
 </listOfProducts>
 <listOfModifiers>
   <modifierSpeciesReference metaid="metaid_value" species="E"/>
   <modifierSpeciesReference species="P1"/>
   <modifierSpeciesReference species="P2"/>
   <modifierSpeciesReference species="E"/>
 </listOfModifiers>
 <listOfParameters>
    <parameter metaid="metaid_value" id="kcat" value="kcat_value" units="per_second"/>
    <parameter metaid="metaid_value" id="Km1" value="Km1_value"  units="mM"/>
    <parameter metaid="metaid_value" id="Km2" value="Km2_value"  units="mM"/>
 </listOfParameters>
```

the conversion module generates the corresponding XML code:

```
    <reaction>
     <interaction>
       <reactants>
         <reactant id="E"/>
         <reactant id="M1"/>
       </reactants>
       <products>
         <product id="E+M1"/>
       </products>
       <Km unit="mM">Km1_value</Km>
     </interaction>
     <interaction>
       <reactants>
         <reactant id="E"/>
         <reactant id="M2"/>
       </reactants>
       <products>
         <product id="E+M2"/>
       </products>
       <Km unit="mM">Km2_value</Km>
     </interaction>
     <interaction>
       <reactants>
         <reactant id="E+M1"/>
         <reactant id="M2"/>
       </reactants>
       <products>
         <product id="E+M1+M2"/>
       </products>
       <Km unit="mM">Km2_value</Km>
     </interaction>
     <interaction>
```

```
      <reactants>
        <reactant id="E+M2"/>
        <reactant id="M1"/>
      </reactants>
      <products>
        <product id="E+M1+M2"/>
      </products>
      <Km unit="mM">Km1_value</Km>
    </interaction>
    <interaction>
      <reactants>
        <reactant id="E+M1+M2"/>
      </reactants>
      <products>
        <product id="P1"/>
        <product id="P2/>
        <product id="E"/>
      </products>
      <Km unit="mM">0.0</Km>
    </interaction>
    <kcat unit="per_second">kcat_value</kcat>
  </reaction>
```

Where `E+M1` and `E+M2` are dual complexes (the processes $DC1$ and $DC2$ of the algebraic model), while `E+M1+M2` represents the saturated enzyme. The K_m of the last interaction is always 0 because this process models the release of the reaction products after the time interval given by the k_{cat} reciprocal.

The conversion module also retrieves from online databases, specifically ChEBI[17] and UniProt[18], the molecular weights (MW), needed for the simulation but missing in the SBML model. These values are required to obtain the volumes of the spheres that represent the molecules of the simulation; they are calculated through the average value of the molar specific volume of a protein in solution (approximately 0.73 cm^3/g) by assuming the following equation[19–22]:

$$V(\text{Å}^3) = \frac{0.73\, cm^3/g \times 10^{24}\ \text{Å}^3/cm^3 \times \text{MW}\ g/mole}{6.02 \times 10^{23}\ \ molecules/mole} \tag{S2}$$

According to the data retrievable in the BioNumbers database, the average values of the molecular radii are about 20 Å (angstroms) for enzymes and 5 Å for metabolites[23]. By looking at Table S1, the radii generated from the volumes calculated through Equation S2 are in agreement with those experimental results.

The XML file is structured in four main parts:

1. unit definitions;
2. list of metabolites and enzymes in the modelled cytoplasm portion at the beginning of the simulation (along with the list of the complexes that may form during the metabolic process);
3. list of all the reactions that may occur in the metabolic pathway;
4. ambient and interaction properties of the simulation.

The first three parts are the children of the "`pathway`" element because they, indeed, set the properties of the modelled metabolic pathway.

The *unit definitions* provide a list of all the derived units adopted in the model and specify how they are obtained from the SI (Système International) base units. For example, the definition of the unit *millimolar* (mM or mmol/l) is given by the following XML code:

```
<unitDefinition id="mM">
   <unit exponent="1" kind="mole" multiplier="1" scale="-3"/>
   <unit exponent="-1" kind="litre" multiplier="1" scale="0"/>
</unitDefinition>
```

The *list of molecules* part reports all the metabolites, enzymes and complexes involved in the modelled metabolic process. It also stores the initial concentrations and the molecular weights (which the simulator retrieves from online databases, as explained before). For molecular complexes, the initial concentrations are always zero, while their molecular weights are calculated as the sum of the weights of the molecules that compose each of them.

As an example of XML code for a metabolite, `glucose` (`GLC`) is defined as follows:

```
<!--metabolite - ChEBI name: D-glucopyranose; resource: http://identifiers.org/chebi/CHEBI:4167 -->
<molecule compartment="cell" id="GLC" name="glucose" type="Metabolite">
   <molecularWeight unit="dalton">180.06</molecularWeight>
   <initialConcentration unit="mM">0.6280001793382419</initialConcentration>
</molecule>
```

The `compartment` is reported as an attribute for allowing the possibility of modelling membrane transport (not implemented in the simulations described in the paper); `name` and `id` are taken from the SBML model, but the database name is also indicated in the comment (the first line, delimited by `<!--` and `-->`), along with the link to the online database record.

A similar approach is used to define enzymes and complexes. To improve the file's readability, the simulator groups each enzyme definition with those of the complexes that such enzyme can form. For example, considering the isoenzyme `hexokinase-1` (`HXK1`), since it can interact with `glucose` and `ATP`, this enzyme and the related complexes are listed in the following way:

```
<!--Reaction: Hexokinase [HXK1]-->
<!--enzyme - UniProt name: Hexokinase-1; resource: https://www.uniprot.org/uniprot/P04806 -->
<molecule compartment="cell" id="HXK1" name="HXK1" type="Enzyme">
   <molecularWeight unit="dalton">53738.0</molecularWeight>
   <initialConcentration unit="mM">0.0167807457149784</initialConcentration>
</molecule>
<molecule compartment="cell" id="HXK1+GLC" name="HXK1+glucose" type="Complex">
   <molecularWeight unit="dalton">53918.06</molecularWeight>
   <initialConcentration unit="mM">0</initialConcentration>
</molecule>
<molecule compartment="cell" id="HXK1+ATP" name="HXK1+ATP" type="Complex">
   <molecularWeight unit="dalton">54245.0</molecularWeight>
   <initialConcentration unit="mM">0</initialConcentration>
</molecule>
<molecule compartment="cell" id="HXK1+GLC+ATP" name="HXK1+glucose+ATP" type="Complex">
   <molecularWeight unit="dalton">54425.06</molecularWeight>
   <initialConcentration unit="mM">0</initialConcentration>
</molecule>
```

The *list of reactions* is in the form already described in this section; for completeness, we show, instead of just a generalisation, the reaction catalysed by `HXK1`, that is:

$$\texttt{GLC} + \texttt{ATP} \xrightarrow{\texttt{HXK1}} \texttt{G6P} + \texttt{ADP}$$

The XML code generated for this reaction is the following:

```
<!--Hexokinase [HXK1]: irreversible reaction - forward direction-->
      <reaction>
        <interaction>
          <reactants>
            <reactant id="HXK1"/>
            <reactant id="GLC"/>
          </reactants>
          <products>
```

```
        <product id="HXK1+GLC"/>
      </products>
      <Km unit="mM">0.15</Km>
    </interaction>
    <interaction>
      <reactants>
        <reactant id="HXK1"/>
        <reactant id="ATP"/>
      </reactants>
      <products>
        <product id="HXK1+ATP"/>
      </products>
      <Km unit="mM">0.293</Km>
    </interaction>
    <interaction>
      <reactants>
        <reactant id="HXK1+GLC"/>
        <reactant id="ATP"/>
      </reactants>
      <products>
        <product id="HXK1+GLC+ATP"/>
      </products>
      <Km unit="mM">0.293</Km>
    </interaction>
    <interaction>
      <reactants>
        <reactant id="HXK1+ATP"/>
        <reactant id="GLC"/>
      </reactants>
      <products>
        <product id="HXK1+GLC+ATP"/>
      </products>
      <Km unit="mM">0.15</Km>
    </interaction>
    <interaction>
      <reactants>
        <reactant id="HXK1+GLC+ATP"/>
      </reactants>
      <products>
        <product id="G6P"/>
        <product id="ADP"/>
        <product id="HXK1"/>
      </products>
      <Km unit="mM">0.0</Km>
    </interaction>
    <kcat unit="per_second">10.2</kcat>
  </reaction>
```

The fourth part of the XML file is placed outside the "`pathway`" element; it specifies the physical parameters of the modelled cytoplasm portion that are needed to reproduce Brownian motion (see the Methods section of the paper), as well as the properties of molecular interactions. Specifically, it lists:

- volume of the cytoplasm portion (in attoliters);
- viscosity of the environment (in pascal-seconds);

- temperature (in Kelvin degrees);
- perception distance of the active molecules (in angstroms);
- the possibility or not for enzymes to prioritise interactions on the basis of the specificity constant (k_{cat}/K_m ratio, as previously explained in this section).

Except for the perception distance, which we set to 5, 10 or 300 Å (as widely discussed in this document and in the article), we configure the other simulation parameters as in the following XML code:

```
<!--Ambient settings-->
  <ambientSettings>
    <volumeOfSimulation unit="attolitre">1</volumeOfSimulation>
    <viscosity unit="pascal_second">0.0011</viscosity>
    <temperature unit="kelvin">298.15</temperature>
  </ambientSettings>
<!--Interaction settings-->
  <interactionSettings>
    <perceptionDistance unit="angstrom">300</perceptionDistance>
    <priorityBySpecificity>false</priorityBySpecificity>
  </interactionSettings>
```

Appendix A1 of this supplementary document reports the actual XML file generated for the simulations proposed in the manuscript.

In the article, we describe and explain the changes we made to the Smallbone2013 model by converting it to our agent-based model of glycolysis. The result of all these modifications is the set of the reactions actually simulated, which are listed in Table S2 of this supplementary document and schematised in Fig. 2 of the article.

In addition to the imported reactions types, we also made some adjustments to the molecular species initial concentrations provided in the original SBML model; this is needed to comply with the computational demand of the simulations. In fact, agent-based simulations have a fairly light computational load, but reproducing a metabolic pathway involves thousands of molecules, and thus as many agents running concurrently. The resulting resources demand conditioned the molecular concentrations we were able to simulate. More precisely, we scaled the concentrations provided by the Smallbone2013 model to values less than 1 mmol/l. In Table S1, we report the initial concentrations of all the simulated species. The total number of molecules (enzymes and metabolites) in the environment at the beginning of the simulation is 6955.

2.3 Simulation Output and Visualisation

The output of the simulator is a set of CSV (Comma-Separated Values) files reporting the type and number of molecules contained in the simulated environment, along with their position, at each instant of simulation. More precisely, the simulator generates three types of CSV files:

- A *standard output*, reporting type (metabolite, enzyme or complex) and concentration (in mmol/l) of every molecular species in the modelled cytoplasm portion at each instant of the simulation. Such files are used to generate the plots provided in the article and in Section 3 of this supplementary document.
- A *"verbose" output*, which, differently from the previous one, lists each molecule in the environment at every instant of the simulation, along with its radius (in picometres) and the coordinates of its position in the three-dimensional space. This file is specifically designed to generate a 3D representation of the simulated environment through a dedicated interface of the simulator; it is also necessary to restart an interrupted simulation.
- An *"interactors" output*, containing, for each simulation time step, the enzymes perceiving one or more metabolites, and a list of these molecules; such a file is needed to highlight enzymes' perception in the 3D interface. The generation of this file is optional and can be excluded to reduce the computational demand of the simulation.

In Fig. S3 we provide a screenshot of the simulated environment after 5.9 ms of simulation and the related plots of the concentration changes generated through the simulator interface.

Metabolites			Enzymes		
Name	Initial Conc. (mmol/l)	Sphere Radius (Å)	Name	Initial Conc. (mmol/l)	Sphere Radius (Å)
ADP	0.129	4.98	CDC19	0.205	25.07
AMP	0.44	4.65	ENO1	0.686	23.83
ATP	0.429	5.28	ENO2	0.197	23.85
BPG	0.007	4.26	FBA1	0.134	22.54
DHAP	0.116	3.67	GLK1	0.045	25.2
F16bP	0.458	4.62	GPD1	0.068	23.14
F26bP	0.030	4.62	GPD2	0.008	24.26
F6P	0.235	4.22	GPM1	0.730	19.98
G1P	0.539	4.22	HOR2	0.055	20.03
G3P	0.274	3.68	HXK1	0.017	24.95
G6P	0.772	4.22	HXK2	0.061	24.98
GAP	0.316	3.67	PFK1	0.047	31.48
GLC	0.628	3.74	PFK2	0.039	31.15
GLY	0.150	2.99	PGI1	0.138	26.07
NAD	0.150	5.77	PGK1	0.258	23.47
NADH	0.087	5.78	PGM1	0.033	26.32
P2G	0.068	3.78	PGM2	0.013	26.32
P3G	0.470	3.78	PYK2	0.061	25.17
PEP	0.610	3.65	RHR2	0.051	20.07
PYR	0.211	2.93	TDH1	0.351	21.78
T6P	0.020	4.96	TDH3	0.420	21.78
TRH	0.015	4.63	TPI1	0.294	19.79
UDG	0.467	5.47	TPS1	0.034	25.32
UDP	0.282	4.89	TPS2	0.027	30.99
UTP	0.649	5.18	UGP1	0.062	25.29

Table S1. Initial concentrations and sphere radii of the molecular species simulated in our study. The original amounts provided by the Smallbone2013 - Iteration 18 model have been scaled (to values less than 1 mmol/l) to fit the computational demand of the simulations. Each radius is obtained from the volume calculated through Equation S2.

2.4 Additional Information on the Perception Spheres - Interaction Probabilities

As detailed in the article, interactions between active molecules (free enzymes and dual-complexes) and metabolites are modelled through perception capabilities specifically designed. Their core property lies in the definition of a *perception sphere* that surrounds each active molecule. The perception radius–the radius of the perception sphere–can be set according to needs; therefore, we can test various perception lengths and the related molecular behaviours (as schematised in Fig. 1b of the article).

Each perception radius is obtained by summing the radius of the enzyme to the *perception distance* at which we want the enzyme to be able to find a cognate metabolite; the perception distance extends beyond the surface of the sphere representing the enzyme. As the distance of the metabolite from the enzyme increases, the intensity of the forces acting on a metabolite diminishes; for this reason, each perception sphere is characterised by different interaction probabilities, depending on its size.

Since we aim to compare the effects of long- and short-distance interactions, we focus the study proposed in the paper on three perception distances: 5, 10 and 300 Å.

- A perception distance of 5 Å sets the space affected only by van der Waals-like potentials; when a metabolite enters the related sphere (of a cognate enzyme), there is a probability $p = 1$ that the interaction will happen.

- A 10 Å perception distance models the effect of the Debye screening. In this case, the probability of the interaction is still $p = 1$ when the metabolite is, at most, 5 Å far from the enzyme sphere; it reduces to $p = 1/2$ when the metabolite is detected at a distance d such that $5 < d \leq 10$ angstroms.

Reaction name	Chemical equations	k_{cat} (s^{-1})
3-phosphoglycerate kinase	ADP + BPG $\xrightleftharpoons{PGK1}$ ATP + P3G	58.6
enolase	P2G $\xrightleftharpoons{ENO1}$ PEP	7.6
	P2G $\xrightleftharpoons{ENO2}$ PEP	19.87
fructosebisphosphate aldolase	F16bP $\xrightleftharpoons{FBA1}$ DHAP + GAP	4.14
glyceraldehyde phosphate dehydrogenase	GAP + NAD $\xrightleftharpoons{TDH1}$ BPG + NADH	19.12
	GAP + NAD $\xrightleftharpoons{TDH2}$ BPG + NADH	8.63
	GAP + NAD $\xrightleftharpoons{TDH3}$ BPG + NADH	18.16
glycerol 3-phosphatase	G3P $\xrightarrow{HOR2}$ GLY	161.38
	G3P $\xrightarrow{RHR2}$ GLY	17.26
glycerol 3-phosphate dehydrogenase	DHAP + NADH $\xrightleftharpoons{GPD1}$ G3P + NAD	114.6
	DHAP + NADH $\xrightleftharpoons{GPD2}$ G3P + NAD	987.3
hexokinase	GLC + ATP $\xrightarrow{HXK1}$ G6P + ADP	10.2
	GLC + ATP $\xrightarrow{HXK2}$ G6P + ADP	63.1
	GLC + ATP $\xrightarrow{GLK1}$ G6P + ADP	0.07
phosphofructokinase	ATP + F6P $\xrightarrow{PFK1}$ ADP + F16bP	209.6
	ATP + F6P $\xrightarrow{PFK2}$ ADP + F16bP	209.6
phosphoglucomutase	G6P $\xrightleftharpoons{PGM1}$ G1P	39.12
	G6P $\xrightleftharpoons{PGM2}$ G1P	101.39
phosphoglucose isomerase	G6P $\xrightleftharpoons{PGI1}$ F6P	487.36
phosphoglyceromutase	P3G $\xrightleftharpoons{GPM1}$ P2G	400
pyruvate kinase	ADP + PEP $\xrightarrow{CDC19}$ ATP + PYR	20.15
	ADP + PEP $\xrightarrow{PYK2}$ ATP + PYR	0
T6P synthase	G6P + UDG $\xrightarrow{TPS1}$ T6P + UDP	145.49
T6P phosphatase	T6P $\xrightarrow{TPS2}$ TRH	879.75
triosephosphate isomerase	DHAP $\xrightleftharpoons{TPI1}$ GAP	564.38
UDP glucose phosphorylase	G1P + UTP $\xrightarrow{UGP1}$ UDG	2137.21

Table S2. Table of the reactions gained from the Smallbone2103 - Iteration 18 model to define the ABM underlying our simulations. As explained in the Methods section of the article, they represent a subset of all the Smallbone2013 reactions, specifically those for which the enzymatic concentration is provided and those not involved in the transformation of pyruvate to ethanol. The reactions are shown in alphabetic order; the related k_{cat} values are also reported.

- A perception distance of 300 Å has been chosen as the average length to simulate the existence of long-range forces among biomolecules (considering that the size of the simulation volume of our study is 1000 cubic angstroms). A perception sphere of this size is modelled with four different interaction probability intervals. Specifically, let p be the probability of interaction, d_{per} the perception distance and d_m the distance of the metabolite from the centre of the sphere representing the perceiving enzyme (all the lengths expressed in angstroms):
  - if $d_m \leq \frac{1}{4}\, d_{per}$, then $p = 1$
  - if $\frac{1}{4}\, d_{per} < d_m \leq \frac{3}{4}\, d_{per}$, then $p = \frac{3}{4}$
  - if $\frac{3}{4}\, d_{per} < d_m \leq d_{per}$, then $p = \frac{1}{2}$

This modelling approach turned out to be a reasonable abstraction to represent the progressive reduction of the attraction strength exerted by the enzyme on a cognate metabolite as the distance between the two molecules increases. We remark that we simulated three different types of systems in which specific short or long-range forces drive molecular interactions; therefore, in the AMBs defined for glycolysis, only one type of perception radius is allowed per modelled system.

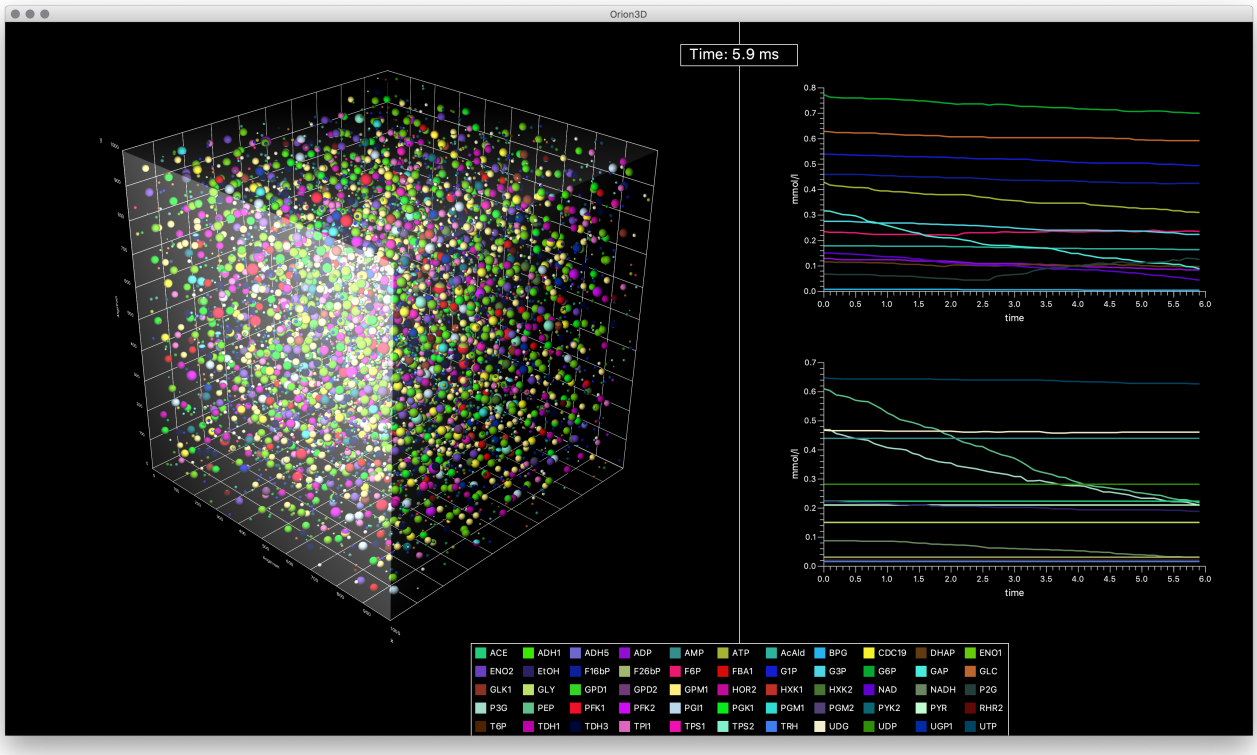

Figure S3. 3D interface of the agent-based simulator. The cube representing the volume of simulation has a side of 1000 Å. The interface shows the position of every molecule instant by instant. It is also possible to highlight the metabolites perceived by each enzyme at a specific time step of the simulation. On the right, the plots of the species concentration changes over time is generated for the first 5.9 ms of simulation. At the bottom of the interface, a legend associates each molecule with its corresponding colour.

3 Plots of the Concentration Changes over Time

This section provides some of the plots generated for the study proposed in the paper. Precisely, we report the concentration changes over time of the metabolites (Subsection 3.1) and a selection of the complex formations (Subsection 3.2). We choose specifically these plots since they are relevant to highlight the differences of the electrostatic and electromagnetic potentials modelled through our agent-based approach, as described in the article and in Section 2.4 of this supplementary document.

We ran the simulations on cloud-based virtual machines powered by 8 vCPUs and 32 GB of memory. With these hardware resources, simulating 0.1 seconds requires roughly 24 hours. For this reason, we chose 1 second (about ten days of simulation) as the standard time interval for our study. Even if it could be too short for observing some biological phenomena, such as the effects of enzyme activations and inhibitions, it turned out to be sufficient to highlight the impact of the long-distance electrodynamic interactions on the glycolytic pathway.

In what follows, we refer to each type of simulation as:

- `300 Å simulation`, representing a system where molecular interactions are driven by long-range forces;
- `10 Å simulation`, based on the model of a system where the Debye screening limits the capability of biomolecules to perceive each other;
- `5 Å simulation`, reproducing a biochemical system whose reactions rely only on random encounters and chemical affinity.

This convention is based on the *perception distances* that characterise the space inside which an enzyme can identify a cognate metabolite, as explained in Section 2.4 of this supplementary document.

3.1 Metabolite Concentration Changes

The following plots represent the molar concentration changes, observable in the interval of 1 second, of the metabolites simulated during our study. We exclude from the plots the `AMP` and the `F26bP` because, although they are present in the simulated environment, they are involved in enzyme regulation, a feature we have not yet modelled; their concentrations, thus, remain constant. To make each plot more readable, we define four subsets of metabolites (a complete list of which can be found in Table S1). According to the Smallbone2013 - Iteration 18 model[15], all of them are already present in the environment at the beginning of the glycolytic process.

Every kind of simulation produces plots with common properties; *therefore, we prefer to describe and compare such features in this introductory discussion instead of providing redundant captions for each figure.*

Observing the provided plots, we can notice the high rates through which the `300 Å simulation` produces or consumes the metabolites in the environment; this behaviour is less evident in the `10 Å simulation` and almost absent in the `5 Å simulation`. We largely discuss the reasons behind these phenomena in the article, where, in particular, we explain the concentration variations of `ADP`, `ATP`, `NADH` and `F16bP`; their plots are shown in the following Figs. S4 and S6. To summarise some of our findings, we can say that, by limiting the molecular interactions to those allowed by short-range van der Waals-like potentials (5 Å perception distance), most enzymes cannot bind part of their substrate. For example, in the plots generated by the `5 Å simulation`, the number of `ATP` molecules can only increase because, during the preparation phase of glycolysis, neither the `hexokinases` (`HXK`)–and `glucokinases` (`GLK`)–nor the `phosphofructokinases` (`PFK`) are able to bind this metabolite and complete the catalysis of their respective reactions; `ATP` is instead produced in the payoff phase. This particular aspect is better shown in the next subsection, which provides the plots of the complexes formation. Similarly, the concentration of `NADH` never changes during the entire interval of the `5 Å simulation` because the `glycerol-3-phosphate dehydrogenase` (`GDP`) is not able to bind this molecule (see Figs. S6, S11, and S12).

In general, the concentration curves of the `5 Å simulation`, besides showing, for several species, no changes in metabolite amounts, have two peculiar trends; precisely, they can:

1. increase/decrease until they reach a plateau (as in the case of `GLC`, in Fig. S5);
2. get to a value after which they roughly oscillate in a concentration interval during the rest of the simulation (a behaviour characterising `F6P`, in Fig. S4, and `G6P`, in Fig. S5).

The two situations are linked to the inability of specific enzymes to saturate when allowed to perceive their cognate metabolites through a small perception sphere. In the first case, they bind their substrate until all the enzymes of the same kind are partly saturated (i.e., forming the dual-complexes defined in Section 2.1); at this point, they are not able to complete the catalysis of the reaction, and the substrate concentration stabilises at the reached

value. The second case is due to a similar reason but relates to metabolites also involved in reversible reactions that do not require additional substrate (e.g., an energy donor) to be catalysed. Instead of reaching a plateau, when the partly-saturated enzyme cannot consume them, they start to "move back and forth" in the interconversion process of the reversible reaction, showing the oscillations mentioned above. The most relevant examples of these phenomena are the reactions catalysed by HXK (and GLK) and the one carried out by PFK:

$$\begin{aligned}
&\texttt{hexokinase:} && \texttt{GLC} + \texttt{ATP} \xrightarrow{\texttt{HXK}} \texttt{G6P} + \texttt{ADP} \\
&\texttt{glucokinase:} && \texttt{GLC} + \texttt{ATP} \xrightarrow{\texttt{GLK}} \texttt{G6P} + \texttt{ADP} \\
&\texttt{phosphofructokinase:} && \texttt{ATP} + \texttt{F6P} \xrightarrow{\texttt{PFK}} \texttt{ADP} + \texttt{F16bP}
\end{aligned}$$

Since, in the 5 Å simulation, PFK cannot bind ATP (Figure S10), all the molecules of this enzyme remain partly saturated, and the F6P still present in the environment begins to be interconverted in G6P by the phosphoglucose isomerase (PGI1–see Table S2). Since HXKs and GLKs also reach a point in which they are all partly saturated (Figure S8) and cannot produce G6P from glucose, the concentration of G6P starts to oscillate. The phosphoglucomutase (PGM), which interconverts G6P in G1P, equally participates in this process (Figure 2 of the article may help the reader visualise these behaviours because it graphically reproduces the sequence of glycolysis reactions).

In both plateaus and oscillations, the glycolytic process is blocked by the reactions catalysed by enzymes unable to saturate.

Conversely, the concentration changes of the 10 Å simulation have trends similar to those of the 300 Å simulation, although they show significantly lower rates. Among the others, it is important to notice this property in glucose (Figure S5), pyruvate (Figure S6), and in the products of the branches considered in our models, that is, glycerol (Figure S5) and trehalose (Figure S7).

We also provide the plots generated by a numerical time-course simulation, carried out through Copasi[24], of the Smallbone2013 kinetic model[15]. These plots are shown just for comparison; in the article, we propose various considerations over the limitations of this type of modelling and simulation approach. The main discrepancy with the agent-based simulations is the inability of the kinetic model to grasp the fluctuations of the species concentrations, producing more homogeneous curves. Moreover, a system of differential equations is less flexible than an agent-based model and removing enzyme regulation would have compromised its consistency, making the numerical simulation impossible. This property resulted in concentration changes closer to those observable at steady state, a condition unlikely to be reached by our agent-based simulations (see the article for details). If we exclude these differences, the numerical simulation, in some cases, shows concentration trends loosely closer to those observable in the 300 Å simulation and 10 Å simulation (see the GLC curve in Fig. S5), while, in others, to the variations produced by the 5 Å simulation. In several cases, they are completely different from the corresponding concentration changes generated by the agent-based simulations.

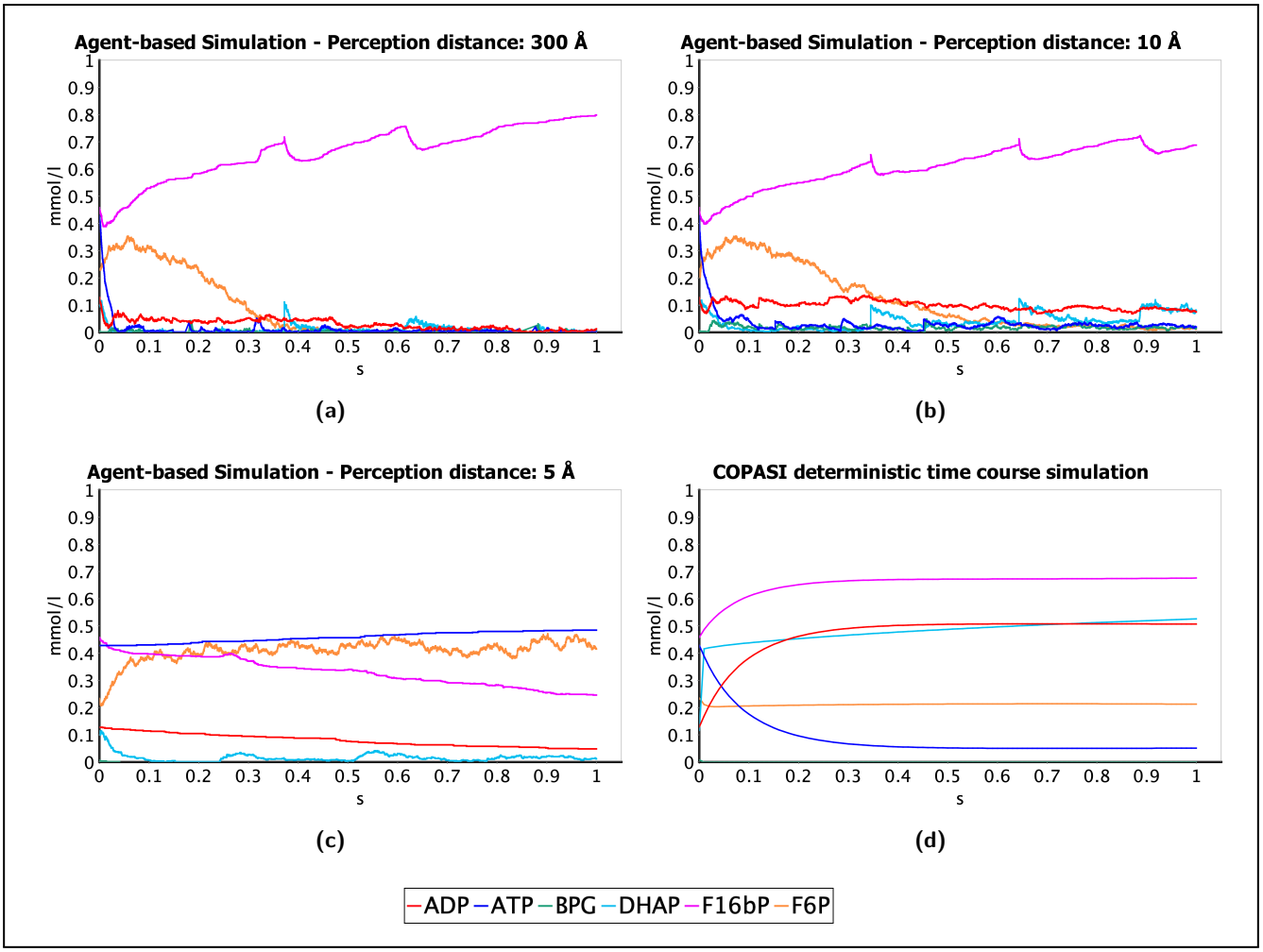

Figure S4. Concentration changes over time of `adenosine diphosphate` (`ADP`), `adenosine triphosphate` (`ATP`), `1,3-bisphosphoglycerate` (`BPG`), `dihydroxyacetone phosphate` (`DHAP`), `fructose 1,6-bisphosphate` (`F16bP`) and `fructose 6-phosphate` (`F6P`).

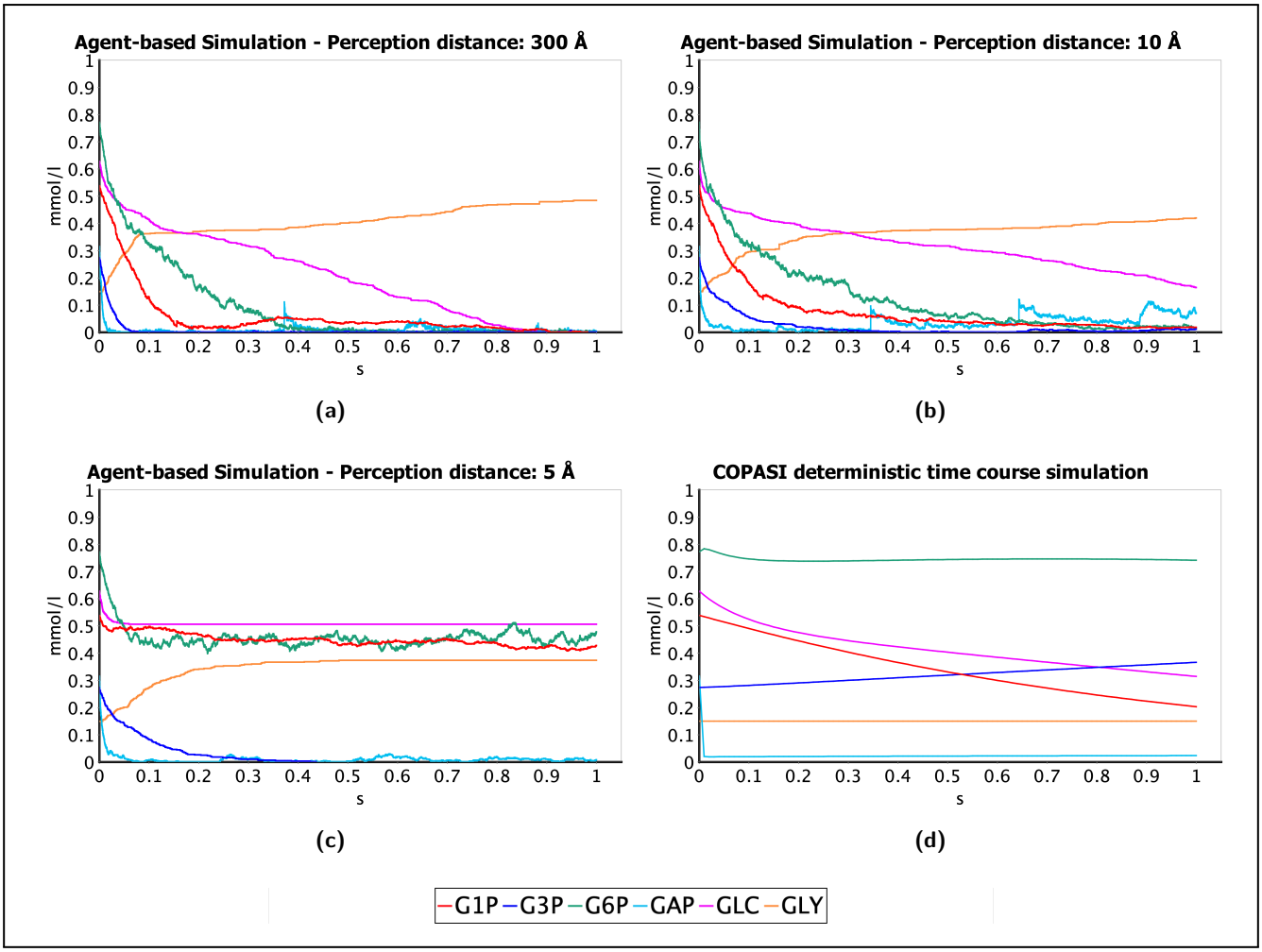

Figure S5. Concentration changes over time of `glucose 1-phosphate` (`G1P`), `glycerol 3-phosphate` (`G3P`), `glucose 6-phosphate` (`G6P`), `glyceraldehyde 3-phosphate` (`GAP`), `glucose` (`GLC`) and `glycerol` (`GLY`).

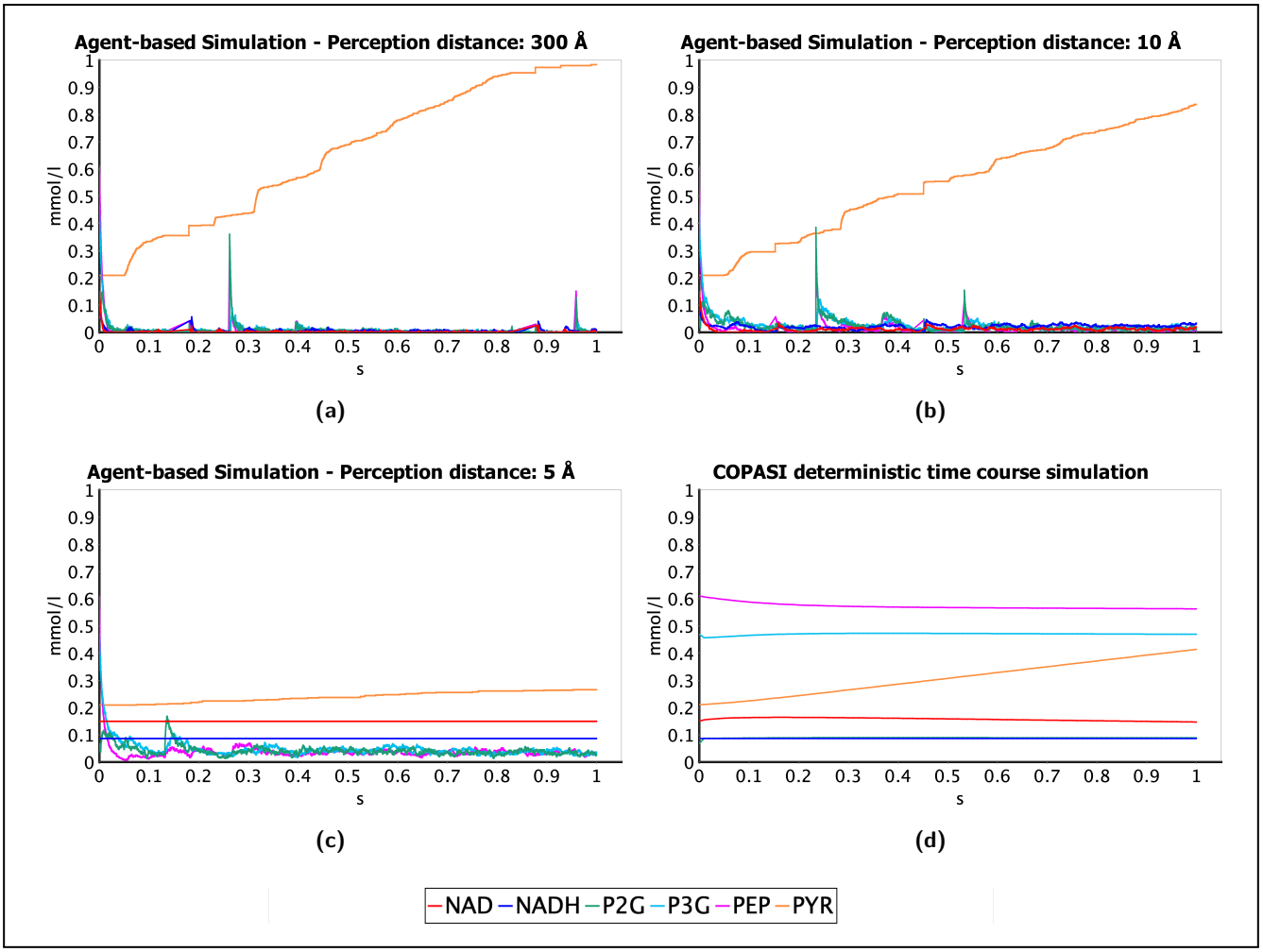

Figure S6. Concentration changes over time of `nicotinamide adenine dinucleotide` (`NAD`), `nicotinamide adenine dinucleotide plus hydrogen` (`NADH`), `2-phosphoglycerate` (`P2G`), `3-phosphoglycerate` (`P3G`), `phosphoenolpyruvate` (`PEP`) and `pyruvate` (`PYR`).

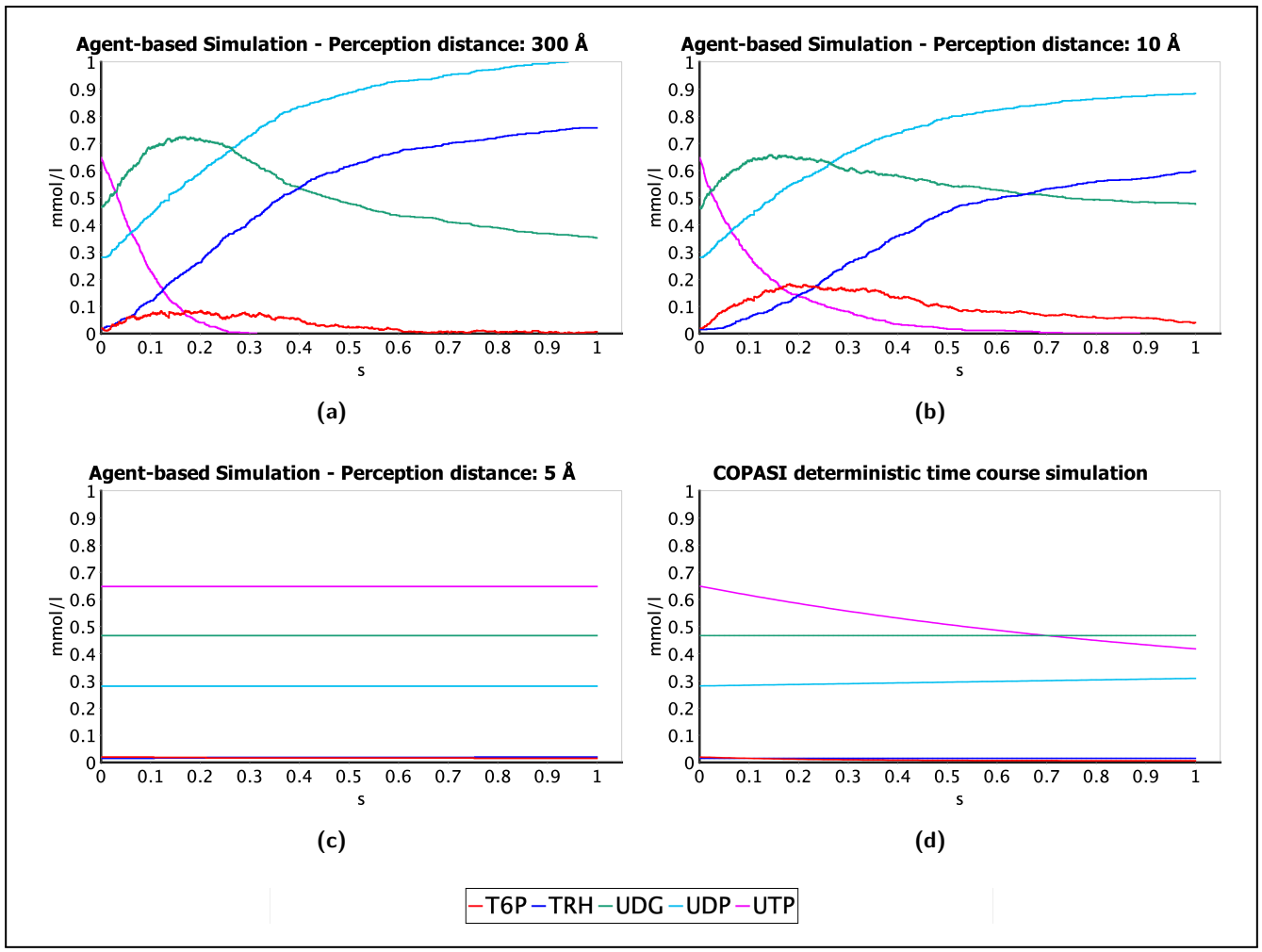

Figure S7. Concentration changes over time of `trehalose 6-phosphate` (`T6P`), `trehalose` (`TRH`), `uridine diphosphate glucose` (`UDG`), `uridine diphosphate` (`UDP`) and `uridine triphosphate` (`UTP`).

3.2 Comparison of Relevant Complexes Formation

In this subsection, we provide a comparison of the plots of metabolites and complexes involved, during the 5, 10 and 300 Å simulations, in the following reactions:

- phosphorylation of glucose (GLC) to glucose-6-phosphate (G6P), performed by the hexokinase (HXK) and the glucokinase (GLK);
- phosphorylation of fructose 6-phosphate (F6P) to fructose 1,6-bisphosphate (F16bP), carried out by the phosphofructokinase (PFK);
- conversion of dihydroxyacetone phosphate (DHAP) to glycerol 3-phosphate (G3P), catalysed by glycerol-3-phosphate dehydrogenase (GDP).

The reason for this choice is to show how the agent-based approach can highlight the limitations of a system in which molecular interactions are driven only by random encounters and chemical affinity (that is, the 5 Å simulation). This is possible thanks to the capability of the agent-based simulations to reproduce local molecular interactions and thus the formation of partly saturated enzymes. A similar analysis cannot be carried out over the numerical time-course simulations, because they consider each reaction as a mathematical function from reactants to products, not explicitly taking into account the effects of the local interactions. For this reason, all the plots generated through Copasi cannot show the concentration changes of the complexes (whether they are enzymes partly or fully saturated).

In the proposed reactions, we can observe that, differently from the 300 and 10 Å simulations, the 5 Å simulation does not allow the binding of the enzymes with the needed energy donor (ATP for the reactions catalysed by HXK, GLK and PFK, and NADH for the reaction performed by GDP). This property produces the peculiar concentration changes discussed in the previous subsection and in the paper. To make such a phenomenon more evident, each plot is provided with a dedicated legend; in this way, at a glance, it is possible to notice how fully saturated enzymes are not present in the plots of the 5 Å simulation.

For each reaction, we show the concentration changes of reactants, products and complexes made by every isoenzyme considered in our model of glycolysis (see Table S2 for the related chemical equations).

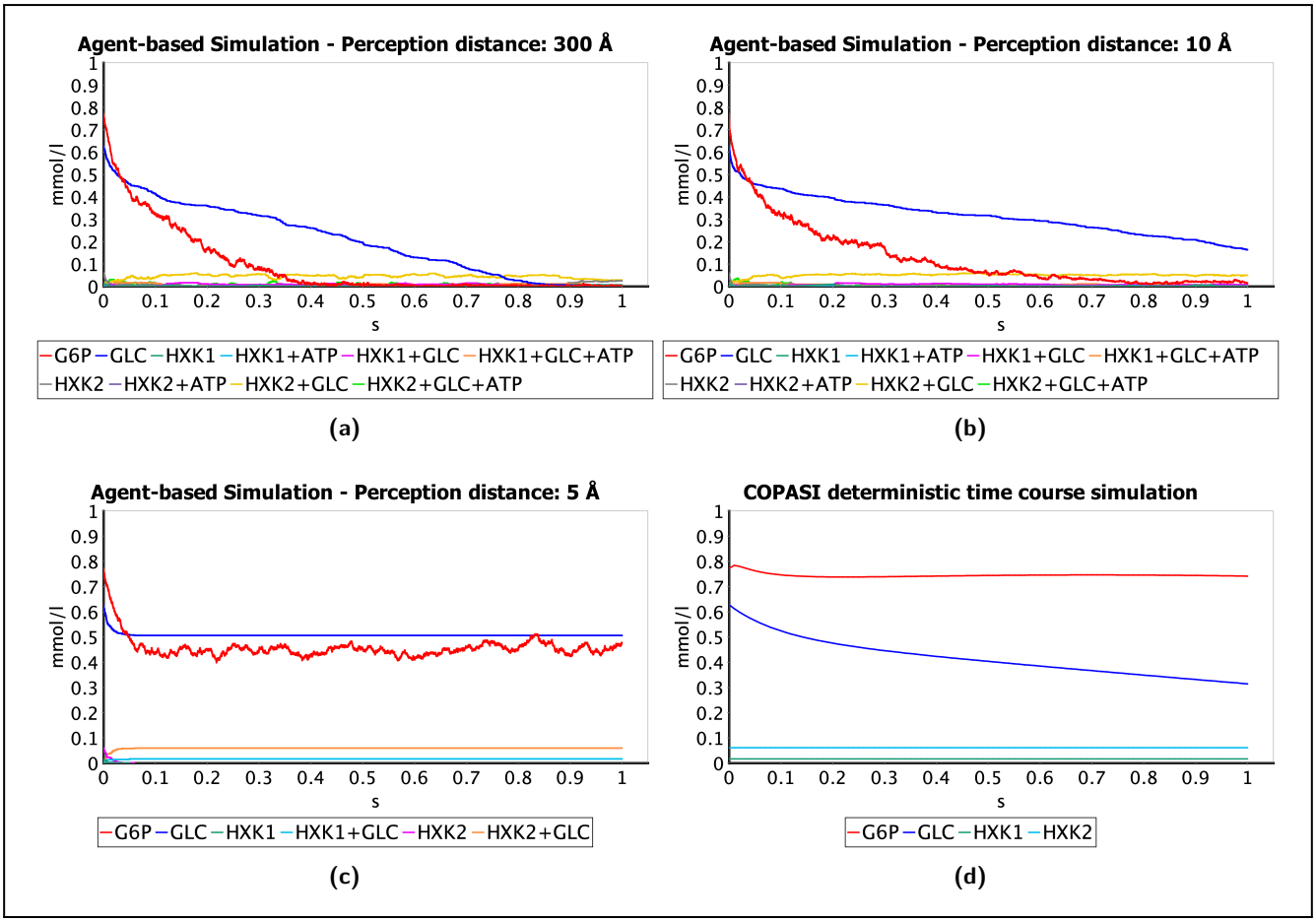

Figure S8. Phosphorylation of `glucose` (`GLC`) to `glucose-6-phosphate` (`G6P`), performed by the hexokinase (`HXK1` and `HXK2` isoenzymes). The reaction requires the energy generated by the hydrolysis of `ATP` to `ADP`.

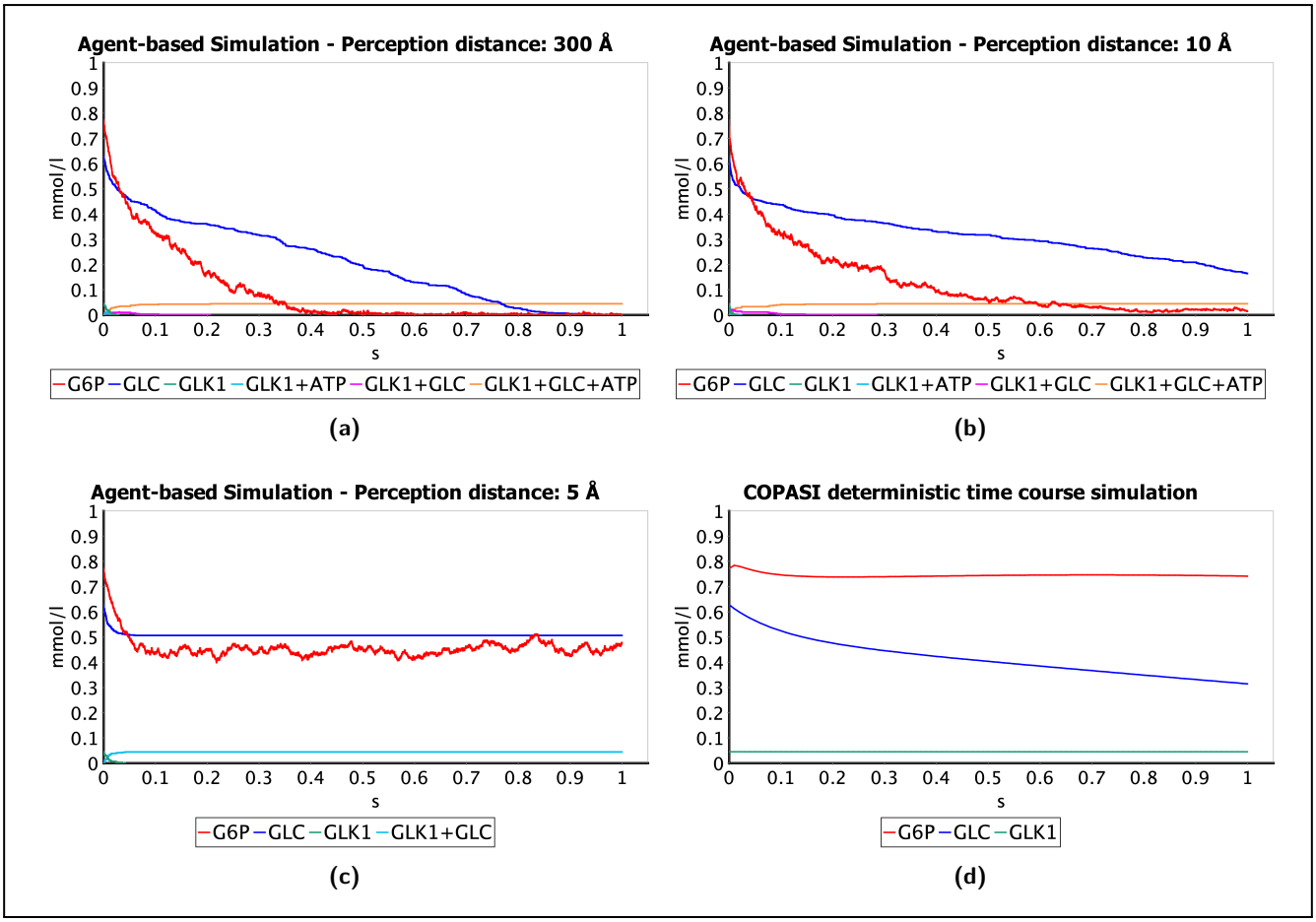

Figure S9. Phosphorylation of `glucose` (`GLC`) to `glucose-6-phosphate` (`G6P`), performed by the `glucokinase` (`GLK1`). The reaction requires the energy generated by the hydrolysis of `ATP` to `ADP`.

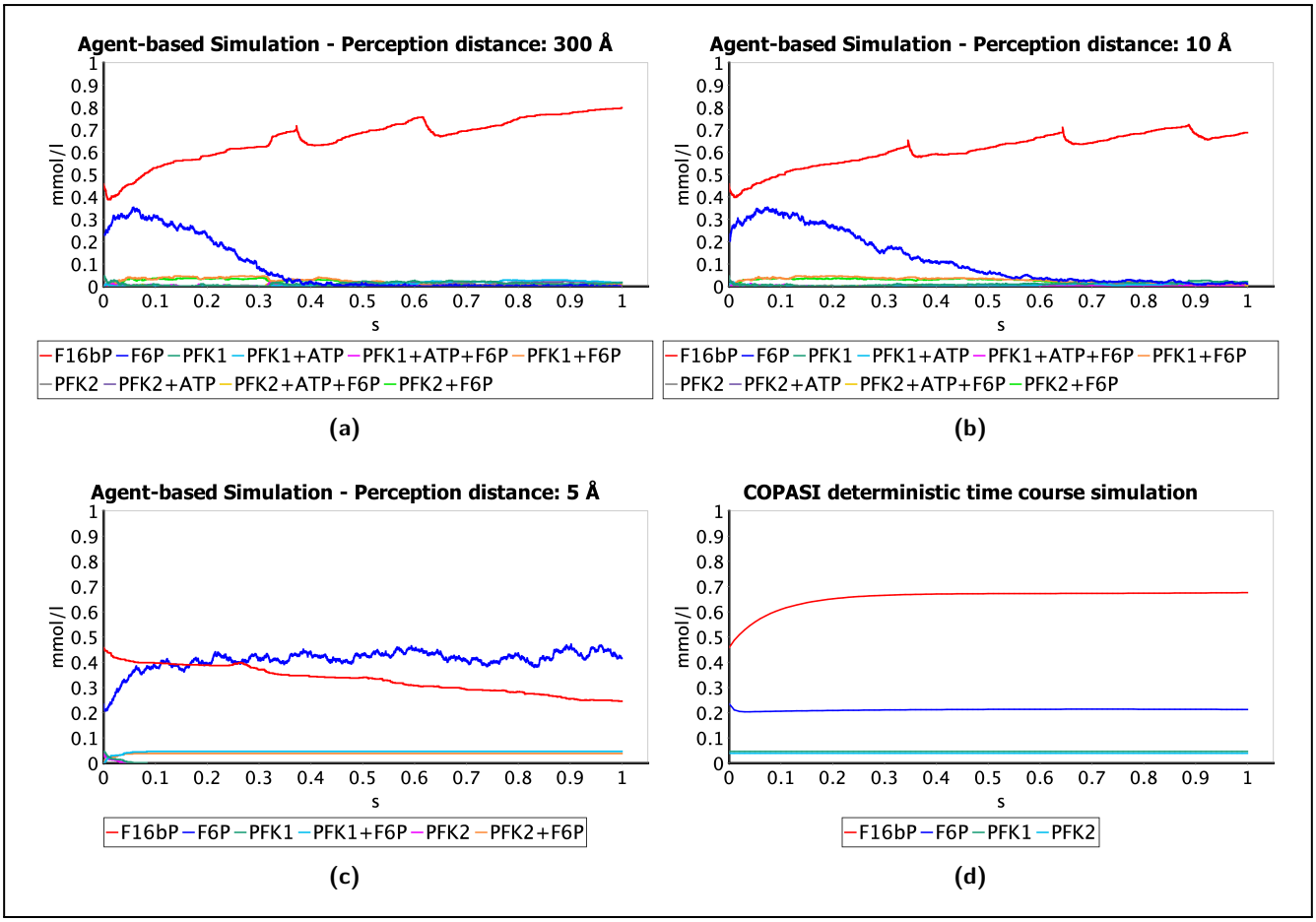

Figure S10. Phosphorylation of `fructose 6-hosphate` (`F6P`) to `fructose 1,6-bisphosphate` (`F16bP`), carried out by the `phosphofructokinase` (`PFK1` and `PFK2` isoenzymes). The reaction requires the energy generated by the hydrolysis of `ATP` to `ADP`.

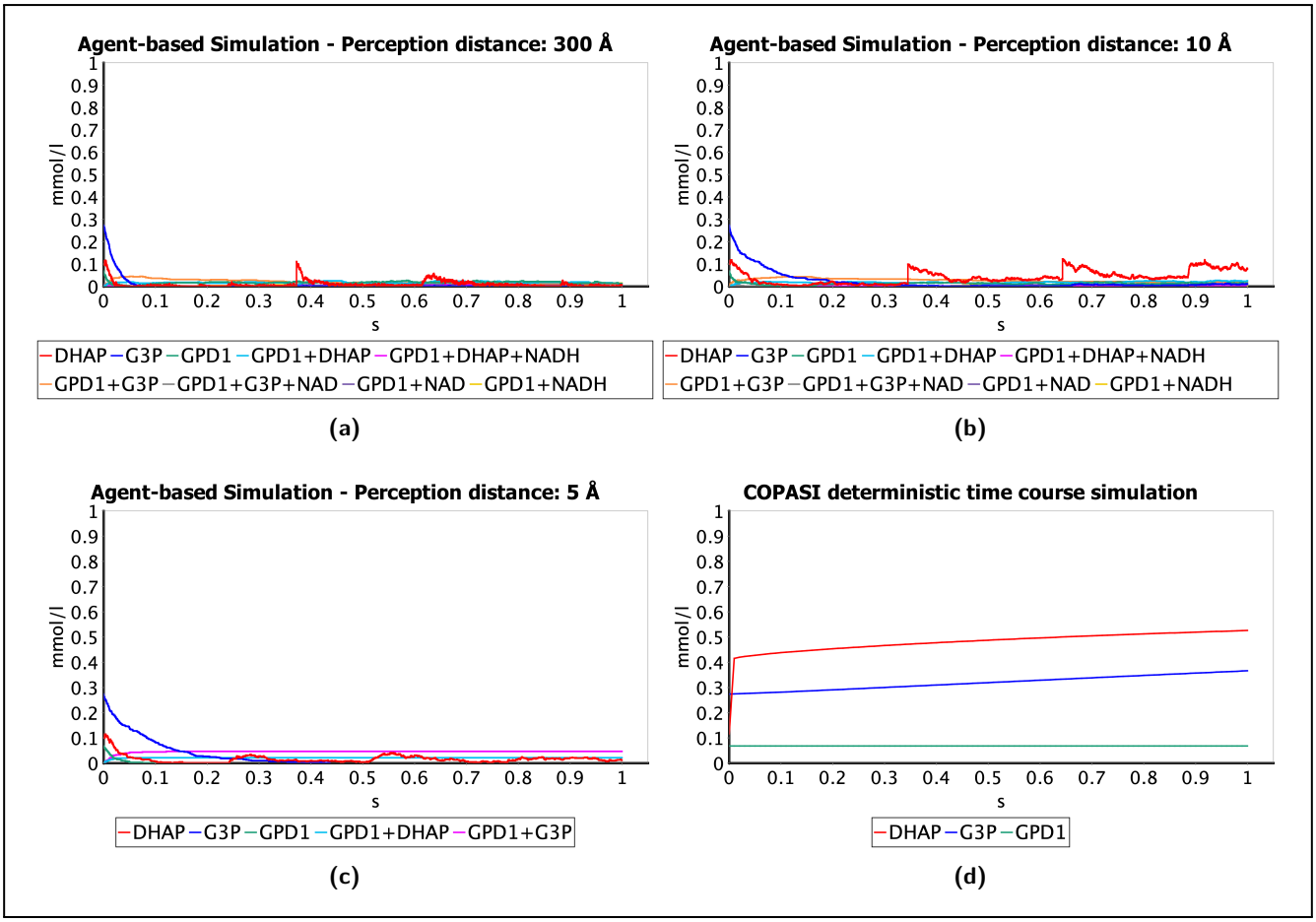

Figure S11. Conversion of `dihydroxyacetone phosphate` (`DHAP`) to `glycerol 3-phosphate` (`G3P`), catalysed by `glycerol-3-phosphate dehydrogenase` (`GPD1` isoenzyme). To be performed, the reaction must be coupled with the conversion of `NADH` to `NAD`.

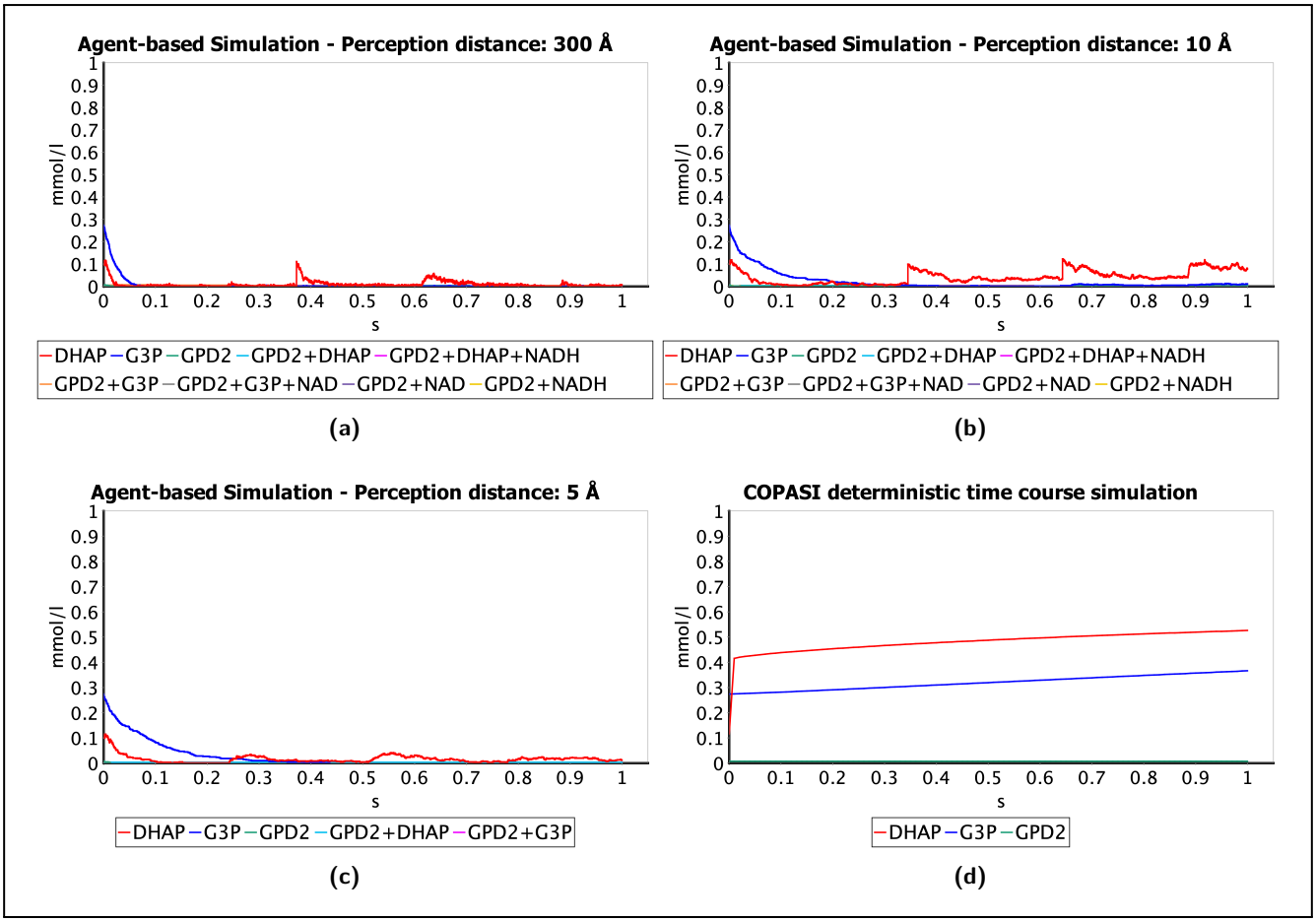

Figure S12. Conversion of `dihydroxyacetone phosphate` (`DHAP`) to `glycerol 3-phosphate` (`G3P`), catalysed by `glycerol-3-phosphate dehydrogenase` (`GPD2` isoenzyme). To be performed, the reaction must be coupled with the conversion of `NADH` to `NAD`.

References

1. Lehninger, A. L., Nelson, D. L. & Cox, M. M. *Lehninger Principles of Biochemistry* (W.H. Freeman, New York, 2005), 4th ed edn.
2. Johnson, K. A. & Goody, R. S. The Original Michaelis Constant: Translation of the 1913 Michaelis–Menten Paper. *Biochemistry* **50**, 8264–8269, DOI: 10.1021/bi201284u (2011).
3. Briggs, G. E. & Haldane, J. B. S. A Note on the Kinetics of Enzyme Action. *Biochem. J.* **19**, 338–339, DOI: 10.1042/bj0190338 (1925).
4. Piangerelli, M., Maestri, S. & Merelli, E. Visualising 2-Simplex formation in metabolic reactions. *J. Mol. Graph. Model.* **97**, 107576, DOI: 10.1016/j.jmgm.2020.107576 (2020).
5. Aceto, L., Ingólfsdóttir, A., Larsen, K. & Srba, J. *Reactive Systems: Modelling, Specification and Verification* (Cambridge University Press, 2007).
6. Maestri, S. & Merelli, E. Algebraic Characterisation of Non-Coding RNA. In Cazzaniga, P., Besozzi, D., Merelli, I. & Manzoni, L. (eds.) *Computational Intelligence Methods for Bioinformatics and Biostatistics*, vol. 12313, 145–158, DOI: 10.1007/978-3-030-63061-4_14 (Springer International Publishing, Cham, 2020).
7. Keller, R. M. Formal verification of parallel programs. *Commun. ACM* **19**, 371–384 (1976).
8. Andersen, J. R. *et al.* CAAL: Concurrency workbench, Aalborg Edition. In *Theoretical Aspects of Computing - ICTAC 2015*, 573–582, DOI: 10.1007/978-3-319-25150-9_33 (Springer International Publishing, 2015).
9. Hucka, M. *et al.* The systems biology markup language (SBML): A medium for representation and exchange of biochemical network models. *Bioinformatics* **19**, 524–531, DOI: 10.1093/bioinformatics/btg015 (2003).
10. Forcato, C. Orion: A spatial multiagent system framework for cellular simulation. Implementation of molecular movement at the mesoscale. MSc Thesis UNICAM-CS-2004-2, Universita' di Camerino (2005).
11. Mattioni, M. Orion: A multiagent system framework for cellular simulation. Implementation of metabolic reaction at the mesoscale. MSc Thesis UNICAM-CS-2005-2, Universita' di Camerino (2005).
12. Baldoncini, A. Orion: A spatial multi agent system framework for computational cellular dynamics of metabolic pathways. MSc Thesis UNICA-CS-2004-1, Universita' di Camerino (2004).
13. Angeletti, M. *et al.* Spatial behavioral modeling and simulation of metabolic pathways with Orion. In *IV Bioinformatics ITalian Society Meeting (BITS 2007)*, 70 (Napoli, Italy, 2006).
14. Cannata, N., Corradini, F. & Merelli, E. Multiagent modelling and simulation of carbohydrate oxidation in cell. *Int J Model. Identif. Control.* **3**, DOI: 10.1504/IJMIC.2008.018191 (2008).
15. Smallbone, K. *et al.* A model of yeast glycolysis based on a consistent kinetic characterisation of all its enzymes. *FEBS Lett.* **587**, 2832–2841, DOI: 10.1016/j.febslet.2013.06.043 (2013).
16. World Wide Web Consortium (W3C). Extensible Markup Language (XML) 1.0 (Fifth Edition) (2013).
17. Hastings, J. *et al.* ChEBI in 2016: Improved services and an expanding collection of metabolites. *Nucleic Acids Res* **44**, D1214–D1219, DOI: 10.1093/nar/gkv1031 (2016).
18. The UniProt Consortium. UniProt: A worldwide hub of protein knowledge. *Nucleic Acids Res.* **47**, D506–D515, DOI: 10.1093/nar/gky1049 (2019).
19. Richards, F. M. AREAS, VOLUMES, PACKING, AND PROTEIN STRUCTURE. *Annu. Rev. Biophys. Bioeng.* **6**, 151–176, DOI: 10.1146/annurev.bb.06.060177.001055 (1977).
20. Zamyatnin, A. Protein volume in solution. *Prog. Biophys. Mol. Biol.* **24**, 107–123, DOI: 10.1016/0079-6107(72)90005-3 (1972).
21. Harpaz, Y., Gerstein, M. & Chothia, C. Volume changes on protein folding. *Structure* **2**, 641–649, DOI: 10.1016/S0969-2126(00)00065-4 (1994).
22. Erickson, H. P. Size and Shape of Protein Molecules at the Nanometer Level Determined by Sedimentation, Gel Filtration, and Electron Microscopy. *Biol Proced Online* **11**, 32–51, DOI: 10.1007/s12575-009-9008-x (2009).
23. Milo, R., Jorgensen, P., Moran, U., Weber, G. & Springer, M. BioNumbers—the database of key numbers in molecular and cell biology. *Nucleic Acids Res.* **38**, D750–D753, DOI: 10.1093/nar/gkp889 (2010).
24. Hoops, S. *et al.* COPASI–a COmplex PAthway SImulator. *Bioinformatics* **22**, 3067–3074, DOI: 10.1093/bioinformatics/btl485 (2006).

Appendices

A1 XML input file

The input of the agent-based simulator described in the article is an XML file generated, through a dedicated module of the simulator, from the SBML representation of the Smallbone2013 - Iteration 18 model[15]. The structure and properties of this file are specified in Section 2.2 of this supplementary document and in the Methods section of the article. This appendix provides the actual XML file taken as input for running the simulations discussed in the manuscript.

```
<?xml version="1.0" encoding="UTF-8"?>
<orion xmlns="https://github.com/stefanomaestri/orion/tree/main/xml">
  <!--Pathway settings-->
  <pathway id="glycolysis" organism="Saccharomyces_cerevisiae">
    <!--Unit definitions-->
    <listOfUnits>
      <unitDefinition id="mmol">
        <unit exponent="1" kind="mole" multiplier="1" scale="-3"/>
      </unitDefinition>
      <unitDefinition id="mM">
        <unit exponent="1" kind="mole" multiplier="1" scale="-3"/>
        <unit exponent="-1" kind="litre" multiplier="1" scale="0"/>
      </unitDefinition>
      <unitDefinition id="mM_per_second">
        <unit exponent="1" kind="mole" multiplier="1" scale="-3"/>
        <unit exponent="-1" kind="litre" multiplier="1" scale="0"/>
        <unit exponent="-1" kind="second" multiplier="1" scale="0"/>
      </unitDefinition>
      <unitDefinition id="per_second">
        <unit exponent="-1" kind="second" multiplier="1" scale="0"/>
      </unitDefinition>
      <unitDefinition id="per_mM_per_second">
        <unit exponent="-1" kind="mole" multiplier="1" scale="-3"/>
        <unit exponent="1" kind="litre" multiplier="1" scale="0"/>
        <unit exponent="-1" kind="second" multiplier="1" scale="0"/>
      </unitDefinition>
      <unitDefinition id="per_mmol">
        <unit exponent="-1" kind="mole" multiplier="1" scale="-3"/>
      </unitDefinition>
      <unitDefinition id="dalton">
        <unit exponent="1" kind="kilogram" multiplier="1.66054" scale="-27"/>
      </unitDefinition>
      <unitDefinition id="attolitre">
        <unit exponent="1" kind="litre" multiplier="1" scale="-18"/>
      </unitDefinition>
      <unitDefinition id="pascal_second">
        <unit exponent="1" kind="kilogram" multiplier="1" scale="0"/>
        <unit exponent="-1" kind="metre" multiplier="1" scale="0"/>
        <unit exponent="-1" kind="second" multiplier="1" scale="0"/>
      </unitDefinition>
      <unitDefinition id="angstrom">
        <unit exponent="1" kind="metre" multiplier="1" scale="-10"/>
      </unitDefinition>
    </listOfUnits>
    <listOfMolecules>
      <!--List of metabolites-->
      <!--metabolite - ChEBI name: ADP; resource: http://identifiers.org/chebi/CHEBI:16761 -->
      <molecule compartment="cell" id="ADP" name="ADP" type="Metabolite">
        <molecularWeight unit="dalton">427.03</molecularWeight>
        <initialConcentration unit="mM">0.129</initialConcentration>
      </molecule>
      <!--metabolite - ChEBI name: ATP; resource: http://identifiers.org/chebi/CHEBI:15422 -->
      <molecule compartment="cell" id="ATP" name="ATP" type="Metabolite">
        <molecularWeight unit="dalton">507.0</molecularWeight>
        <initialConcentration unit="mM">0.429</initialConcentration>
      </molecule>
      <!--metabolite - ChEBI name: 3-phospho-D-glyceroyl_dihydrogen_phosphate; resource: http://
    identifiers.org/chebi/CHEBI:16001 -->
```

```
    <molecule compartment="cell" id="BPG" name="1,3-bisphosphoglycerate" type="Metabolite">
      <molecularWeight unit="dalton">265.96</molecularWeight>
      <initialConcentration unit="mM">0.007368734998656 02</initialConcentration>
    </molecule>
    <!--metabolite - ChEBI name: dihydroxyacetone_phosphate; resource: http://identifiers.org/chebi/CHEBI:16108 -->
    <molecule compartment="cell" id="DHAP" name="dihydroxyacetone_phosphate" type="Metabolite">
      <molecularWeight unit="dalton">170.0</molecularWeight>
      <initialConcentration unit="mM">0.11613768527467001</initialConcentration>
    </molecule>
    <!--metabolite - ChEBI name: beta-D-fructofuranose_1,6-bisphosphate; resource: http://identifiers.org/chebi/CHEBI:28013 -->
    <molecule compartment="cell" id="F16bP" name="fructose_1,6-bisphosphate" type="Metabolite">
      <molecularWeight unit="dalton">340.0</molecularWeight>
      <initialConcentration unit="mM">0.458321859006931</initialConcentration>
    </molecule>
    <!--metabolite - ChEBI name: beta-D-fructofuranose_6-phosphate; resource: http://identifiers.org/chebi/CHEBI:16084 -->
    <molecule compartment="cell" id="F6P" name="fructose_6-phosphate" type="Metabolite">
      <molecularWeight unit="dalton">260.03</molecularWeight>
      <initialConcentration unit="mM">0.235441221891221</initialConcentration>
    </molecule>
    <!--metabolite - ChEBI name: D-glucopyranose_1-phosphate; resource: http://identifiers.org/chebi/CHEBI:16077 -->
    <molecule compartment="cell" id="G1P" name="glucose_1-phosphate" type="Metabolite">
      <molecularWeight unit="dalton">260.03</molecularWeight>
      <initialConcentration unit="mM">0.539248506344921</initialConcentration>
    </molecule>
    <!--metabolite - ChEBI name: sn-glycerol_3-phosphate; resource: http://identifiers.org/chebi/CHEBI:15978 -->
    <molecule compartment="cell" id="G3P" name="glycerol_3-phosphate" type="Metabolite">
      <molecularWeight unit="dalton">172.01</molecularWeight>
      <initialConcentration unit="mM">0.274002929191284</initialConcentration>
    </molecule>
    <!--metabolite - ChEBI name: alpha-D-glucose_6-phosphate; resource: http://identifiers.org/chebi/CHEBI:17665 -->
    <molecule compartment="cell" id="G6P" name="glucose_6-phosphate" type="Metabolite">
      <molecularWeight unit="dalton">260.03</molecularWeight>
      <initialConcentration unit="mM">0.772483203645216</initialConcentration>
    </molecule>
    <!--metabolite - ChEBI name: D-glyceraldehyde_3-phosphate; resource: http://identifiers.org/chebi/CHEBI:29052 -->
    <molecule compartment="cell" id="GAP" name="glyceraldehyde_3-phosphate" type="Metabolite">
      <molecularWeight unit="dalton">170.0</molecularWeight>
      <initialConcentration unit="mM">0.315891028770503</initialConcentration>
    </molecule>
    <!--metabolite - ChEBI name: D-glucopyranose; resource: http://identifiers.org/chebi/CHEBI:4167 -->
    <molecule compartment="cell" id="GLC" name="glucose" type="Metabolite">
      <molecularWeight unit="dalton">180.06</molecularWeight>
      <initialConcentration unit="mM">0.6280001793382419</initialConcentration>
    </molecule>
    <!--metabolite - ChEBI name: NAD(+); resource: http://identifiers.org/chebi/CHEBI:15846 -->
    <molecule compartment="cell" id="NAD" name="NAD" type="Metabolite">
      <molecularWeight unit="dalton">664.12</molecularWeight>
      <initialConcentration unit="mM">0.15032903020153102</initialConcentration>
    </molecule>
    <!--metabolite - ChEBI name: 2-phospho-D-glyceric_acid; resource: http://identifiers.org/chebi/CHEBI:17835 -->
    <molecule compartment="cell" id="P2G" name="2-phosphoglycerate" type="Metabolite">
      <molecularWeight unit="dalton">185.99</molecularWeight>
      <initialConcentration unit="mM">0.0677379081099344</initialConcentration>
    </molecule>
    <!--metabolite - ChEBI name: 3-phospho-D-glyceric_acid; resource: http://identifiers.org/chebi/CHEBI:17794 -->
    <molecule compartment="cell" id="P3G" name="3-phosphoglycerate" type="Metabolite">
      <molecularWeight unit="dalton">185.99</molecularWeight>
      <initialConcentration unit="mM">0.469825011134444</initialConcentration>
    </molecule>
```

```
    <!--metabolite - ChEBI name: phosphoenolpyruvate; resource: http://identifiers.org/chebi/CHEBI:18021 -->
    <molecule compartment="cell" id="PEP" name="phosphoenolpyruvate" type="Metabolite">
      <molecularWeight unit="dalton">166.98</molecularWeight>
      <initialConcentration unit="mM">0.610005413358042</initialConcentration>
    </molecule>
    <!--metabolite - ChEBI name: pyruvate; resource: http://identifiers.org/chebi/CHEBI:15361 -->
    <molecule compartment="cell" id="PYR" name="pyruvate" type="Metabolite">
      <molecularWeight unit="dalton">87.01</molecularWeight>
      <initialConcentration unit="mM">0.210847140717419</initialConcentration>
    </molecule>
    <!--metabolite - ChEBI name: alpha,alpha-trehalose_6-phosphate; resource: http://identifiers.org/chebi/CHEBI:18283 -->
    <molecule compartment="cell" id="T6P" name="trehalose_6-phosphate" type="Metabolite">
      <molecularWeight unit="dalton">422.08</molecularWeight>
      <initialConcentration unit="mM">0.02</initialConcentration>
    </molecule>
    <!--metabolite - ChEBI name: UDP; resource: http://identifiers.org/chebi/CHEBI:17659 -->
    <molecule compartment="cell" id="UDP" name="UDP" type="Metabolite">
      <molecularWeight unit="dalton">404.0</molecularWeight>
      <initialConcentration unit="mM">0.2815</initialConcentration>
    </molecule>
    <!--metabolite - ChEBI name: UTP(4-); resource: http://identifiers.org/chebi/CHEBI:46398 -->
    <molecule compartment="cell" id="UTP" name="UTP" type="Metabolite">
      <molecularWeight unit="dalton">479.94</molecularWeight>
      <initialConcentration unit="mM">0.6491</initialConcentration>
    </molecule>
    <!--metabolite - ChEBI name: adenosine_5‘-monophosphate; resource: http://identifiers.org/chebi/CHEBI:16027 -->
    <molecule compartment="cell" id="AMP" name="AMP" type="Metabolite">
      <molecularWeight unit="dalton">347.06</molecularWeight>
      <initialConcentration unit="mM">0.44</initialConcentration>
    </molecule>
    <!--metabolite - ChEBI name: NADH; resource: http://identifiers.org/chebi/CHEBI:16908 -->
    <molecule compartment="cell" id="NADH" name="NADH" type="Metabolite">
      <molecularWeight unit="dalton">665.12</molecularWeight>
      <initialConcentration unit="mM">0.0867096979846952</initialConcentration>
    </molecule>
    <!--metabolite - ChEBI name: UDP-D-glucose; resource: http://identifiers.org/chebi/CHEBI:18066 -->
    <molecule compartment="cell" id="UDG" name="UDP_glucose" type="Metabolite">
      <molecularWeight unit="dalton">566.06</molecularWeight>
      <initialConcentration unit="mM">0.467246194874247</initialConcentration>
    </molecule>
    <!--metabolite - ChEBI name: beta-D-fructofuranose_2,6-bisphosphate; resource: http://identifiers.org/chebi/CHEBI:28602 -->
    <molecule compartment="cell" id="F26bP" name="fructose_2,6-bisphosphate" type="Metabolite">
      <molecularWeight unit="dalton">340.0</molecularWeight>
      <initialConcentration unit="mM">0.03</initialConcentration>
    </molecule>
    <!--metabolite - ChEBI name: D-glucopyranose; resource: http://identifiers.org/chebi/CHEBI:4167 -->
    <molecule compartment="extracellular" id="GLCx" name="glucose" type="Metabolite">
      <molecularWeight unit="dalton">180.06</molecularWeight>
      <initialConcentration unit="mM">0.74</initialConcentration>
    </molecule>
    <!--metabolite - ChEBI name: glycerol; resource: http://identifiers.org/chebi/CHEBI:17754 -->
    <molecule compartment="cell" id="GLY" name="glycerol" type="Metabolite">
      <molecularWeight unit="dalton">92.05</molecularWeight>
      <initialConcentration unit="mM">0.15</initialConcentration>
    </molecule>
    <!--metabolite - ChEBI name: alpha,alpha-trehalose; resource: http://identifiers.org/chebi/CHEBI:16551 -->
    <molecule compartment="cell" id="TRH" name="trehalose" type="Metabolite">
      <molecularWeight unit="dalton">342.12</molecularWeight>
      <initialConcentration unit="mM">0.0153878853696526</initialConcentration>
    </molecule>
    <!--List of enzymes and complexes-->
```

```
    <!--Reaction: Pyruvate kinase [CDC19]-->
    <!--enzyme - UniProt name: Pyruvate_kinase_1; resource: https://www.uniprot.org/uniprot/P00549 -->
    <molecule compartment="cell" id="CDC19" name="CDC19" type="Enzyme">
      <molecularWeight unit="dalton">54545.0</molecularWeight>
      <initialConcentration unit="mM">0.20483901071712</initialConcentration>
    </molecule>
    <molecule compartment="cell" id="CDC19+ADP" name="CDC19+ADP" type="Complex">
      <molecularWeight unit="dalton">54972.03</molecularWeight>
      <initialConcentration unit="mM">0</initialConcentration>
    </molecule>
    <molecule compartment="cell" id="CDC19+PEP" name="CDC19+phosphoenolpyruvate" type="Complex">
      <molecularWeight unit="dalton">54711.98</molecularWeight>
      <initialConcentration unit="mM">0</initialConcentration>
    </molecule>
    <molecule compartment="cell" id="CDC19+ADP+PEP" name="CDC19+ADP+phosphoenolpyruvate" type="Complex">
      <molecularWeight unit="dalton">55139.01</molecularWeight>
      <initialConcentration unit="mM">0</initialConcentration>
    </molecule>
    <!--Reaction: Enolase [ENO1]-->
    <!--enzyme - UniProt name: Enolase_1; resource: https://www.uniprot.org/uniprot/P00924 -->
    <molecule compartment="cell" id="ENO1" name="ENO1" type="Enzyme">
      <molecularWeight unit="dalton">46816.0</molecularWeight>
      <initialConcentration unit="mM">0.686371954155832</initialConcentration>
    </molecule>
    <molecule compartment="cell" id="ENO1+P2G" name="ENO1+2-phosphoglycerate" type="Complex">
      <molecularWeight unit="dalton">47001.99</molecularWeight>
      <initialConcentration unit="mM">0</initialConcentration>
    </molecule>
    <molecule compartment="cell" id="ENO1+PEP" name="ENO1+phosphoenolpyruvate" type="Complex">
      <molecularWeight unit="dalton">46982.98</molecularWeight>
      <initialConcentration unit="mM">0</initialConcentration>
    </molecule>
    <!--Reaction: Enolase [ENO2]-->
    <!--enzyme - UniProt name: Enolase_2; resource: https://www.uniprot.org/uniprot/P00925 -->
    <molecule compartment="cell" id="ENO2" name="ENO2" type="Enzyme">
      <molecularWeight unit="dalton">46914.0</molecularWeight>
      <initialConcentration unit="mM">0.197444629317817</initialConcentration>
    </molecule>
    <molecule compartment="cell" id="ENO2+P2G" name="ENO2+2-phosphoglycerate" type="Complex">
      <molecularWeight unit="dalton">47099.99</molecularWeight>
      <initialConcentration unit="mM">0</initialConcentration>
    </molecule>
    <molecule compartment="cell" id="ENO2+PEP" name="ENO2+phosphoenolpyruvate" type="Complex">
      <molecularWeight unit="dalton">47080.98</molecularWeight>
      <initialConcentration unit="mM">0</initialConcentration>
    </molecule>
    <!--Reaction: Fructosebisphosphate aldolase-->
    <!--enzyme - UniProt name: Fructose-bisphosphate_aldolase; resource: https://www.uniprot.org/uniprot/P14540 -->
    <molecule compartment="cell" id="FBA1" name="FBA1" type="Enzyme">
      <molecularWeight unit="dalton">39621.0</molecularWeight>
      <initialConcentration unit="mM">0.133839465704882</initialConcentration>
    </molecule>
    <molecule compartment="cell" id="FBA1+F16bP" name="FBA1+fructose_1,6-bisphosphate" type="Complex">
      <molecularWeight unit="dalton">39961.0</molecularWeight>
      <initialConcentration unit="mM">0</initialConcentration>
    </molecule>
    <molecule compartment="cell" id="FBA1+DHAP" name="FBA1+dihydroxyacetone_phosphate" type="Complex">
      <molecularWeight unit="dalton">39791.0</molecularWeight>
      <initialConcentration unit="mM">0</initialConcentration>
    </molecule>
    <molecule compartment="cell" id="FBA1+GAP" name="FBA1+glyceraldehyde_3-phosphate" type="Complex">
      <molecularWeight unit="dalton">39791.0</molecularWeight>
      <initialConcentration unit="mM">0</initialConcentration>
    </molecule>
```

```
    <molecule compartment="cell" id="FBA1+DHAP+GAP" name="FBA1+dihydroxyacetone_phosphate+glyceraldehyde_3-phosphate" type="Complex">
      <molecularWeight unit="dalton">39961.0</molecularWeight>
      <initialConcentration unit="mM">0</initialConcentration>
    </molecule>
    <!--Reaction: Hexokinase [GLK1]-->
    <!--enzyme - UniProt name: Glucokinase-1; resource: https://www.uniprot.org/uniprot/P17709 -->
    <molecule compartment="cell" id="GLK1" name="GLK1" type="Enzyme">
      <molecularWeight unit="dalton">55377.0</molecularWeight>
      <initialConcentration unit="mM">0.0450869624419226</initialConcentration>
    </molecule>
    <molecule compartment="cell" id="GLK1+GLC" name="GLK1+glucose" type="Complex">
      <molecularWeight unit="dalton">55557.06</molecularWeight>
      <initialConcentration unit="mM">0</initialConcentration>
    </molecule>
    <molecule compartment="cell" id="GLK1+ATP" name="GLK1+ATP" type="Complex">
      <molecularWeight unit="dalton">55884.0</molecularWeight>
      <initialConcentration unit="mM">0</initialConcentration>
    </molecule>
    <molecule compartment="cell" id="GLK1+GLC+ATP" name="GLK1+glucose+ATP" type="Complex">
      <molecularWeight unit="dalton">56064.06</molecularWeight>
      <initialConcentration unit="mM">0</initialConcentration>
    </molecule>
    <!--Reaction: Glycerol 3-phosphate dehydrogenase-->
    <!--enzyme - UniProt name: Glycerol-3-phosphate_dehydrogenase_[NAD(+)]_1; resource: https://www.uniprot.org/uniprot/Q00055 -->
    <molecule compartment="cell" id="GPD1" name="GPD1" type="Enzyme">
      <molecularWeight unit="dalton">42869.0</molecularWeight>
      <initialConcentration unit="mM">0.0683511177089872</initialConcentration>
    </molecule>
    <molecule compartment="cell" id="GPD1+DHAP" name="GPD1+dihydroxyacetone_phosphate" type="Complex">
      <molecularWeight unit="dalton">43039.0</molecularWeight>
      <initialConcentration unit="mM">0</initialConcentration>
    </molecule>
    <molecule compartment="cell" id="GPD1+NADH" name="GPD1+NADH" type="Complex">
      <molecularWeight unit="dalton">43534.12</molecularWeight>
      <initialConcentration unit="mM">0</initialConcentration>
    </molecule>
    <molecule compartment="cell" id="GPD1+DHAP+NADH" name="GPD1+dihydroxyacetone_phosphate+NADH" type="Complex">
      <molecularWeight unit="dalton">43704.12</molecularWeight>
      <initialConcentration unit="mM">0</initialConcentration>
    </molecule>
    <molecule compartment="cell" id="GPD1+G3P" name="GPD1+glycerol_3-phosphate" type="Complex">
      <molecularWeight unit="dalton">43041.01</molecularWeight>
      <initialConcentration unit="mM">0</initialConcentration>
    </molecule>
    <molecule compartment="cell" id="GPD1+NAD" name="GPD1+NAD" type="Complex">
      <molecularWeight unit="dalton">43533.12</molecularWeight>
      <initialConcentration unit="mM">0</initialConcentration>
    </molecule>
    <molecule compartment="cell" id="GPD1+G3P+NAD" name="GPD1+glycerol_3-phosphate+NAD" type="Complex">
      <molecularWeight unit="dalton">43705.13</molecularWeight>
      <initialConcentration unit="mM">0</initialConcentration>
    </molecule>
    <!--Reaction: Glycerol 3-phosphate dehydrogenase-->
    <!--enzyme - UniProt name: Glycerol-3-phosphate_dehydrogenase_[NAD(+)]_2,_mitochondrial; resource: https://www.uniprot.org/uniprot/P41911 -->
    <molecule compartment="cell" id="GPD2" name="GPD2" type="Enzyme">
      <molecularWeight unit="dalton">49422.0</molecularWeight>
      <initialConcentration unit="mM">0.007934056664242281</initialConcentration>
    </molecule>
    <molecule compartment="cell" id="GPD2+DHAP" name="GPD2+dihydroxyacetone_phosphate" type="Complex">
      <molecularWeight unit="dalton">49592.0</molecularWeight>
      <initialConcentration unit="mM">0</initialConcentration>
    </molecule>
```

```
      <molecule compartment="cell" id="GPD2+NADH" name="GPD2+NADH" type="Complex">
        <molecularWeight unit="dalton">50087.12</molecularWeight>
        <initialConcentration unit="mM">0</initialConcentration>
      </molecule>
      <molecule compartment="cell" id="GPD2+DHAP+NADH" name="GPD2+dihydroxyacetone_phosphate+NADH"
     type="Complex">
        <molecularWeight unit="dalton">50257.12</molecularWeight>
        <initialConcentration unit="mM">0</initialConcentration>
      </molecule>
      <molecule compartment="cell" id="GPD2+G3P" name="GPD2+glycerol_3-phosphate" type="Complex">
        <molecularWeight unit="dalton">49594.01</molecularWeight>
        <initialConcentration unit="mM">0</initialConcentration>
      </molecule>
      <molecule compartment="cell" id="GPD2+NAD" name="GPD2+NAD" type="Complex">
        <molecularWeight unit="dalton">50086.12</molecularWeight>
        <initialConcentration unit="mM">0</initialConcentration>
      </molecule>
      <molecule compartment="cell" id="GPD2+G3P+NAD" name="GPD2+glycerol_3-phosphate+NAD" type="
    Complex">
        <molecularWeight unit="dalton">50258.13</molecularWeight>
        <initialConcentration unit="mM">0</initialConcentration>
      </molecule>
      <!--Reaction: Phosphoglyceromutase-->
      <!--enzyme - UniProt name: Phosphoglycerate_mutase_1; resource: https://www.uniprot.org/
    uniprot/P00950 -->
      <molecule compartment="cell" id="GPM1" name="GPM1" type="Enzyme">
        <molecularWeight unit="dalton">27609.0</molecularWeight>
        <initialConcentration unit="mM">0.73000029889707</initialConcentration>
      </molecule>
      <molecule compartment="cell" id="GPM1+P3G" name="GPM1+3-phosphoglycerate" type="Complex">
        <molecularWeight unit="dalton">27794.99</molecularWeight>
        <initialConcentration unit="mM">0</initialConcentration>
      </molecule>
      <molecule compartment="cell" id="GPM1+P2G" name="GPM1+2-phosphoglycerate" type="Complex">
        <molecularWeight unit="dalton">27794.99</molecularWeight>
        <initialConcentration unit="mM">0</initialConcentration>
      </molecule>
      <!--Reaction: Glycerol 3-phosphatase-->
      <!--enzyme - UniProt name: Glycerol-1-phosphate_phosphohydrolase_2; resource: https://www.
    uniprot.org/uniprot/P40106 -->
      <molecule compartment="cell" id="HOR2" name="HOR2" type="Enzyme">
        <molecularWeight unit="dalton">27814.0</molecularWeight>
        <initialConcentration unit="mM">0.054734695639756</initialConcentration>
      </molecule>
      <molecule compartment="cell" id="HOR2+G3P" name="HOR2+glycerol_3-phosphate" type="Complex">
        <molecularWeight unit="dalton">27986.01</molecularWeight>
        <initialConcentration unit="mM">0</initialConcentration>
      </molecule>
      <!--Reaction: Hexokinase [HXK1]-->
      <!--enzyme - UniProt name: Hexokinase-1; resource: https://www.uniprot.org/uniprot/P04806 --
    >
      <molecule compartment="cell" id="HXK1" name="HXK1" type="Enzyme">
        <molecularWeight unit="dalton">53738.0</molecularWeight>
        <initialConcentration unit="mM">0.0167807457149784</initialConcentration>
      </molecule>
      <molecule compartment="cell" id="HXK1+GLC" name="HXK1+glucose" type="Complex">
        <molecularWeight unit="dalton">53918.06</molecularWeight>
        <initialConcentration unit="mM">0</initialConcentration>
      </molecule>
      <molecule compartment="cell" id="HXK1+ATP" name="HXK1+ATP" type="Complex">
        <molecularWeight unit="dalton">54245.0</molecularWeight>
        <initialConcentration unit="mM">0</initialConcentration>
      </molecule>
      <molecule compartment="cell" id="HXK1+GLC+ATP" name="HXK1+glucose+ATP" type="Complex">
        <molecularWeight unit="dalton">54425.06</molecularWeight>
        <initialConcentration unit="mM">0</initialConcentration>
      </molecule>
      <!--Reaction: Hexokinase [HXK2]-->
      <!--enzyme - UniProt name: Hexokinase-2; resource: https://www.uniprot.org/uniprot/P04807 --
    >
```

```
      <molecule compartment="cell" id="HXK2" name="HXK2" type="Enzyme">
        <molecularWeight unit="dalton">53942.0</molecularWeight>
        <initialConcentration unit="mM">0.0613313539705155</initialConcentration>
      </molecule>
      <molecule compartment="cell" id="HXK2+GLC" name="HXK2+glucose" type="Complex">
        <molecularWeight unit="dalton">54122.06</molecularWeight>
        <initialConcentration unit="mM">0</initialConcentration>
      </molecule>
      <molecule compartment="cell" id="HXK2+ATP" name="HXK2+ATP" type="Complex">
        <molecularWeight unit="dalton">54449.0</molecularWeight>
        <initialConcentration unit="mM">0</initialConcentration>
      </molecule>
      <molecule compartment="cell" id="HXK2+GLC+ATP" name="HXK2+glucose+ATP" type="Complex">
        <molecularWeight unit="dalton">54629.06</molecularWeight>
        <initialConcentration unit="mM">0</initialConcentration>
      </molecule>
      <!--Reaction: Phosphofructokinase-->
      <!--enzyme - UniProt name: ATP-dependent_6-phosphofructokinase_subunit_alpha; resource:
    https://www.uniprot.org/uniprot/P16861 -->
      <molecule compartment="cell" id="PFK1" name="PFK1" type="Enzyme">
        <molecularWeight unit="dalton">107970.0</molecularWeight>
        <initialConcentration unit="mM">0.0467850299063124</initialConcentration>
      </molecule>
      <molecule compartment="cell" id="PFK1+ATP" name="PFK1+ATP" type="Complex">
        <molecularWeight unit="dalton">108477.0</molecularWeight>
        <initialConcentration unit="mM">0</initialConcentration>
      </molecule>
      <molecule compartment="cell" id="PFK1+F6P" name="PFK1+fructose_6-phosphate" type="Complex">
        <molecularWeight unit="dalton">108230.03</molecularWeight>
        <initialConcentration unit="mM">0</initialConcentration>
      </molecule>
      <molecule compartment="cell" id="PFK1+ATP+F6P" name="PFK1+ATP+fructose_6-phosphate" type="
    Complex">
        <molecularWeight unit="dalton">108737.03</molecularWeight>
        <initialConcentration unit="mM">0</initialConcentration>
      </molecule>
      <!--Reaction: Phosphofructokinase-->
      <!--enzyme - UniProt name: ATP-dependent_6-phosphofructokinase_subunit_beta; resource:
    https://www.uniprot.org/uniprot/P16862 -->
      <molecule compartment="cell" id="PFK2" name="PFK2" type="Enzyme">
        <molecularWeight unit="dalton">104618.0</molecularWeight>
        <initialConcentration unit="mM">0.0390366215332091</initialConcentration>
      </molecule>
      <molecule compartment="cell" id="PFK2+ATP" name="PFK2+ATP" type="Complex">
        <molecularWeight unit="dalton">105125.0</molecularWeight>
        <initialConcentration unit="mM">0</initialConcentration>
      </molecule>
      <molecule compartment="cell" id="PFK2+F6P" name="PFK2+fructose_6-phosphate" type="Complex">
        <molecularWeight unit="dalton">104878.03</molecularWeight>
        <initialConcentration unit="mM">0</initialConcentration>
      </molecule>
      <molecule compartment="cell" id="PFK2+ATP+F6P" name="PFK2+ATP+fructose_6-phosphate" type="
    Complex">
        <molecularWeight unit="dalton">105385.03</molecularWeight>
        <initialConcentration unit="mM">0</initialConcentration>
      </molecule>
      <!--Reaction: Phosphoglucose isomerase-->
      <!--enzyme - UniProt name: Glucose-6-phosphate_isomerase; resource: https://www.uniprot.org/
    uniprot/P12709 -->
      <molecule compartment="cell" id="PGI1" name="PGI1" type="Enzyme">
        <molecularWeight unit="dalton">61299.0</molecularWeight>
        <initialConcentration unit="mM">0.1382907072901</initialConcentration>
      </molecule>
      <molecule compartment="cell" id="PGI1+G6P" name="PGI1+glucose_6-phosphate" type="Complex">
        <molecularWeight unit="dalton">61559.03</molecularWeight>
        <initialConcentration unit="mM">0</initialConcentration>
      </molecule>
      <molecule compartment="cell" id="PGI1+F6P" name="PGI1+fructose_6-phosphate" type="Complex">
        <molecularWeight unit="dalton">61559.03</molecularWeight>
        <initialConcentration unit="mM">0</initialConcentration>
```

```
    </molecule>
    <!--Reaction: 3-phosphoglycerate kinase-->
    <!--enzyme - UniProt name: Phosphoglycerate_kinase; resource: https://www.uniprot.org/uniprot/P00560 -->
    <molecule compartment="cell" id="PGK1" name="PGK1" type="Enzyme">
      <molecularWeight unit="dalton">44738.0</molecularWeight>
      <initialConcentration unit="mM">0.257656912658955</initialConcentration>
    </molecule>
    <molecule compartment="cell" id="PGK1+ADP" name="PGK1+ADP" type="Complex">
      <molecularWeight unit="dalton">45165.03</molecularWeight>
      <initialConcentration unit="mM">0</initialConcentration>
    </molecule>
    <molecule compartment="cell" id="PGK1+BPG" name="PGK1+1,3-bisphosphoglycerate" type="Complex">
      <molecularWeight unit="dalton">45003.96</molecularWeight>
      <initialConcentration unit="mM">0</initialConcentration>
    </molecule>
    <molecule compartment="cell" id="PGK1+ADP+BPG" name="PGK1+ADP+1,3-bisphosphoglycerate" type="Complex">
      <molecularWeight unit="dalton">45430.99</molecularWeight>
      <initialConcentration unit="mM">0</initialConcentration>
    </molecule>
    <molecule compartment="cell" id="PGK1+ATP" name="PGK1+ATP" type="Complex">
      <molecularWeight unit="dalton">45245.0</molecularWeight>
      <initialConcentration unit="mM">0</initialConcentration>
    </molecule>
    <molecule compartment="cell" id="PGK1+P3G" name="PGK1+3-phosphoglycerate" type="Complex">
      <molecularWeight unit="dalton">44923.99</molecularWeight>
      <initialConcentration unit="mM">0</initialConcentration>
    </molecule>
    <molecule compartment="cell" id="PGK1+ATP+P3G" name="PGK1+ATP+3-phosphoglycerate" type="Complex">
      <molecularWeight unit="dalton">45430.99</molecularWeight>
      <initialConcentration unit="mM">0</initialConcentration>
    </molecule>
    <!--Reaction: Phosphoglucomutase-->
    <!--enzyme - UniProt name: Phosphoglucomutase_1; resource: https://www.uniprot.org/uniprot/P33401 -->
    <molecule compartment="cell" id="PGM1" name="PGM1" type="Enzyme">
      <molecularWeight unit="dalton">63112.0</molecularWeight>
      <initialConcentration unit="mM">0.0326229546307459</initialConcentration>
    </molecule>
    <molecule compartment="cell" id="PGM1+G6P" name="PGM1+glucose_6-phosphate" type="Complex">
      <molecularWeight unit="dalton">63372.03</molecularWeight>
      <initialConcentration unit="mM">0</initialConcentration>
    </molecule>
    <molecule compartment="cell" id="PGM1+G1P" name="PGM1+glucose_1-phosphate" type="Complex">
      <molecularWeight unit="dalton">63372.03</molecularWeight>
      <initialConcentration unit="mM">0</initialConcentration>
    </molecule>
    <!--Reaction: Phosphoglucomutase-->
    <!--enzyme - UniProt name: Phosphoglucomutase_2; resource: https://www.uniprot.org/uniprot/P37012 -->
    <molecule compartment="cell" id="PGM2" name="PGM2" type="Enzyme">
      <molecularWeight unit="dalton">63089.0</molecularWeight>
      <initialConcentration unit="mM">0.0125868877176552</initialConcentration>
    </molecule>
    <molecule compartment="cell" id="PGM2+G6P" name="PGM2+glucose_6-phosphate" type="Complex">
      <molecularWeight unit="dalton">63349.03</molecularWeight>
      <initialConcentration unit="mM">0</initialConcentration>
    </molecule>
    <molecule compartment="cell" id="PGM2+G1P" name="PGM2+glucose_1-phosphate" type="Complex">
      <molecularWeight unit="dalton">63349.03</molecularWeight>
      <initialConcentration unit="mM">0</initialConcentration>
    </molecule>
    <!--Reaction: Pyruvate kinase [PYK2]-->
    <!--enzyme - UniProt name: Pyruvate_kinase_2; resource: https://www.uniprot.org/uniprot/P52489 -->
    <molecule compartment="cell" id="PYK2" name="PYK2" type="Enzyme">
      <molecularWeight unit="dalton">55195.0</molecularWeight>
```

```
        <initialConcentration unit="mM">0.0606993527217899</initialConcentration>
      </molecule>
      <molecule compartment="cell" id="PYK2+ADP" name="PYK2+ADP" type="Complex">
        <molecularWeight unit="dalton">55622.03</molecularWeight>
        <initialConcentration unit="mM">0</initialConcentration>
      </molecule>
      <molecule compartment="cell" id="PYK2+PEP" name="PYK2+phosphoenolpyruvate" type="Complex">
        <molecularWeight unit="dalton">55361.98</molecularWeight>
        <initialConcentration unit="mM">0</initialConcentration>
      </molecule>
      <molecule compartment="cell" id="PYK2+ADP+PEP" name="PYK2+ADP+phosphoenolpyruvate" type="
    Complex">
        <molecularWeight unit="dalton">55789.01</molecularWeight>
        <initialConcentration unit="mM">0</initialConcentration>
      </molecule>
      <!--Reaction: Glycerol 3-phosphatase-->
      <!--enzyme - UniProt name: Glycerol-1-phosphate_phosphohydrolase_1; resource: https://www.
    uniprot.org/uniprot/P41277 -->
      <molecule compartment="cell" id="RHR2" name="RHR2" type="Enzyme">
        <molecularWeight unit="dalton">27947.0</molecularWeight>
        <initialConcentration unit="mM">0.0511804773718313</initialConcentration>
      </molecule>
      <molecule compartment="cell" id="RHR2+G3P" name="RHR2+glycerol_3-phosphate" type="Complex">
        <molecularWeight unit="dalton">28119.01</molecularWeight>
        <initialConcentration unit="mM">0</initialConcentration>
      </molecule>
      <!--Reaction: Glyceraldehyde phosphate dehydrogenase [TDH1]-->
      <!--enzyme - UniProt name: Glyceraldehyde-3-phosphate_dehydrogenase_1; resource: https://www
    .uniprot.org/uniprot/P00360 -->
      <molecule compartment="cell" id="TDH1" name="TDH1" type="Enzyme">
        <molecularWeight unit="dalton">35750.0</molecularWeight>
        <initialConcentration unit="mM">0.350864642801396</initialConcentration>
      </molecule>
      <molecule compartment="cell" id="TDH1+GAP" name="TDH1+glyceraldehyde_3-phosphate" type="
    Complex">
        <molecularWeight unit="dalton">35920.0</molecularWeight>
        <initialConcentration unit="mM">0</initialConcentration>
      </molecule>
      <molecule compartment="cell" id="TDH1+NAD" name="TDH1+NAD" type="Complex">
        <molecularWeight unit="dalton">36414.12</molecularWeight>
        <initialConcentration unit="mM">0</initialConcentration>
      </molecule>
      <molecule compartment="cell" id="TDH1+GAP+NAD" name="TDH1+glyceraldehyde_3-phosphate+NAD"
    type="Complex">
        <molecularWeight unit="dalton">36584.12</molecularWeight>
        <initialConcentration unit="mM">0</initialConcentration>
      </molecule>
      <molecule compartment="cell" id="TDH1+BPG" name="TDH1+1,3-bisphosphoglycerate" type="Complex
    ">
        <molecularWeight unit="dalton">36015.96</molecularWeight>
        <initialConcentration unit="mM">0</initialConcentration>
      </molecule>
      <molecule compartment="cell" id="TDH1+NADH" name="TDH1+NADH" type="Complex">
        <molecularWeight unit="dalton">36415.12</molecularWeight>
        <initialConcentration unit="mM">0</initialConcentration>
      </molecule>
      <molecule compartment="cell" id="TDH1+BPG+NADH" name="TDH1+1,3-bisphosphoglycerate+NADH"
    type="Complex">
        <molecularWeight unit="dalton">36681.08</molecularWeight>
        <initialConcentration unit="mM">0</initialConcentration>
      </molecule>
      <!--Reaction: Glyceraldehyde phosphate dehydrogenase [TDH2]-->
      <!--enzyme - UniProt name: Glyceraldehyde-3-phosphate_dehydrogenase_2; resource: https://www
    .uniprot.org/uniprot/P00358 -->
      <molecule compartment="cell" id="TDH2" name="TDH2" type="Enzyme">
        <molecularWeight unit="dalton">35847.0</molecularWeight>
        <initialConcentration unit="mM">0</initialConcentration>
      </molecule>
      <molecule compartment="cell" id="TDH2+GAP" name="TDH2+glyceraldehyde_3-phosphate" type="
    Complex">
```

```
        <molecularWeight unit="dalton">36017.0</molecularWeight>
        <initialConcentration unit="mM">0</initialConcentration>
      </molecule>
      <molecule compartment="cell" id="TDH2+NAD" name="TDH2+NAD" type="Complex">
        <molecularWeight unit="dalton">36511.12</molecularWeight>
        <initialConcentration unit="mM">0</initialConcentration>
      </molecule>
      <molecule compartment="cell" id="TDH2+GAP+NAD" name="TDH2+glyceraldehyde_3-phosphate+NAD"
    type="Complex">
        <molecularWeight unit="dalton">36681.12</molecularWeight>
        <initialConcentration unit="mM">0</initialConcentration>
      </molecule>
      <molecule compartment="cell" id="TDH2+BPG" name="TDH2+1,3-bisphosphoglycerate" type="Complex
    ">
        <molecularWeight unit="dalton">36112.96</molecularWeight>
        <initialConcentration unit="mM">0</initialConcentration>
      </molecule>
      <molecule compartment="cell" id="TDH2+NADH" name="TDH2+NADH" type="Complex">
        <molecularWeight unit="dalton">36512.12</molecularWeight>
        <initialConcentration unit="mM">0</initialConcentration>
      </molecule>
      <molecule compartment="cell" id="TDH2+BPG+NADH" name="TDH2+1,3-bisphosphoglycerate+NADH"
    type="Complex">
        <molecularWeight unit="dalton">36778.08</molecularWeight>
        <initialConcentration unit="mM">0</initialConcentration>
      </molecule>
      <!--Reaction: Glyceraldehyde phosphate dehydrogenase [TDH3]-->
      <!--enzyme - UniProt name: Glyceraldehyde-3-phosphate_dehydrogenase_3; resource: https://www
    .uniprot.org/uniprot/P00359 -->
      <molecule compartment="cell" id="TDH3" name="TDH3" type="Enzyme">
        <molecularWeight unit="dalton">35747.0</molecularWeight>
        <initialConcentration unit="mM">0.420440474648547</initialConcentration>
      </molecule>
      <molecule compartment="cell" id="TDH3+GAP" name="TDH3+glyceraldehyde_3-phosphate" type="
    Complex">
        <molecularWeight unit="dalton">35917.0</molecularWeight>
        <initialConcentration unit="mM">0</initialConcentration>
      </molecule>
      <molecule compartment="cell" id="TDH3+NAD" name="TDH3+NAD" type="Complex">
        <molecularWeight unit="dalton">36411.12</molecularWeight>
        <initialConcentration unit="mM">0</initialConcentration>
      </molecule>
      <molecule compartment="cell" id="TDH3+GAP+NAD" name="TDH3+glyceraldehyde_3-phosphate+NAD"
    type="Complex">
        <molecularWeight unit="dalton">36581.12</molecularWeight>
        <initialConcentration unit="mM">0</initialConcentration>
      </molecule>
      <molecule compartment="cell" id="TDH3+BPG" name="TDH3+1,3-bisphosphoglycerate" type="Complex
    ">
        <molecularWeight unit="dalton">36012.96</molecularWeight>
        <initialConcentration unit="mM">0</initialConcentration>
      </molecule>
      <molecule compartment="cell" id="TDH3+NADH" name="TDH3+NADH" type="Complex">
        <molecularWeight unit="dalton">36412.12</molecularWeight>
        <initialConcentration unit="mM">0</initialConcentration>
      </molecule>
      <molecule compartment="cell" id="TDH3+BPG+NADH" name="TDH3+1,3-bisphosphoglycerate+NADH"
    type="Complex">
        <molecularWeight unit="dalton">36678.08</molecularWeight>
        <initialConcentration unit="mM">0</initialConcentration>
      </molecule>
      <!--Reaction: Triosephosphate isomerase-->
      <!--enzyme - UniProt name: Triosephosphate_isomerase; resource: https://www.uniprot.org/
    uniprot/P00942 -->
      <molecule compartment="cell" id="TPI1" name="TPI1" type="Enzyme">
        <molecularWeight unit="dalton">26795.0</molecularWeight>
        <initialConcentration unit="mM">0.294357819645508</initialConcentration>
      </molecule>
      <molecule compartment="cell" id="TPI1+DHAP" name="TPI1+dihydroxyacetone_phosphate" type="
    Complex">
```

```
        <molecularWeight unit="dalton">26965.0</molecularWeight>
        <initialConcentration unit="mM">0</initialConcentration>
      </molecule>
      <molecule compartment="cell" id="TPI1+GAP" name="TPI1+glyceraldehyde_3-phosphate" type="
    Complex">
        <molecularWeight unit="dalton">26965.0</molecularWeight>
        <initialConcentration unit="mM">0</initialConcentration>
      </molecule>
      <!--Reaction: T6P synthase-->
      <!--enzyme - UniProt name: Alpha,alpha-trehalose-phosphate_synthase_[UDP-forming]
    _56_kDa_subunit; resource: https://www.uniprot.org/uniprot/Q00764 -->
      <molecule compartment="cell" id="TPS1" name="TPS1" type="Enzyme">
        <molecularWeight unit="dalton">56148.0</molecularWeight>
        <initialConcentration unit="mM">0.033924817423706496</initialConcentration>
      </molecule>
      <molecule compartment="cell" id="TPS1+G6P" name="TPS1+glucose_6-phosphate" type="Complex">
        <molecularWeight unit="dalton">56408.03</molecularWeight>
        <initialConcentration unit="mM">0</initialConcentration>
      </molecule>
      <molecule compartment="cell" id="TPS1+UDG" name="TPS1+UDP_glucose" type="Complex">
        <molecularWeight unit="dalton">56714.06</molecularWeight>
        <initialConcentration unit="mM">0</initialConcentration>
      </molecule>
      <molecule compartment="cell" id="TPS1+G6P+UDG" name="TPS1+glucose_6-phosphate+UDP_glucose"
    type="Complex">
        <molecularWeight unit="dalton">56974.09</molecularWeight>
        <initialConcentration unit="mM">0</initialConcentration>
      </molecule>
      <!--Reaction: T6P phosphatase-->
      <!--enzyme - UniProt name: Trehalose-phosphatase; resource: https://www.uniprot.org/uniprot/
    P31688 -->
      <molecule compartment="cell" id="TPS2" name="TPS2" type="Enzyme">
        <molecularWeight unit="dalton">102976.0</molecularWeight>
        <initialConcentration unit="mM">0.0265985181347494</initialConcentration>
      </molecule>
      <molecule compartment="cell" id="TPS2+T6P" name="TPS2+trehalose_6-phosphate" type="Complex">
        <molecularWeight unit="dalton">103398.08</molecularWeight>
        <initialConcentration unit="mM">0</initialConcentration>
      </molecule>
      <!--Reaction: UDP glucose phosphorylase-->
      <!--enzyme - UniProt name: UTP- -glucose-1-phosphate_uridylyltransferase; resource: https://
    www.uniprot.org/uniprot/P32861 -->
      <molecule compartment="cell" id="UGP1" name="UGP1" type="Enzyme">
        <molecularWeight unit="dalton">55988.0</molecularWeight>
        <initialConcentration unit="mM">0.0620211419860714</initialConcentration>
      </molecule>
      <molecule compartment="cell" id="UGP1+G1P" name="UGP1+glucose_1-phosphate" type="Complex">
        <molecularWeight unit="dalton">56248.03</molecularWeight>
        <initialConcentration unit="mM">0</initialConcentration>
      </molecule>
      <molecule compartment="cell" id="UGP1+UTP" name="UGP1+UTP" type="Complex">
        <molecularWeight unit="dalton">56467.94</molecularWeight>
        <initialConcentration unit="mM">0</initialConcentration>
      </molecule>
      <molecule compartment="cell" id="UGP1+G1P+UTP" name="UGP1+glucose_1-phosphate+UTP" type="
    Complex">
        <molecularWeight unit="dalton">56727.97</molecularWeight>
        <initialConcentration unit="mM">0</initialConcentration>
      </molecule>
    </listOfMolecules>
    <listOfReactions>
      <!--Pyruvate kinase [CDC19]: irreversible reaction - forward direction-->
      <reaction>
        <interaction>
          <reactants>
            <reactant id="CDC19"/>
            <reactant id="ADP"/>
          </reactants>
          <products>
            <product id="CDC19+ADP"/>
```

```
          </products>
          <Km unit="mM">0.243</Km>
        </interaction>
        <interaction>
          <reactants>
            <reactant id="CDC19"/>
            <reactant id="PEP"/>
          </reactants>
          <products>
            <product id="CDC19+PEP"/>
          </products>
          <Km unit="mM">0.281</Km>
        </interaction>
        <interaction>
          <reactants>
            <reactant id="CDC19+ADP"/>
            <reactant id="PEP"/>
          </reactants>
          <products>
            <product id="CDC19+ADP+PEP"/>
          </products>
          <Km unit="mM">0.281</Km>
        </interaction>
        <interaction>
          <reactants>
            <reactant id="CDC19+PEP"/>
            <reactant id="ADP"/>
          </reactants>
          <products>
            <product id="CDC19+ADP+PEP"/>
          </products>
          <Km unit="mM">0.243</Km>
        </interaction>
        <interaction>
          <reactants>
            <reactant id="CDC19+ADP+PEP"/>
          </reactants>
          <products>
            <product id="ATP"/>
            <product id="PYR"/>
            <product id="CDC19"/>
          </products>
          <Km unit="mM">0.0</Km>
        </interaction>
        <kcat unit="per_second">20.146</kcat>
      </reaction>
      <!--Enolase [ENO1]: reversible reaction - forward direction-->
      <reaction>
        <interaction>
          <reactants>
            <reactant id="ENO1"/>
            <reactant id="P2G"/>
          </reactants>
          <products>
            <product id="ENO1+P2G"/>
          </products>
          <Km unit="mM">0.043</Km>
        </interaction>
        <interaction>
          <reactants>
            <reactant id="ENO1+P2G"/>
          </reactants>
          <products>
            <product id="PEP"/>
            <product id="ENO1"/>
          </products>
          <Km unit="mM">0.0</Km>
        </interaction>
        <kcat unit="per_second">7.6</kcat>
      </reaction>
```

```
<!--Enolase [ENO1]: reversible reaction - backward direction-->
<reaction>
  <interaction>
    <reactants>
      <reactant id="ENO1"/>
      <reactant id="PEP"/>
    </reactants>
    <products>
      <product id="ENO1+PEP"/>
    </products>
    <Km unit="mM">0.5</Km>
  </interaction>
  <interaction>
    <reactants>
      <reactant id="ENO1+PEP"/>
    </reactants>
    <products>
      <product id="P2G"/>
      <product id="ENO1"/>
    </products>
    <Km unit="mM">0.0</Km>
  </interaction>
  <kcat unit="per_second">7.6</kcat>
</reaction>
<!--Enolase [ENO2]: reversible reaction - forward direction-->
<reaction>
  <interaction>
    <reactants>
      <reactant id="ENO2"/>
      <reactant id="P2G"/>
    </reactants>
    <products>
      <product id="ENO2+P2G"/>
    </products>
    <Km unit="mM">0.104</Km>
  </interaction>
  <interaction>
    <reactants>
      <reactant id="ENO2+P2G"/>
    </reactants>
    <products>
      <product id="PEP"/>
      <product id="ENO2"/>
    </products>
    <Km unit="mM">0.0</Km>
  </interaction>
  <kcat unit="per_second">19.87</kcat>
</reaction>
<!--Enolase [ENO2]: reversible reaction - backward direction-->
<reaction>
  <interaction>
    <reactants>
      <reactant id="ENO2"/>
      <reactant id="PEP"/>
    </reactants>
    <products>
      <product id="ENO2+PEP"/>
    </products>
    <Km unit="mM">0.5</Km>
  </interaction>
  <interaction>
    <reactants>
      <reactant id="ENO2+PEP"/>
    </reactants>
    <products>
      <product id="P2G"/>
      <product id="ENO2"/>
    </products>
    <Km unit="mM">0.0</Km>
  </interaction>
```

```
        <kcat unit="per_second">19.87</kcat>
      </reaction>
      <!--Fructosebisphosphate aldolase: reversible reaction - forward direction-->
      <reaction>
        <interaction>
          <reactants>
            <reactant id="FBA1"/>
            <reactant id="F16bP"/>
          </reactants>
          <products>
            <product id="FBA1+F16bP"/>
          </products>
          <Km unit="mM">0.4507</Km>
        </interaction>
        <interaction>
          <reactants>
            <reactant id="FBA1+F16bP"/>
          </reactants>
          <products>
            <product id="DHAP"/>
            <product id="GAP"/>
            <product id="FBA1"/>
          </products>
          <Km unit="mM">0.0</Km>
        </interaction>
        <kcat unit="per_second">4.139</kcat>
      </reaction>
      <!--Fructosebisphosphate aldolase: reversible reaction - backward direction-->
      <reaction>
        <interaction>
          <reactants>
            <reactant id="FBA1"/>
            <reactant id="DHAP"/>
          </reactants>
          <products>
            <product id="FBA1+DHAP"/>
          </products>
          <Km unit="mM">2.0</Km>
        </interaction>
        <interaction>
          <reactants>
            <reactant id="FBA1"/>
            <reactant id="GAP"/>
          </reactants>
          <products>
            <product id="FBA1+GAP"/>
          </products>
          <Km unit="mM">2.4</Km>
        </interaction>
        <interaction>
          <reactants>
            <reactant id="FBA1+DHAP"/>
            <reactant id="GAP"/>
          </reactants>
          <products>
            <product id="FBA1+DHAP+GAP"/>
          </products>
          <Km unit="mM">2.4</Km>
        </interaction>
        <interaction>
          <reactants>
            <reactant id="FBA1+GAP"/>
            <reactant id="DHAP"/>
          </reactants>
          <products>
            <product id="FBA1+DHAP+GAP"/>
          </products>
          <Km unit="mM">2.0</Km>
        </interaction>
        <interaction>
```

```
          <reactants>
            <reactant id="FBA1+DHAP+GAP"/>
          </reactants>
          <products>
            <product id="F16bP"/>
            <product id="FBA1"/>
          </products>
          <Km unit="mM">0.0</Km>
        </interaction>
        <kcat unit="per_second">4.139</kcat>
      </reaction>
      <!--Hexokinase [GLK1]: irreversible reaction - forward direction-->
      <reaction>
        <interaction>
          <reactants>
            <reactant id="GLK1"/>
            <reactant id="GLC"/>
          </reactants>
          <products>
            <product id="GLK1+GLC"/>
          </products>
          <Km unit="mM">0.0106</Km>
        </interaction>
        <interaction>
          <reactants>
            <reactant id="GLK1"/>
            <reactant id="ATP"/>
          </reactants>
          <products>
            <product id="GLK1+ATP"/>
          </products>
          <Km unit="mM">0.865</Km>
        </interaction>
        <interaction>
          <reactants>
            <reactant id="GLK1+GLC"/>
            <reactant id="ATP"/>
          </reactants>
          <products>
            <product id="GLK1+GLC+ATP"/>
          </products>
          <Km unit="mM">0.865</Km>
        </interaction>
        <interaction>
          <reactants>
            <reactant id="GLK1+ATP"/>
            <reactant id="GLC"/>
          </reactants>
          <products>
            <product id="GLK1+GLC+ATP"/>
          </products>
          <Km unit="mM">0.0106</Km>
        </interaction>
        <interaction>
          <reactants>
            <reactant id="GLK1+GLC+ATP"/>
          </reactants>
          <products>
            <product id="G6P"/>
            <product id="ADP"/>
            <product id="GLK1"/>
          </products>
          <Km unit="mM">0.0</Km>
        </interaction>
        <kcat unit="per_second">0.0721</kcat>
      </reaction>
      <!--Glycerol 3-phosphate dehydrogenase: reversible reaction - forward direction-->
      <reaction>
        <interaction>
          <reactants>
```

```
            <reactant id="GPD1"/>
            <reactant id="DHAP"/>
          </reactants>
          <products>
            <product id="GPD1+DHAP"/>
          </products>
          <Km unit="mM">0.54</Km>
        </interaction>
        <interaction>
          <reactants>
            <reactant id="GPD1"/>
            <reactant id="NADH"/>
          </reactants>
          <products>
            <product id="GPD1+NADH"/>
          </products>
          <Km unit="mM">0.023</Km>
        </interaction>
        <interaction>
          <reactants>
            <reactant id="GPD1+DHAP"/>
            <reactant id="NADH"/>
          </reactants>
          <products>
            <product id="GPD1+DHAP+NADH"/>
          </products>
          <Km unit="mM">0.023</Km>
        </interaction>
        <interaction>
          <reactants>
            <reactant id="GPD1+NADH"/>
            <reactant id="DHAP"/>
          </reactants>
          <products>
            <product id="GPD1+DHAP+NADH"/>
          </products>
          <Km unit="mM">0.54</Km>
        </interaction>
        <interaction>
          <reactants>
            <reactant id="GPD1+DHAP+NADH"/>
          </reactants>
          <products>
            <product id="G3P"/>
            <product id="NAD"/>
            <product id="GPD1"/>
          </products>
          <Km unit="mM">0.0</Km>
        </interaction>
        <kcat unit="per_second">114.60431951800193</kcat>
      </reaction>
      <!--Glycerol 3-phosphate dehydrogenase: reversible reaction - backward direction-->
      <reaction>
        <interaction>
          <reactants>
            <reactant id="GPD1"/>
            <reactant id="G3P"/>
          </reactants>
          <products>
            <product id="GPD1+G3P"/>
          </products>
          <Km unit="mM">1.2</Km>
        </interaction>
        <interaction>
          <reactants>
            <reactant id="GPD1"/>
            <reactant id="NAD"/>
          </reactants>
          <products>
            <product id="GPD1+NAD"/>
```

```
          </products>
          <Km unit="mM">0.93</Km>
        </interaction>
        <interaction>
          <reactants>
            <reactant id="GPD1+G3P" />
            <reactant id="NAD" />
          </reactants>
          <products>
            <product id="GPD1+G3P+NAD" />
          </products>
          <Km unit="mM">0.93</Km>
        </interaction>
        <interaction>
          <reactants>
            <reactant id="GPD1+NAD" />
            <reactant id="G3P" />
          </reactants>
          <products>
            <product id="GPD1+G3P+NAD" />
          </products>
          <Km unit="mM">1.2</Km>
        </interaction>
        <interaction>
          <reactants>
            <reactant id="GPD1+G3P+NAD" />
          </reactants>
          <products>
            <product id="DHAP" />
            <product id="NADH" />
            <product id="GPD1" />
          </products>
          <Km unit="mM">0.0</Km>
        </interaction>
        <kcat unit="per_second">114.60431951800193</kcat>
      </reaction>
      <!--Glycerol 3-phosphate dehydrogenase: reversible reaction - forward direction-->
      <reaction>
        <interaction>
          <reactants>
            <reactant id="GPD2" />
            <reactant id="DHAP" />
          </reactants>
          <products>
            <product id="GPD2+DHAP" />
          </products>
          <Km unit="mM">0.54</Km>
        </interaction>
        <interaction>
          <reactants>
            <reactant id="GPD2" />
            <reactant id="NADH" />
          </reactants>
          <products>
            <product id="GPD2+NADH" />
          </products>
          <Km unit="mM">0.023</Km>
        </interaction>
        <interaction>
          <reactants>
            <reactant id="GPD2+DHAP" />
            <reactant id="NADH" />
          </reactants>
          <products>
            <product id="GPD2+DHAP+NADH" />
          </products>
          <Km unit="mM">0.023</Km>
        </interaction>
        <interaction>
          <reactants>
```

```
            <reactant id="GPD2+NADH"/>
            <reactant id="DHAP"/>
          </reactants>
          <products>
            <product id="GPD2+DHAP+NADH"/>
          </products>
          <Km unit="mM">0.54</Km>
        </interaction>
        <interaction>
          <reactants>
            <reactant id="GPD2+DHAP+NADH"/>
          </reactants>
          <products>
            <product id="G3P"/>
            <product id="NAD"/>
            <product id="GPD2"/>
          </products>
          <Km unit="mM">0.0</Km>
        </interaction>
        <kcat unit="per_second">987.3049393051481</kcat>
      </reaction>
      <!--Glycerol 3-phosphate dehydrogenase: reversible reaction - backward direction-->
      <reaction>
        <interaction>
          <reactants>
            <reactant id="GPD2"/>
            <reactant id="G3P"/>
          </reactants>
          <products>
            <product id="GPD2+G3P"/>
          </products>
          <Km unit="mM">1.2</Km>
        </interaction>
        <interaction>
          <reactants>
            <reactant id="GPD2"/>
            <reactant id="NAD"/>
          </reactants>
          <products>
            <product id="GPD2+NAD"/>
          </products>
          <Km unit="mM">0.93</Km>
        </interaction>
        <interaction>
          <reactants>
            <reactant id="GPD2+G3P"/>
            <reactant id="NAD"/>
          </reactants>
          <products>
            <product id="GPD2+G3P+NAD"/>
          </products>
          <Km unit="mM">0.93</Km>
        </interaction>
        <interaction>
          <reactants>
            <reactant id="GPD2+NAD"/>
            <reactant id="G3P"/>
          </reactants>
          <products>
            <product id="GPD2+G3P+NAD"/>
          </products>
          <Km unit="mM">1.2</Km>
        </interaction>
        <interaction>
          <reactants>
            <reactant id="GPD2+G3P+NAD"/>
          </reactants>
          <products>
            <product id="DHAP"/>
            <product id="NADH"/>
```

```
            <product id="GPD2"/>
          </products>
          <Km unit="mM">0.0</Km>
        </interaction>
        <kcat unit="per_second">987.3049393051481</kcat>
      </reaction>
      <!--Phosphoglyceromutase: reversible reaction - forward direction-->
      <reaction>
        <interaction>
          <reactants>
            <reactant id="GPM1"/>
            <reactant id="P3G"/>
          </reactants>
          <products>
            <product id="GPM1+P3G"/>
          </products>
          <Km unit="mM">1.2</Km>
        </interaction>
        <interaction>
          <reactants>
            <reactant id="GPM1+P3G"/>
          </reactants>
          <products>
            <product id="P2G"/>
            <product id="GPM1"/>
          </products>
          <Km unit="mM">0.0</Km>
        </interaction>
        <kcat unit="per_second">400.0</kcat>
      </reaction>
      <!--Phosphoglyceromutase: reversible reaction - backward direction-->
      <reaction>
        <interaction>
          <reactants>
            <reactant id="GPM1"/>
            <reactant id="P2G"/>
          </reactants>
          <products>
            <product id="GPM1+P2G"/>
          </products>
          <Km unit="mM">1.41</Km>
        </interaction>
        <interaction>
          <reactants>
            <reactant id="GPM1+P2G"/>
          </reactants>
          <products>
            <product id="P3G"/>
            <product id="GPM1"/>
          </products>
          <Km unit="mM">0.0</Km>
        </interaction>
        <kcat unit="per_second">400.0</kcat>
      </reaction>
      <!--Glycerol 3-phosphatase: irreversible reaction - forward direction-->
      <reaction>
        <interaction>
          <reactants>
            <reactant id="HOR2"/>
            <reactant id="G3P"/>
          </reactants>
          <products>
            <product id="HOR2+G3P"/>
          </products>
          <Km unit="mM">3.5</Km>
        </interaction>
        <interaction>
          <reactants>
            <reactant id="HOR2+G3P"/>
          </reactants>
```

```
          <products>
            <product id="GLY"/>
            <product id="HOR2"/>
          </products>
          <Km unit="mM">0.0</Km>
        </interaction>
        <kcat unit="per_second">161.38453370547887</kcat>
      </reaction>
      <!--Hexokinase [HXK1]: irreversible reaction - forward direction-->
      <reaction>
        <interaction>
          <reactants>
            <reactant id="HXK1"/>
            <reactant id="GLC"/>
          </reactants>
          <products>
            <product id="HXK1+GLC"/>
          </products>
          <Km unit="mM">0.15</Km>
        </interaction>
        <interaction>
          <reactants>
            <reactant id="HXK1"/>
            <reactant id="ATP"/>
          </reactants>
          <products>
            <product id="HXK1+ATP"/>
          </products>
          <Km unit="mM">0.293</Km>
        </interaction>
        <interaction>
          <reactants>
            <reactant id="HXK1+GLC"/>
            <reactant id="ATP"/>
          </reactants>
          <products>
            <product id="HXK1+GLC+ATP"/>
          </products>
          <Km unit="mM">0.293</Km>
        </interaction>
        <interaction>
          <reactants>
            <reactant id="HXK1+ATP"/>
            <reactant id="GLC"/>
          </reactants>
          <products>
            <product id="HXK1+GLC+ATP"/>
          </products>
          <Km unit="mM">0.15</Km>
        </interaction>
        <interaction>
          <reactants>
            <reactant id="HXK1+GLC+ATP"/>
          </reactants>
          <products>
            <product id="G6P"/>
            <product id="ADP"/>
            <product id="HXK1"/>
          </products>
          <Km unit="mM">0.0</Km>
        </interaction>
        <kcat unit="per_second">10.2</kcat>
      </reaction>
      <!--Hexokinase [HXK2]: irreversible reaction - forward direction-->
      <reaction>
        <interaction>
          <reactants>
            <reactant id="HXK2"/>
            <reactant id="GLC"/>
          </reactants>
```

```
          <products>
            <product id="HXK2+GLC"/>
          </products>
          <Km unit="mM">0.2</Km>
        </interaction>
        <interaction>
          <reactants>
            <reactant id="HXK2"/>
            <reactant id="ATP"/>
          </reactants>
          <products>
            <product id="HXK2+ATP"/>
          </products>
          <Km unit="mM">0.195</Km>
        </interaction>
        <interaction>
          <reactants>
            <reactant id="HXK2+GLC"/>
            <reactant id="ATP"/>
          </reactants>
          <products>
            <product id="HXK2+GLC+ATP"/>
          </products>
          <Km unit="mM">0.195</Km>
        </interaction>
        <interaction>
          <reactants>
            <reactant id="HXK2+ATP"/>
            <reactant id="GLC"/>
          </reactants>
          <products>
            <product id="HXK2+GLC+ATP"/>
          </products>
          <Km unit="mM">0.2</Km>
        </interaction>
        <interaction>
          <reactants>
            <reactant id="HXK2+GLC+ATP"/>
          </reactants>
          <products>
            <product id="G6P"/>
            <product id="ADP"/>
            <product id="HXK2"/>
          </products>
          <Km unit="mM">0.0</Km>
        </interaction>
        <kcat unit="per_second">63.1</kcat>
      </reaction>
      <!--Phosphofructokinase: irreversible reaction - forward direction-->
      <reaction>
        <interaction>
          <reactants>
            <reactant id="PFK1"/>
            <reactant id="ATP"/>
          </reactants>
          <products>
            <product id="PFK1+ATP"/>
          </products>
          <Km unit="mM">0.71</Km>
        </interaction>
        <interaction>
          <reactants>
            <reactant id="PFK1"/>
            <reactant id="F6P"/>
          </reactants>
          <products>
            <product id="PFK1+F6P"/>
          </products>
          <Km unit="mM">0.1</Km>
        </interaction>
```

```
        <interaction>
          <reactants>
            <reactant id="PFK1+ATP"/>
            <reactant id="F6P"/>
          </reactants>
          <products>
            <product id="PFK1+ATP+F6P"/>
          </products>
          <Km unit="mM">0.1</Km>
        </interaction>
        <interaction>
          <reactants>
            <reactant id="PFK1+F6P"/>
            <reactant id="ATP"/>
          </reactants>
          <products>
            <product id="PFK1+ATP+F6P"/>
          </products>
          <Km unit="mM">0.71</Km>
        </interaction>
        <interaction>
          <reactants>
            <reactant id="PFK1+ATP+F6P"/>
          </reactants>
          <products>
            <product id="ADP"/>
            <product id="F16bP"/>
            <product id="PFK1"/>
          </products>
          <Km unit="mM">0.0</Km>
        </interaction>
        <kcat unit="per_second">209.6</kcat>
      </reaction>
      <!--Phosphofructokinase: irreversible reaction - forward direction-->
      <reaction>
        <interaction>
          <reactants>
            <reactant id="PFK2"/>
            <reactant id="ATP"/>
          </reactants>
          <products>
            <product id="PFK2+ATP"/>
          </products>
          <Km unit="mM">0.71</Km>
        </interaction>
        <interaction>
          <reactants>
            <reactant id="PFK2"/>
            <reactant id="F6P"/>
          </reactants>
          <products>
            <product id="PFK2+F6P"/>
          </products>
          <Km unit="mM">0.1</Km>
        </interaction>
        <interaction>
          <reactants>
            <reactant id="PFK2+ATP"/>
            <reactant id="F6P"/>
          </reactants>
          <products>
            <product id="PFK2+ATP+F6P"/>
          </products>
          <Km unit="mM">0.1</Km>
        </interaction>
        <interaction>
          <reactants>
            <reactant id="PFK2+F6P"/>
            <reactant id="ATP"/>
          </reactants>
```

```
          <products>
            <product id="PFK2+ATP+F6P"/>
          </products>
          <Km unit="mM">0.71</Km>
        </interaction>
        <interaction>
          <reactants>
            <reactant id="PFK2+ATP+F6P"/>
          </reactants>
          <products>
            <product id="ADP"/>
            <product id="F16bP"/>
            <product id="PFK2"/>
          </products>
          <Km unit="mM">0.0</Km>
        </interaction>
        <kcat unit="per_second">209.6</kcat>
      </reaction>
      <!--Phosphoglucose isomerase: reversible reaction - forward direction-->
      <reaction>
        <interaction>
          <reactants>
            <reactant id="PGI1"/>
            <reactant id="G6P"/>
          </reactants>
          <products>
            <product id="PGI1+G6P"/>
          </products>
          <Km unit="mM">1.0257</Km>
        </interaction>
        <interaction>
          <reactants>
            <reactant id="PGI1+G6P"/>
          </reactants>
          <products>
            <product id="F6P"/>
            <product id="PGI1"/>
          </products>
          <Km unit="mM">0.0</Km>
        </interaction>
        <kcat unit="per_second">487.36</kcat>
      </reaction>
      <!--Phosphoglucose isomerase: reversible reaction - backward direction-->
      <reaction>
        <interaction>
          <reactants>
            <reactant id="PGI1"/>
            <reactant id="F6P"/>
          </reactants>
          <products>
            <product id="PGI1+F6P"/>
          </products>
          <Km unit="mM">0.307</Km>
        </interaction>
        <interaction>
          <reactants>
            <reactant id="PGI1+F6P"/>
          </reactants>
          <products>
            <product id="G6P"/>
            <product id="PGI1"/>
          </products>
          <Km unit="mM">0.0</Km>
        </interaction>
        <kcat unit="per_second">487.36</kcat>
      </reaction>
      <!--3-phosphoglycerate kinase: reversible reaction - forward direction-->
      <reaction>
        <interaction>
          <reactants>
```

```
            <reactant id="PGK1"/>
            <reactant id="ADP"/>
          </reactants>
          <products>
            <product id="PGK1+ADP"/>
          </products>
          <Km unit="mM">0.2</Km>
        </interaction>
        <interaction>
          <reactants>
            <reactant id="PGK1"/>
            <reactant id="BPG"/>
          </reactants>
          <products>
            <product id="PGK1+BPG"/>
          </products>
          <Km unit="mM">0.003</Km>
        </interaction>
        <interaction>
          <reactants>
            <reactant id="PGK1+ADP"/>
            <reactant id="BPG"/>
          </reactants>
          <products>
            <product id="PGK1+ADP+BPG"/>
          </products>
          <Km unit="mM">0.003</Km>
        </interaction>
        <interaction>
          <reactants>
            <reactant id="PGK1+BPG"/>
            <reactant id="ADP"/>
          </reactants>
          <products>
            <product id="PGK1+ADP+BPG"/>
          </products>
          <Km unit="mM">0.2</Km>
        </interaction>
        <interaction>
          <reactants>
            <reactant id="PGK1+ADP+BPG"/>
          </reactants>
          <products>
            <product id="ATP"/>
            <product id="P3G"/>
            <product id="PGK1"/>
          </products>
          <Km unit="mM">0.0</Km>
        </interaction>
        <kcat unit="per_second">58.6</kcat>
      </reaction>
      <!--3-phosphoglycerate kinase: reversible reaction - backward direction-->
      <reaction>
        <interaction>
          <reactants>
            <reactant id="PGK1"/>
            <reactant id="ATP"/>
          </reactants>
          <products>
            <product id="PGK1+ATP"/>
          </products>
          <Km unit="mM">1.99</Km>
        </interaction>
        <interaction>
          <reactants>
            <reactant id="PGK1"/>
            <reactant id="P3G"/>
          </reactants>
          <products>
            <product id="PGK1+P3G"/>
```

```
          </products>
          <Km unit="mM">4.58</Km>
        </interaction>
        <interaction>
          <reactants>
            <reactant id="PGK1+ATP" />
            <reactant id="P3G" />
          </reactants>
          <products>
            <product id="PGK1+ATP+P3G" />
          </products>
          <Km unit="mM">4.58</Km>
        </interaction>
        <interaction>
          <reactants>
            <reactant id="PGK1+P3G" />
            <reactant id="ATP" />
          </reactants>
          <products>
            <product id="PGK1+ATP+P3G" />
          </products>
          <Km unit="mM">1.99</Km>
        </interaction>
        <interaction>
          <reactants>
            <reactant id="PGK1+ATP+P3G" />
          </reactants>
          <products>
            <product id="ADP" />
            <product id="BPG" />
            <product id="PGK1" />
          </products>
          <Km unit="mM">0.0</Km>
        </interaction>
        <kcat unit="per_second">58.6</kcat>
      </reaction>
      <!--Phosphoglucomutase: reversible reaction - forward direction-->
      <reaction>
        <interaction>
          <reactants>
            <reactant id="PGM1" />
            <reactant id="G6P" />
          </reactants>
          <products>
            <product id="PGM1+G6P" />
          </products>
          <Km unit="mM">0.05</Km>
        </interaction>
        <interaction>
          <reactants>
            <reactant id="PGM1+G6P" />
          </reactants>
          <products>
            <product id="G1P" />
            <product id="PGM1" />
          </products>
          <Km unit="mM">0.0</Km>
        </interaction>
        <kcat unit="per_second">39.11969392242694</kcat>
      </reaction>
      <!--Phosphoglucomutase: reversible reaction - backward direction-->
      <reaction>
        <interaction>
          <reactants>
            <reactant id="PGM1" />
            <reactant id="G1P" />
          </reactants>
          <products>
            <product id="PGM1+G1P" />
          </products>
```

```
          <Km unit="mM">0.023</Km>
        </interaction>
        <interaction>
          <reactants>
            <reactant id="PGM1+G1P"/>
          </reactants>
          <products>
            <product id="G6P"/>
            <product id="PGM1"/>
          </products>
          <Km unit="mM">0.0</Km>
        </interaction>
        <kcat unit="per_second">39.11969392242694</kcat>
      </reaction>
      <!--Phosphoglucomutase: reversible reaction - forward direction-->
      <reaction>
        <interaction>
          <reactants>
            <reactant id="PGM2"/>
            <reactant id="G6P"/>
          </reactants>
          <products>
            <product id="PGM2+G6P"/>
          </products>
          <Km unit="mM">0.05</Km>
        </interaction>
        <interaction>
          <reactants>
            <reactant id="PGM2+G6P"/>
          </reactants>
          <products>
            <product id="G1P"/>
            <product id="PGM2"/>
          </products>
          <Km unit="mM">0.0</Km>
        </interaction>
        <kcat unit="per_second">101.39122781002628</kcat>
      </reaction>
      <!--Phosphoglucomutase: reversible reaction - backward direction-->
      <reaction>
        <interaction>
          <reactants>
            <reactant id="PGM2"/>
            <reactant id="G1P"/>
          </reactants>
          <products>
            <product id="PGM2+G1P"/>
          </products>
          <Km unit="mM">0.023</Km>
        </interaction>
        <interaction>
          <reactants>
            <reactant id="PGM2+G1P"/>
          </reactants>
          <products>
            <product id="G6P"/>
            <product id="PGM2"/>
          </products>
          <Km unit="mM">0.0</Km>
        </interaction>
        <kcat unit="per_second">101.39122781002628</kcat>
      </reaction>
      <!--Pyruvate kinase [PYK2]: irreversible reaction - forward direction-->
      <reaction>
        <interaction>
          <reactants>
            <reactant id="PYK2"/>
            <reactant id="ADP"/>
          </reactants>
          <products>
```

```
            <product id="PYK2+ADP"/>
          </products>
          <Km unit="mM">0.3</Km>
        </interaction>
        <interaction>
          <reactants>
            <reactant id="PYK2"/>
            <reactant id="PEP"/>
          </reactants>
          <products>
            <product id="PYK2+PEP"/>
          </products>
          <Km unit="mM">0.19</Km>
        </interaction>
        <interaction>
          <reactants>
            <reactant id="PYK2+ADP"/>
            <reactant id="PEP"/>
          </reactants>
          <products>
            <product id="PYK2+ADP+PEP"/>
          </products>
          <Km unit="mM">0.19</Km>
        </interaction>
        <interaction>
          <reactants>
            <reactant id="PYK2+PEP"/>
            <reactant id="ADP"/>
          </reactants>
          <products>
            <product id="PYK2+ADP+PEP"/>
          </products>
          <Km unit="mM">0.3</Km>
        </interaction>
        <interaction>
          <reactants>
            <reactant id="PYK2+ADP+PEP"/>
          </reactants>
          <products>
            <product id="ATP"/>
            <product id="PYR"/>
            <product id="PYK2"/>
          </products>
          <Km unit="mM">0.0</Km>
        </interaction>
        <kcat unit="per_second">0.0</kcat>
      </reaction>
      <!--Glycerol 3-phosphatase: irreversible reaction - forward direction-->
      <reaction>
        <interaction>
          <reactants>
            <reactant id="RHR2"/>
            <reactant id="G3P"/>
          </reactants>
          <products>
            <product id="RHR2+G3P"/>
          </products>
          <Km unit="mM">3.5</Km>
        </interaction>
        <interaction>
          <reactants>
            <reactant id="RHR2+G3P"/>
          </reactants>
          <products>
            <product id="GLY"/>
            <product id="RHR2"/>
          </products>
          <Km unit="mM">0.0</Km>
        </interaction>
        <kcat unit="per_second">17.25918511693097</kcat>
```

```
    </reaction>
    <!--Glyceraldehyde phosphate dehydrogenase [TDH1]: reversible reaction - forward direction-->
    <reaction>
      <interaction>
        <reactants>
          <reactant id="TDH1"/>
          <reactant id="GAP"/>
        </reactants>
        <products>
          <product id="TDH1+GAP"/>
        </products>
        <Km unit="mM">0.495</Km>
      </interaction>
      <interaction>
        <reactants>
          <reactant id="TDH1"/>
          <reactant id="NAD"/>
        </reactants>
        <products>
          <product id="TDH1+NAD"/>
        </products>
        <Km unit="mM">0.09</Km>
      </interaction>
      <interaction>
        <reactants>
          <reactant id="TDH1+GAP"/>
          <reactant id="NAD"/>
        </reactants>
        <products>
          <product id="TDH1+GAP+NAD"/>
        </products>
        <Km unit="mM">0.09</Km>
      </interaction>
      <interaction>
        <reactants>
          <reactant id="TDH1+NAD"/>
          <reactant id="GAP"/>
        </reactants>
        <products>
          <product id="TDH1+GAP+NAD"/>
        </products>
        <Km unit="mM">0.495</Km>
      </interaction>
      <interaction>
        <reactants>
          <reactant id="TDH1+GAP+NAD"/>
        </reactants>
        <products>
          <product id="BPG"/>
          <product id="NADH"/>
          <product id="TDH1"/>
        </products>
        <Km unit="mM">0.0</Km>
      </interaction>
      <kcat unit="per_second">19.12</kcat>
    </reaction>
    <!--Glyceraldehyde phosphate dehydrogenase [TDH1]: reversible reaction - backward direction-->
    <reaction>
      <interaction>
        <reactants>
          <reactant id="TDH1"/>
          <reactant id="BPG"/>
        </reactants>
        <products>
          <product id="TDH1+BPG"/>
        </products>
        <Km unit="mM">0.0098</Km>
      </interaction>
```

```
        <interaction>
          <reactants>
            <reactant id="TDH1"/>
            <reactant id="NADH"/>
          </reactants>
          <products>
            <product id="TDH1+NADH"/>
          </products>
          <Km unit="mM">0.06</Km>
        </interaction>
        <interaction>
          <reactants>
            <reactant id="TDH1+BPG"/>
            <reactant id="NADH"/>
          </reactants>
          <products>
            <product id="TDH1+BPG+NADH"/>
          </products>
          <Km unit="mM">0.06</Km>
        </interaction>
        <interaction>
          <reactants>
            <reactant id="TDH1+NADH"/>
            <reactant id="BPG"/>
          </reactants>
          <products>
            <product id="TDH1+BPG+NADH"/>
          </products>
          <Km unit="mM">0.0098</Km>
        </interaction>
        <interaction>
          <reactants>
            <reactant id="TDH1+BPG+NADH"/>
          </reactants>
          <products>
            <product id="GAP"/>
            <product id="NAD"/>
            <product id="TDH1"/>
          </products>
          <Km unit="mM">0.0</Km>
        </interaction>
        <kcat unit="per_second">19.12</kcat>
      </reaction>
      <!--Glyceraldehyde phosphate dehydrogenase [TDH2]: reversible reaction - forward direction--
    >
      <reaction>
        <interaction>
          <reactants>
            <reactant id="TDH2"/>
            <reactant id="GAP"/>
          </reactants>
          <products>
            <product id="TDH2+GAP"/>
          </products>
          <Km unit="mM">0.77</Km>
        </interaction>
        <interaction>
          <reactants>
            <reactant id="TDH2"/>
            <reactant id="NAD"/>
          </reactants>
          <products>
            <product id="TDH2+NAD"/>
          </products>
          <Km unit="mM">0.09</Km>
        </interaction>
        <interaction>
          <reactants>
            <reactant id="TDH2+GAP"/>
            <reactant id="NAD"/>
```

```
          </reactants>
          <products>
            <product id="TDH2+GAP+NAD"/>
          </products>
          <Km unit="mM">0.09</Km>
        </interaction>
        <interaction>
          <reactants>
            <reactant id="TDH2+NAD"/>
            <reactant id="GAP"/>
          </reactants>
          <products>
            <product id="TDH2+GAP+NAD"/>
          </products>
          <Km unit="mM">0.77</Km>
        </interaction>
        <interaction>
          <reactants>
            <reactant id="TDH2+GAP+NAD"/>
          </reactants>
          <products>
            <product id="BPG"/>
            <product id="NADH"/>
            <product id="TDH2"/>
          </products>
          <Km unit="mM">0.0</Km>
        </interaction>
        <kcat unit="per_second">8.633</kcat>
      </reaction>
      <!--Glyceraldehyde phosphate dehydrogenase [TDH2]: reversible reaction - backward direction
    -->
      <reaction>
        <interaction>
          <reactants>
            <reactant id="TDH2"/>
            <reactant id="BPG"/>
          </reactants>
          <products>
            <product id="TDH2+BPG"/>
          </products>
          <Km unit="mM">0.0098</Km>
        </interaction>
        <interaction>
          <reactants>
            <reactant id="TDH2"/>
            <reactant id="NADH"/>
          </reactants>
          <products>
            <product id="TDH2+NADH"/>
          </products>
          <Km unit="mM">0.06</Km>
        </interaction>
        <interaction>
          <reactants>
            <reactant id="TDH2+BPG"/>
            <reactant id="NADH"/>
          </reactants>
          <products>
            <product id="TDH2+BPG+NADH"/>
          </products>
          <Km unit="mM">0.06</Km>
        </interaction>
        <interaction>
          <reactants>
            <reactant id="TDH2+NADH"/>
            <reactant id="BPG"/>
          </reactants>
          <products>
            <product id="TDH2+BPG+NADH"/>
          </products>
```

```
          <Km unit="mM">0.0098</Km>
        </interaction>
        <interaction>
          <reactants>
            <reactant id="TDH2+BPG+NADH"/>
          </reactants>
          <products>
            <product id="GAP"/>
            <product id="NAD"/>
            <product id="TDH2"/>
          </products>
          <Km unit="mM">0.0</Km>
        </interaction>
        <kcat unit="per_second">8.633</kcat>
      </reaction>
      <!--Glyceraldehyde phosphate dehydrogenase [TDH3]: reversible reaction - forward direction--
    >
      <reaction>
        <interaction>
          <reactants>
            <reactant id="TDH3"/>
            <reactant id="GAP"/>
          </reactants>
          <products>
            <product id="TDH3+GAP"/>
          </products>
          <Km unit="mM">0.423</Km>
        </interaction>
        <interaction>
          <reactants>
            <reactant id="TDH3"/>
            <reactant id="NAD"/>
          </reactants>
          <products>
            <product id="TDH3+NAD"/>
          </products>
          <Km unit="mM">0.09</Km>
        </interaction>
        <interaction>
          <reactants>
            <reactant id="TDH3+GAP"/>
            <reactant id="NAD"/>
          </reactants>
          <products>
            <product id="TDH3+GAP+NAD"/>
          </products>
          <Km unit="mM">0.09</Km>
        </interaction>
        <interaction>
          <reactants>
            <reactant id="TDH3+NAD"/>
            <reactant id="GAP"/>
          </reactants>
          <products>
            <product id="TDH3+GAP+NAD"/>
          </products>
          <Km unit="mM">0.423</Km>
        </interaction>
        <interaction>
          <reactants>
            <reactant id="TDH3+GAP+NAD"/>
          </reactants>
          <products>
            <product id="BPG"/>
            <product id="NADH"/>
            <product id="TDH3"/>
          </products>
          <Km unit="mM">0.0</Km>
        </interaction>
        <kcat unit="per_second">18.162</kcat>
```

```
    </reaction>
    <!--Glyceraldehyde phosphate dehydrogenase [TDH3]: reversible reaction - backward direction
  -->
    <reaction>
      <interaction>
        <reactants>
          <reactant id="TDH3"/>
          <reactant id="BPG"/>
        </reactants>
        <products>
          <product id="TDH3+BPG"/>
        </products>
        <Km unit="mM">0.909</Km>
      </interaction>
      <interaction>
        <reactants>
          <reactant id="TDH3"/>
          <reactant id="NADH"/>
        </reactants>
        <products>
          <product id="TDH3+NADH"/>
        </products>
        <Km unit="mM">0.06</Km>
      </interaction>
      <interaction>
        <reactants>
          <reactant id="TDH3+BPG"/>
          <reactant id="NADH"/>
        </reactants>
        <products>
          <product id="TDH3+BPG+NADH"/>
        </products>
        <Km unit="mM">0.06</Km>
      </interaction>
      <interaction>
        <reactants>
          <reactant id="TDH3+NADH"/>
          <reactant id="BPG"/>
        </reactants>
        <products>
          <product id="TDH3+BPG+NADH"/>
        </products>
        <Km unit="mM">0.909</Km>
      </interaction>
      <interaction>
        <reactants>
          <reactant id="TDH3+BPG+NADH"/>
        </reactants>
        <products>
          <product id="GAP"/>
          <product id="NAD"/>
          <product id="TDH3"/>
        </products>
        <Km unit="mM">0.0</Km>
      </interaction>
      <kcat unit="per_second">18.162</kcat>
    </reaction>
    <!--Triosephosphate isomerase: reversible reaction - forward direction-->
    <reaction>
      <interaction>
        <reactants>
          <reactant id="TPI1"/>
          <reactant id="DHAP"/>
        </reactants>
        <products>
          <product id="TPI1+DHAP"/>
        </products>
        <Km unit="mM">6.454</Km>
      </interaction>
      <interaction>
```

```
          <reactants>
            <reactant id="TPI1+DHAP"/>
          </reactants>
          <products>
            <product id="GAP"/>
            <product id="TPI1"/>
          </products>
          <Km unit="mM">0.0</Km>
        </interaction>
        <kcat unit="per_second">564.38</kcat>
      </reaction>
      <!--Triosephosphate isomerase: reversible reaction - backward direction-->
      <reaction>
        <interaction>
          <reactants>
            <reactant id="TPI1"/>
            <reactant id="GAP"/>
          </reactants>
          <products>
            <product id="TPI1+GAP"/>
          </products>
          <Km unit="mM">5.25</Km>
        </interaction>
        <interaction>
          <reactants>
            <reactant id="TPI1+GAP"/>
          </reactants>
          <products>
            <product id="DHAP"/>
            <product id="TPI1"/>
          </products>
          <Km unit="mM">0.0</Km>
        </interaction>
        <kcat unit="per_second">564.38</kcat>
      </reaction>
      <!--T6P synthase: irreversible reaction - forward direction-->
      <reaction>
        <interaction>
          <reactants>
            <reactant id="TPS1"/>
            <reactant id="G6P"/>
          </reactants>
          <products>
            <product id="TPS1+G6P"/>
          </products>
          <Km unit="mM">3.8</Km>
        </interaction>
        <interaction>
          <reactants>
            <reactant id="TPS1"/>
            <reactant id="UDG"/>
          </reactants>
          <products>
            <product id="TPS1+UDG"/>
          </products>
          <Km unit="mM">0.886</Km>
        </interaction>
        <interaction>
          <reactants>
            <reactant id="TPS1+G6P"/>
            <reactant id="UDG"/>
          </reactants>
          <products>
            <product id="TPS1+G6P+UDG"/>
          </products>
          <Km unit="mM">0.886</Km>
        </interaction>
        <interaction>
          <reactants>
            <reactant id="TPS1+UDG"/>
```

```
            <reactant id="G6P"/>
          </reactants>
          <products>
            <product id="TPS1+G6P+UDG"/>
          </products>
          <Km unit="mM">3.8</Km>
        </interaction>
        <interaction>
          <reactants>
            <reactant id="TPS1+G6P+UDG"/>
          </reactants>
          <products>
            <product id="T6P"/>
            <product id="UDP"/>
            <product id="TPS1"/>
          </products>
          <Km unit="mM">0.0</Km>
        </interaction>
        <kcat unit="per_second">145.4864130396477</kcat>
      </reaction>
      <!--T6P phosphatase: irreversible reaction - forward direction-->
      <reaction>
        <interaction>
          <reactants>
            <reactant id="TPS2"/>
            <reactant id="T6P"/>
          </reactants>
          <products>
            <product id="TPS2+T6P"/>
          </products>
          <Km unit="mM">0.5</Km>
        </interaction>
        <interaction>
          <reactants>
            <reactant id="TPS2+T6P"/>
          </reactants>
          <products>
            <product id="TRH"/>
            <product id="TPS2"/>
          </products>
          <Km unit="mM">0.0</Km>
        </interaction>
        <kcat unit="per_second">879.7482582095118</kcat>
      </reaction>
      <!--UDP glucose phosphorylase: irreversible reaction - forward direction-->
      <reaction>
        <interaction>
          <reactants>
            <reactant id="UGP1"/>
            <reactant id="G1P"/>
          </reactants>
          <products>
            <product id="UGP1+G1P"/>
          </products>
          <Km unit="mM">0.32</Km>
        </interaction>
        <interaction>
          <reactants>
            <reactant id="UGP1"/>
            <reactant id="UTP"/>
          </reactants>
          <products>
            <product id="UGP1+UTP"/>
          </products>
          <Km unit="mM">0.11</Km>
        </interaction>
        <interaction>
          <reactants>
            <reactant id="UGP1+G1P"/>
            <reactant id="UTP"/>
```

```
          </reactants>
          <products>
            <product id="UGP1+G1P+UTP"/>
          </products>
          <Km unit="mM">0.11</Km>
        </interaction>
        <interaction>
          <reactants>
            <reactant id="UGP1+UTP"/>
            <reactant id="G1P"/>
          </reactants>
          <products>
            <product id="UGP1+G1P+UTP"/>
          </products>
          <Km unit="mM">0.32</Km>
        </interaction>
        <interaction>
          <reactants>
            <reactant id="UGP1+G1P+UTP"/>
          </reactants>
          <products>
            <product id="UDG"/>
            <product id="UGP1"/>
          </products>
          <Km unit="mM">0.0</Km>
        </interaction>
        <kcat unit="per_second">2137.20669686747</kcat>
      </reaction>
    </listOfReactions>
  </pathway>
  <!--Ambient settings-->
  <ambientSettings>
    <volumeOfSimulation unit="attolitre">1</volumeOfSimulation>
    <viscosity unit="pascal_second">0.0011</viscosity>
    <temperature unit="kelvin">298.15</temperature>
  </ambientSettings>
  <!--Interaction settings-->
  <interactionSettings>
    <perceptionDistance unit="angstrom">300</perceptionDistance>
    <priorityBySpecificity>false</priorityBySpecificity>
  </interactionSettings>
</orion>
```
