# Supplementary material for: Agent-based models for detecting the driving forces of biomolecular interactions
Source: Sci Rep. 2022 Feb 3;12:1878. doi: 10.1038/s41598-021-04205-8 (PMC8814177; doi:10.1038/s41598-021-04205-8)
Supplement: Supplementary file 2 — Supplementary Information. [file 41598_2021_4205_MOESM2_ESM.zip › manuscript_plots-dataset/README.pdf]

Simulation-generated data underlying the manuscript's plots

This folder contains the files generated by the simulations carried out during our research; they store the data underlying the plots of the concentration changes over time shown in the Results section of the manuscript and in Section 3 of the Supplementary Information.

Inside this folder, you can find the following CSV (Comma-Separated Values) files:

- `agent_based_simulation_output-5A-pbd.csv`, `agent_based_simulation_output-10A-pbd.csv` and `agent_based_simulation_output-300A-pbd.csv`, which are, respectively, the output files produced by the three types of agent-based simulations discussed in the manuscript; the perception distance of the molecules - 5 Å, 10 Å, or 300 Å - is indicated in the filename (`-pdb` stands for "probability by distance" - see the manuscript's Supplementary Information for details). Each CSV file is a simulation standard output, reporting type (metabolite, enzyme or complex), name, and concentration (in mmol/l) of every molecular species in the modelled cytoplasm portion at each instant of the simulation.
- `time_course_simulation_output.csv`, generated by performing, with COPASI [1], the time-course deterministic simulation over the modified version of the Smallbone2013 - Iteration 18 SBML [2]. The COPASI file of the modified model is available at https://bit.ly/orion-simulator (see the Results section of the manuscript for details on the changes we applied to the original model).

---

[1] Hoops, S. et al. COPASI–a COmplex PAthway SImulator. Bioinformatics 22, 3067–3074 (2006)
[2] Smallbone, K. et al. A model of yeast glycolysis based on a consistent kinetic characterisation of all its enzymes. FEBS Letters 587, 2832–2841 (2013)
